# Supplementary material for: Conservation of uORF repressiveness and sequence features in mouse, human and zebrafish
Source: Nat Commun. 2016 May 24;7:11663. doi: 10.1038/ncomms11663 (PMC4890304; doi:10.1038/ncomms11663)
Supplement: Supplementary Information — Supplementary Figures 1-14, Supplementary Tables 1-7 and Supplementary Note 1 [file ncomms11663-s1.pdf]

SUPPLEMENTARY FIGURES

All figures used reanalyzed data from mES cells (Ingolia et al. 2011) unless stated otherwise.

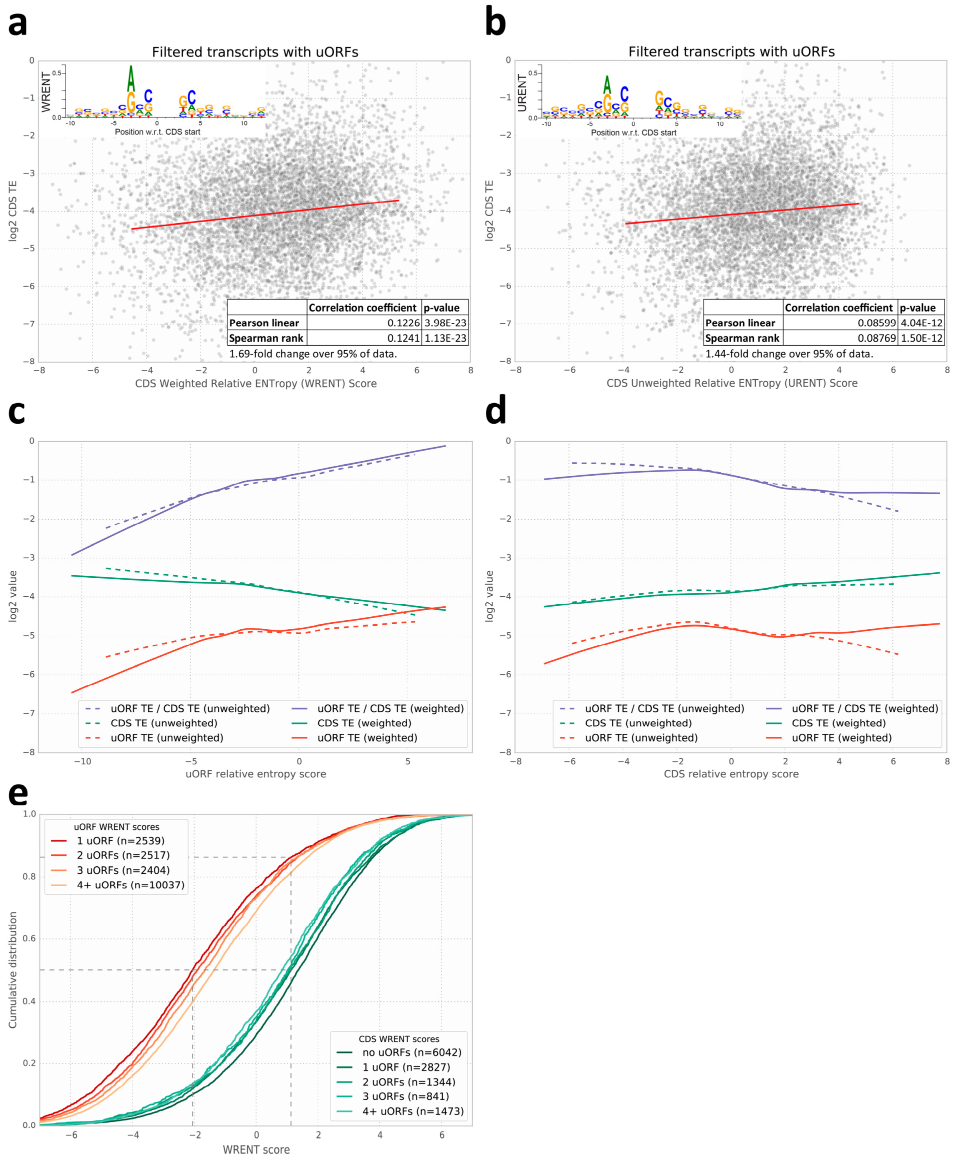

<u>**Supplementary Fig. 1:** Characterization of uORF initiation context sequences using relative entropy scores</u>

a-b. Scatter plots of CDS TE against CDS initiation context relative entropy score (weighted **(a)** and unweighted **(b)**; WRENT and URENT respectively), in transcripts with uORFs. The respective relative entropy scoring matrices are depicted as sequence logos in figure insets.

c. Relationship of uORF unweighted (URENT; dashed lines) and weighted (WRENT; solid lines) relative entropy scores to uORF repressiveness, CDS TE and uORF TE; only the LOWESS trends are shown.

d. Relationship of CDS unweighted (URENT; dashed lines) and weighted (WRENT; solid lines) relative entropy scores to uORF repressiveness, CDS TE and uORF TE; only the LOWESS trends are shown.

e. WRENT scores of uORF and CDS AUGs, grouped by the number of uORFs in each transcript, visualized as a cumulative distribution plot. Dashed lines indicate median uORF and CDS WRENT scores for transcripts with only one uORF, as well as the proportion of uORFs (~86%) with WRENT scores less than the median CDS WRENT score.

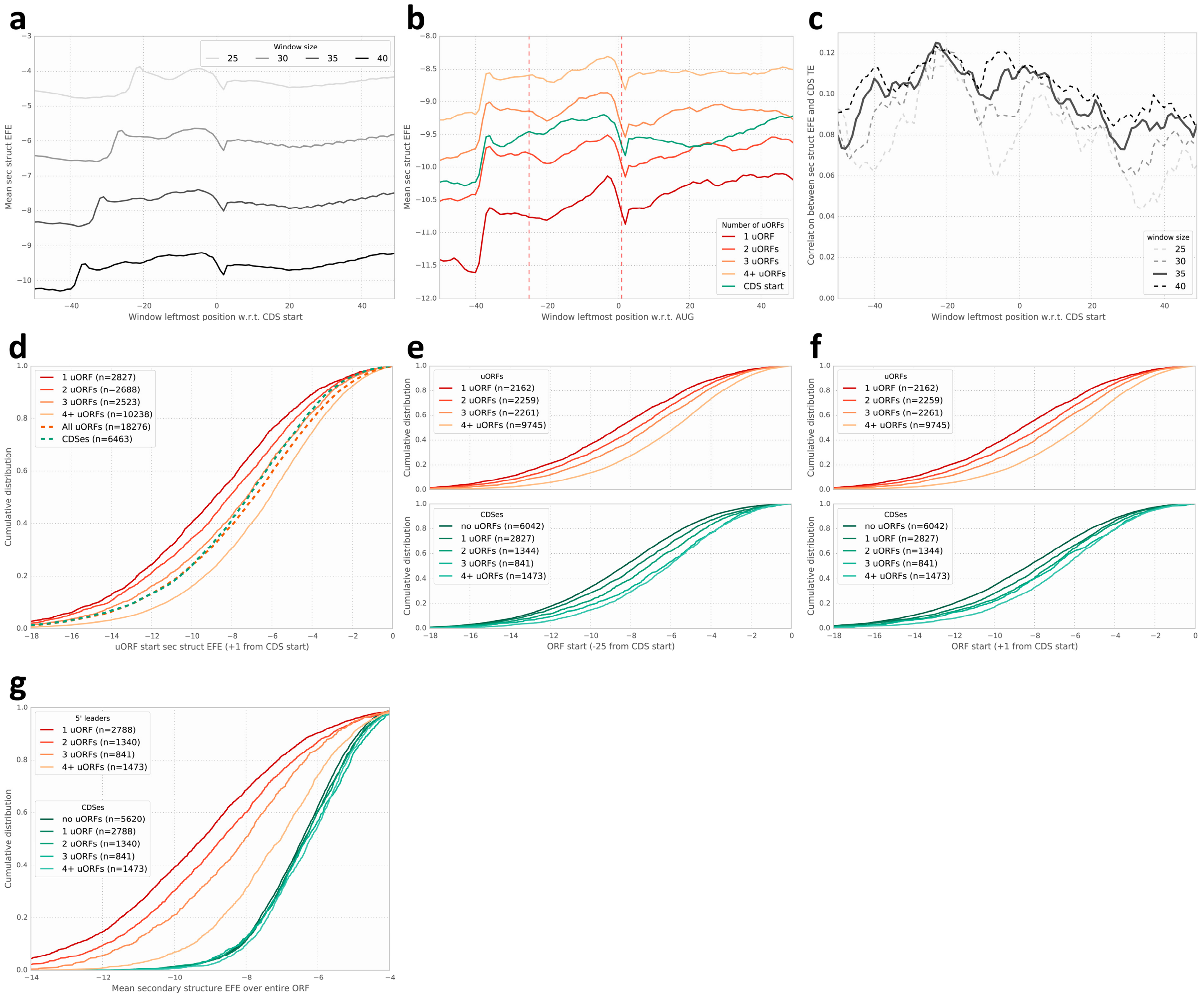

Supplementary Fig. 2: Characterization of initiation context secondary structure using ensemble free energy (EFE)

a. Mean secondary structure EFE profiles around CDS starts using sliding windows of 25, 30, 35 and 40 nucleotides.

b. Mean secondary structure EFE profiles over uORF and CDS starts, for transcripts with varying number of uORFs. Red lines indicate where correlation with CDS TE (see panel **c**) is locally maximal.

c. Correlation between secondary structure EFE and CDS TE for various positions around the CDS start, for varying sliding window sizes. Solid line corresponds to 35 nucleotide window.

d. Cumulative distribution of initiation context secondary structure (at position -25 from the ORF start) of uORFs in transcripts with varying number of uORFs. Distribution of secondary structure EFEs for all uORFs and CDS initiation contexts are indicated (dashed lines).

e-f. Cumulative distribution plot of initiation context secondary structure (at positions -25 **(e)** and +1 **(f)** from the ORF start) of uORFs (above) and CDSes (below)) in transcripts with varying number of uORFs.

g. Cumulative distribution plots of mean secondary structure (folded in 35 nucleotide windows) over entire 5' leaders and CDSes in transcripts with varying number of uORFs.

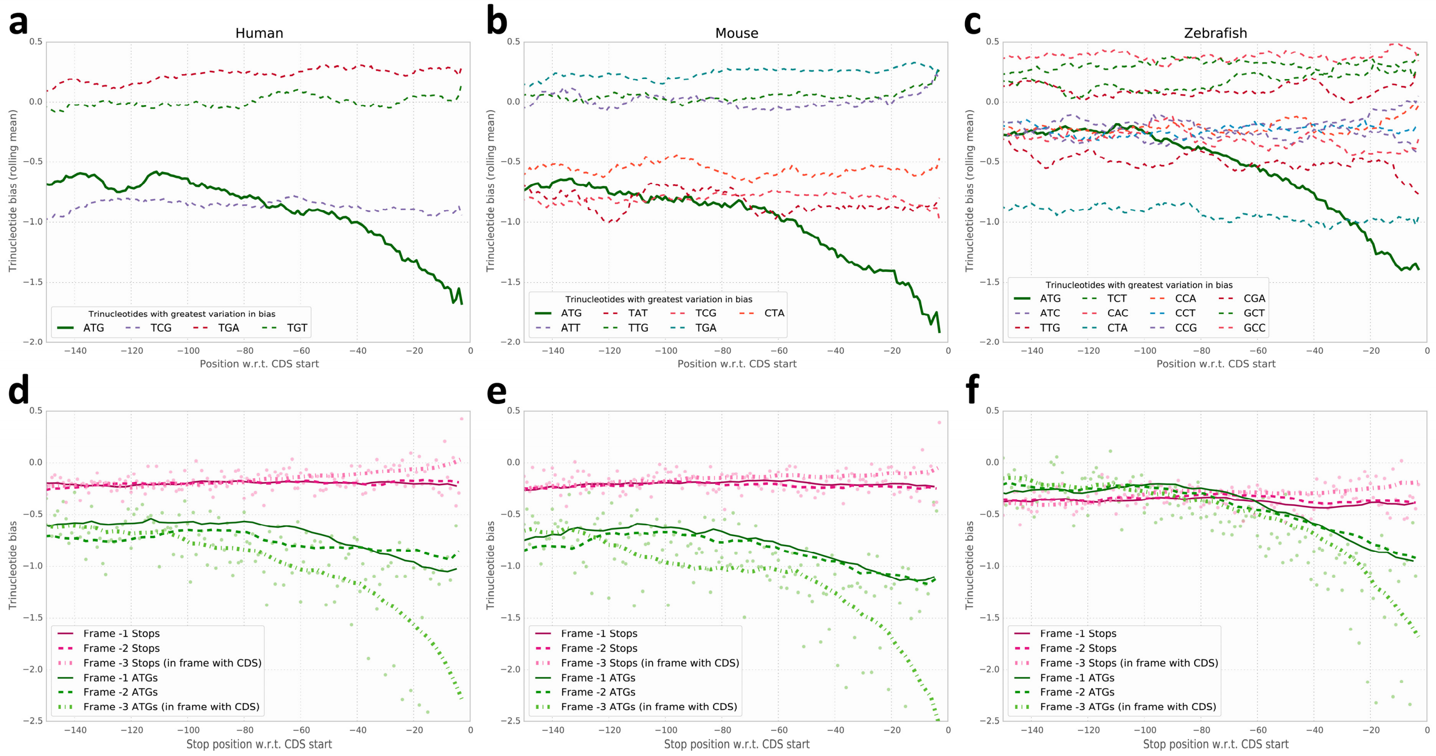

Supplementary Fig. 3: Characterization of position-specific depletion of trinuclotides in 5' leader in vertebrates

a-c. Trinucleotide biases (moving average over 15 nucleotides) comparing start codons (thick solid green line) with a selection of other codons (dashed lines) with large variations in bias: **(a)** human, **(b)** mouse, **(c)** zebrafish.

d-f. Trinucleotide biases of start and stop codons in 3 different frames for all 3 vertebrates: **(d)** human, **(e)** mouse, **(f)** zebrafish. Moving averages over 15 nucleotides are plotted as lines, while the raw values are scatter plotted.

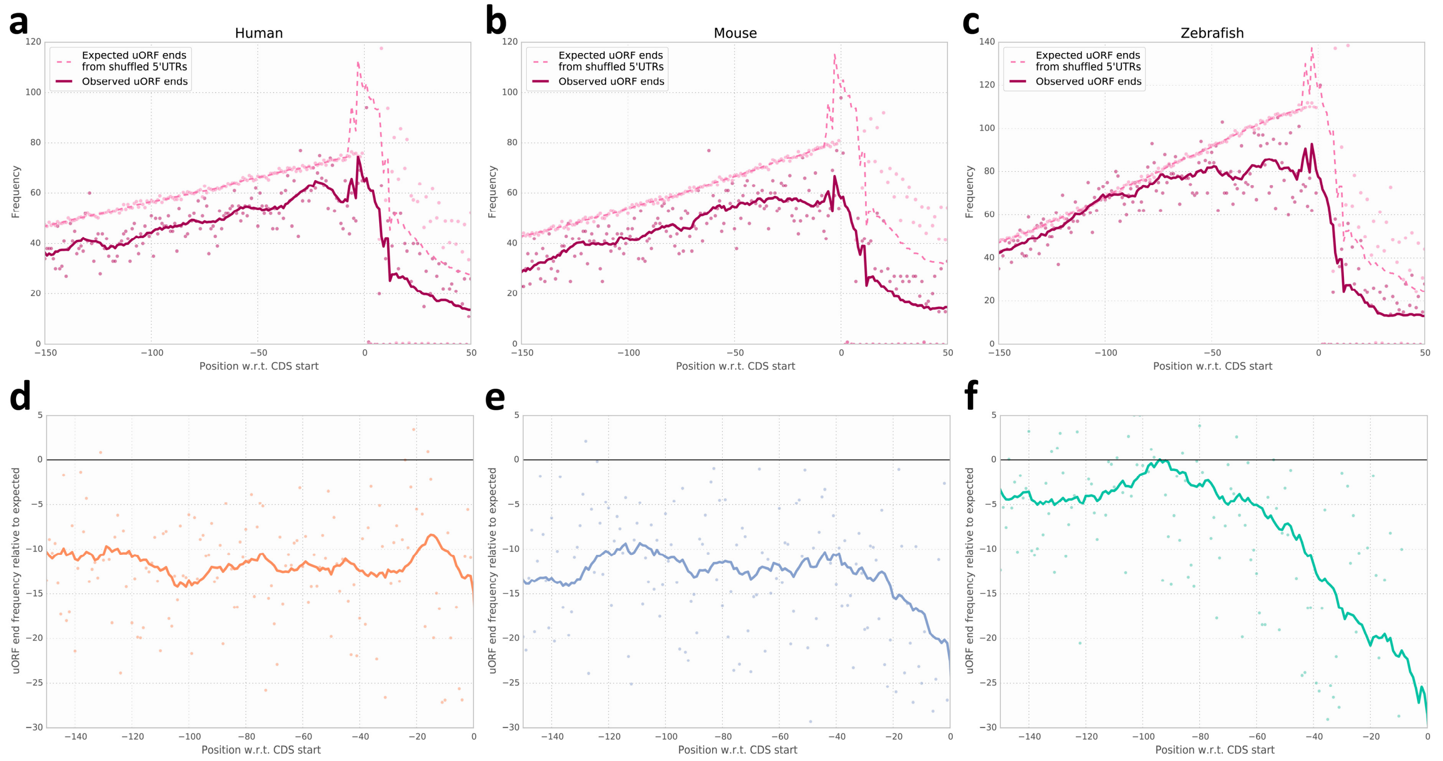

<u>**Supplementary Fig. 4:** Characterization of position-specific depletion of uORFs in 5' leaders in vertebrates</u>

a-c. Frequency of uORF ends with respect to CDS start, in observed (dark magenta) and shuffled (light magenta) 5' leaders, for all 3 vertebrates: **(a)** human, **(b)** mouse, **(c)** zebrafish. Moving averages over 15 nucleotides are plotted alongside scatter points of individual positions.

d-f. Depletion in the frequency of uORF ends (observed - expected number of uORF ends) relative to expected frequencies from shuffled 5' leaders, plotted against uORF end position with respect to CDS start, for all 3 vertebrates: **(d)** human, **(e)** mouse, **(f)** zebrafish. Moving averages over 24 nucleotides are plotted with underlying scatterplot.

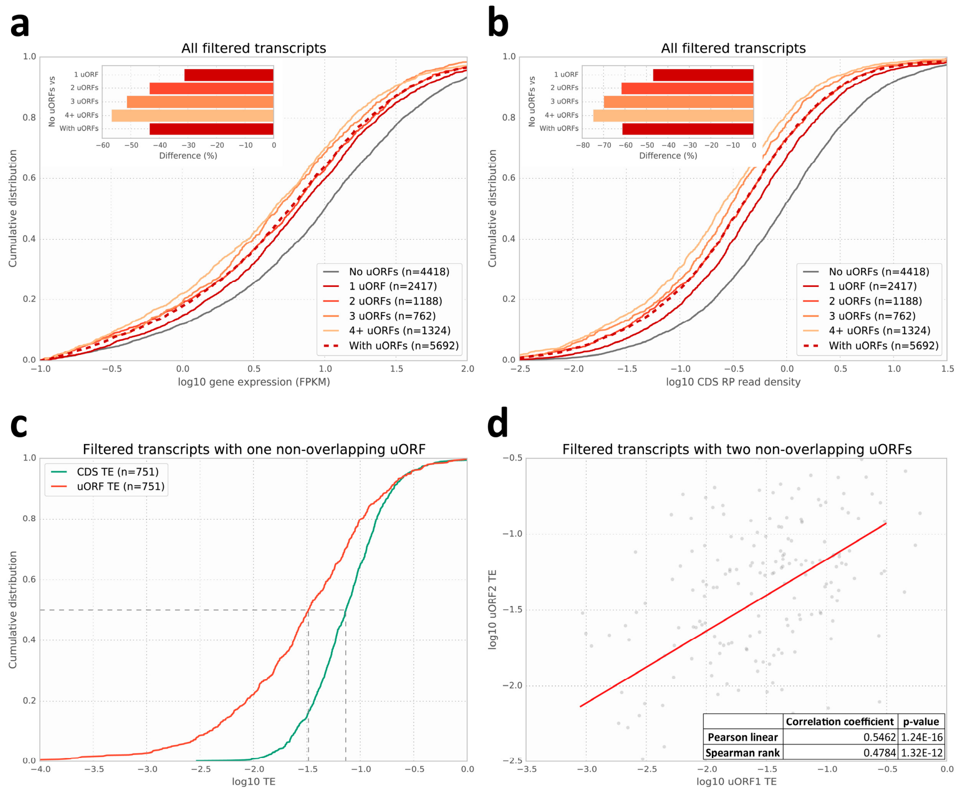

<u>**Supplementary Fig. 5:** Relationships between number of uORFs and gene translation and expression</u>

a-b. Cumulative distribution plot of transcript expression (FPKM) **(a)** and CDS translation **(b)** in transcripts grouped by their number of uORFs. The presence of uORFs is associated with reduced transcript expression and CDS translation (insets; 32-57% and 47-75% reduction with increasing number of uORFs, averaging reductions of 43% and 62% respectively).

c. Cumulative distribution plots of uORF and CDS TEs for transcripts with one non-overlapping uORF.

d. Relationship between TEs of 1st and 2nd uORF for transcripts with two non-overlapping uORFs.

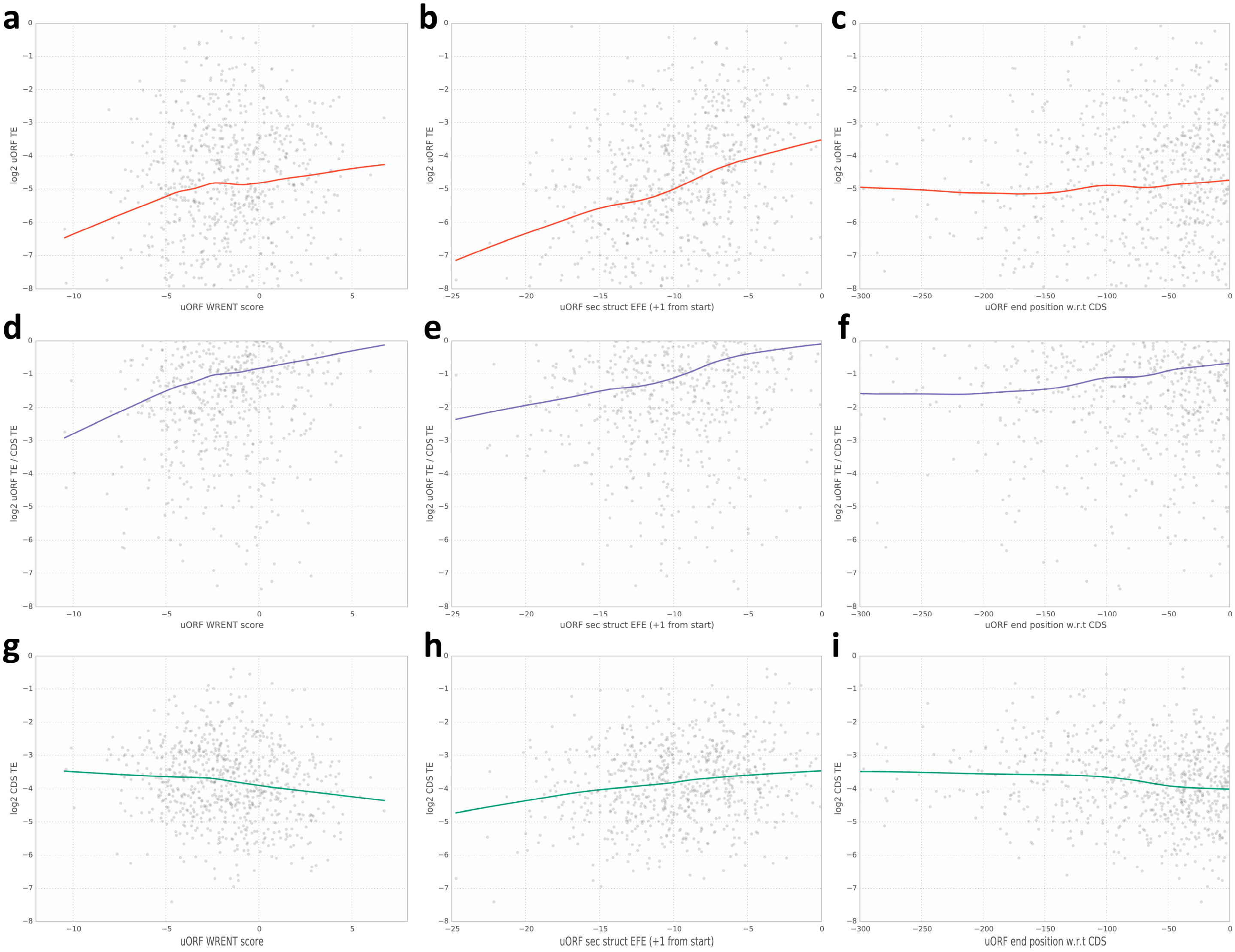

<u>**Supplementary Fig. 6:** Scatter plots and correlations for LOWESS fits relating uORF sequence features to uORF repressiveness, uORF TE and CDS TE</u>

a-i. For transcripts with one non-overlapping uORF, scatter plots of:

uORF TE / CDS TE (uORF repressiveness) **(a-c)**, CDS TE **(d-f)**, and uORF TE **(g-i)** against uORF WRENT score **(a, d, g)**, uORF secondary structure EFE at +1 position from CDS **(b, e, h)**, and position of uORF end with respect to CDS **(c, f, i)**.

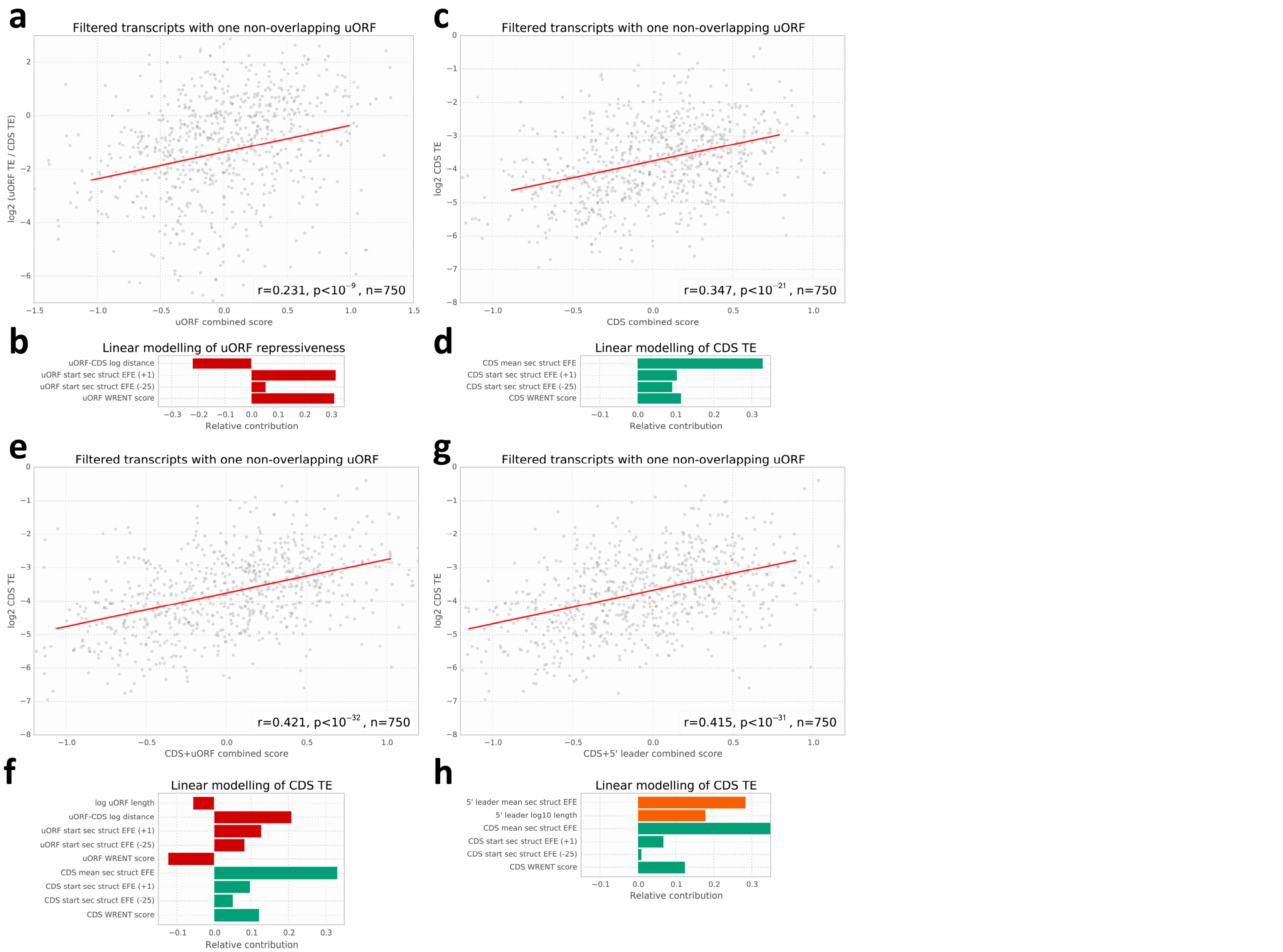

Supplementary Fig. 7: Linear modeling of uORF repressiveness and CDS TE with reduced subsets of transcript sequence features

a-b. Linear modeling of uORF TE / CDS TE with uORF sequence features only, for transcripts with one non-overlapping uORF**.** Scatter plot of uORF TE / CDS TE against a combined score that integrates uORF and CDS sequence features (a); red line indicates the ridge regression linear fit. Relative contributions of individual sequence features to the combined score are depicted in **(b)**.

c-h. Linear modeling of CDS TE with various combinations of uORF, 5' leader and CDS sequence features, for transcripts with one non-overlapping uORF: CDS sequence features only **(c, d)**, or after addition of either uORF **(e, f)**, or 5' leader **(g, h)** sequence features.

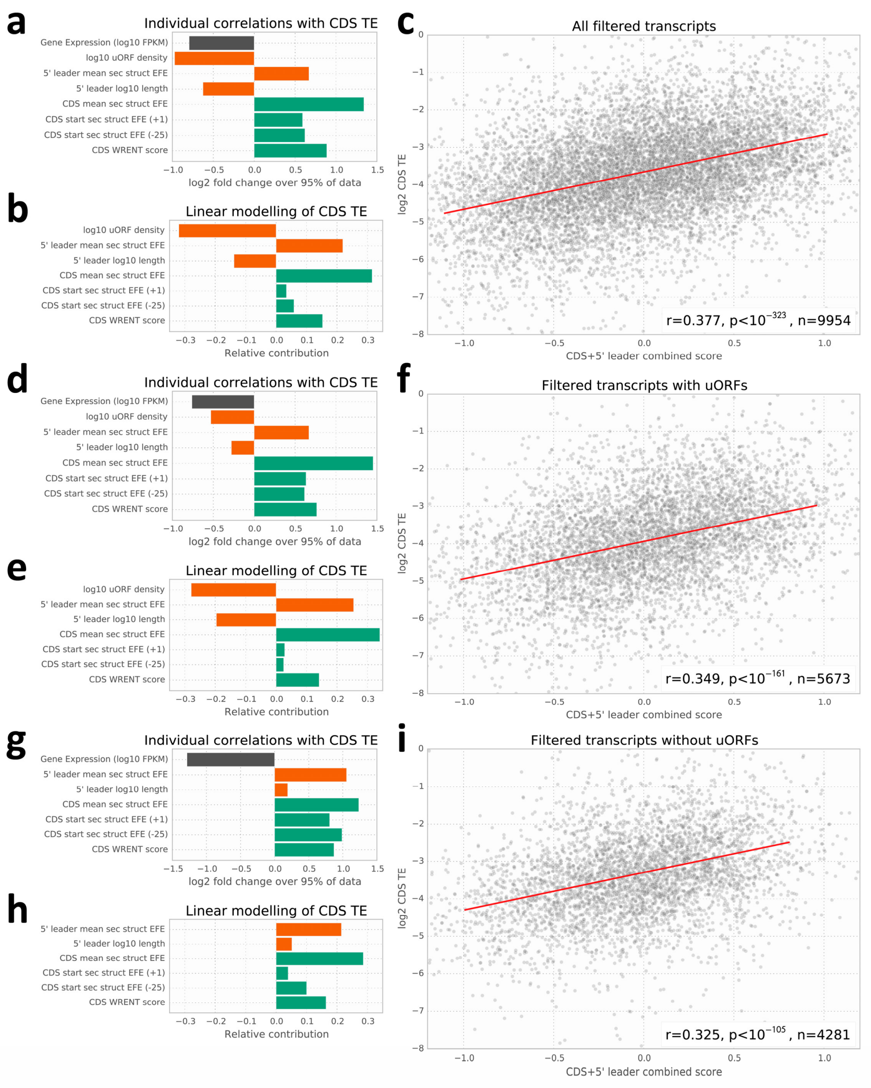

<u>**Supplementary Fig. 8:** Individual correlations of transcript sequence features and linear modeling with CDS TE in transcript subsets with and without uORFs</u>

a, d, g. Linear correlations of individual 5’ leader and CDS sequence features with CDS TE, for various transcript subsets: all filtered transcripts **(a)**, filtered transcripts with uORFs **(d)**, and filtered transcripts without uORFs **(g)**. Horizontal bars summarize the fold-change over 95% of the data.

b, c; e, f; h, i. Linear modeling of CDS TE with 5’ leader and CDS sequence features in all filtered transcripts **(b, c)**, filtered transcripts with uORFs **(e, f)**, and filtered transcripts without uORFs **(h, i)**. Figures shown are similar to those in Supplementary Fig. 7.

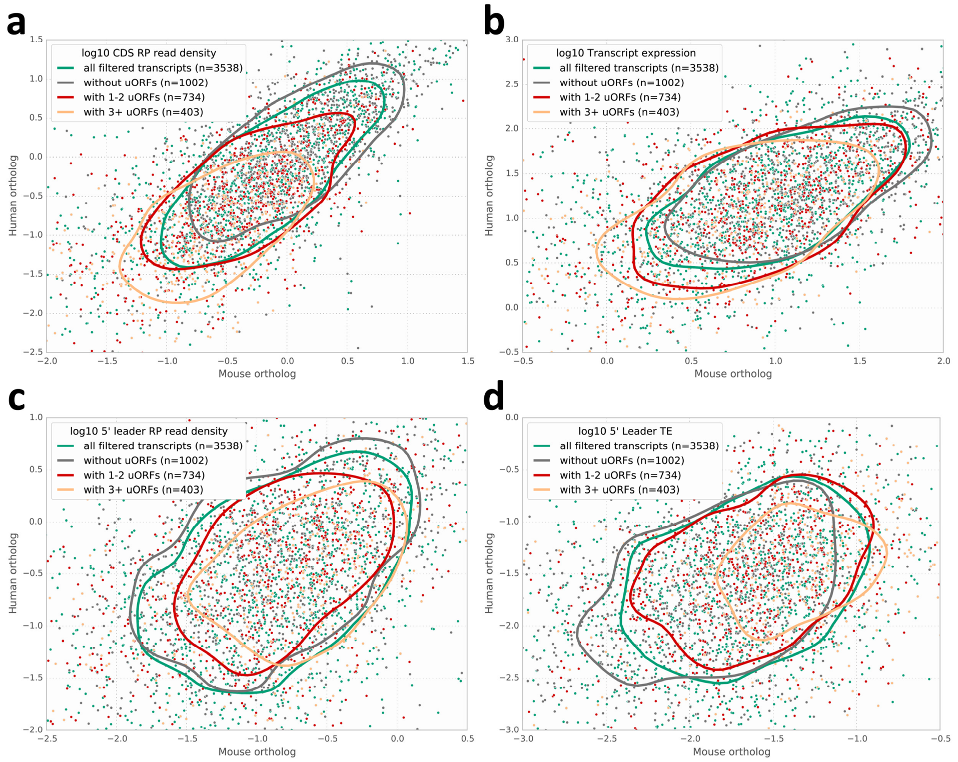

<u>**Supplementary Fig. 9:** Conservation of translation between orthologous human and mouse transcripts</u>

a-d. Scatter plots of CDS translation **(a)**, transcript expression **(b)**, 5' leader translation **(c),** and 5' leader TE **(d)**, for human and mouse orthologs. Scatter points are colored by the number of uORFs in the orthologous pairs of transcripts, while contours (20th percentile values of a bivariate Gaussian kernal density estimator) depict the distribution of each subset of points.

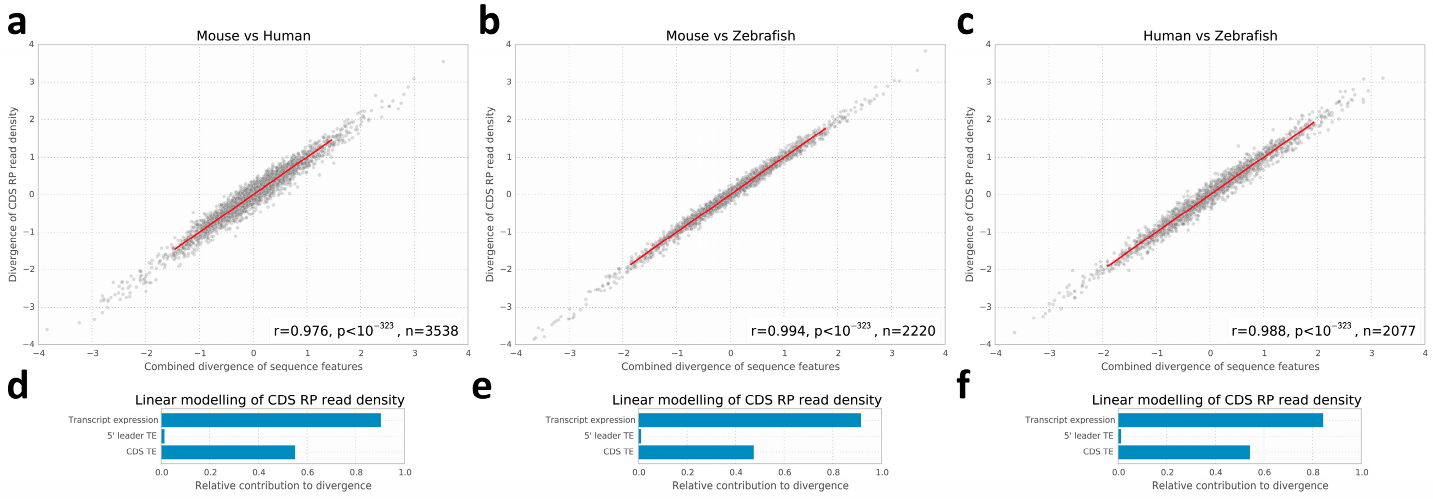

<u>**Supplementary Fig. 10:** Contribution of transcript expression and CDS TE to the divergence of total gene translation between various vertebrates</u>

Linear modeling of the divergence of total CDS translation (measured as CDS ribosome profiling read densities) with the divergences of transcript expression, 5' leader TE, and CDS TE, in pairwise comparisons of orthologous transcripts between mouse and human **(a, d)**, mouse and zebrafish **(b, e)**, and human and zebrafish **(c, f).** Figures shown are similar to those in Supplementary Fig. 7 and 8.

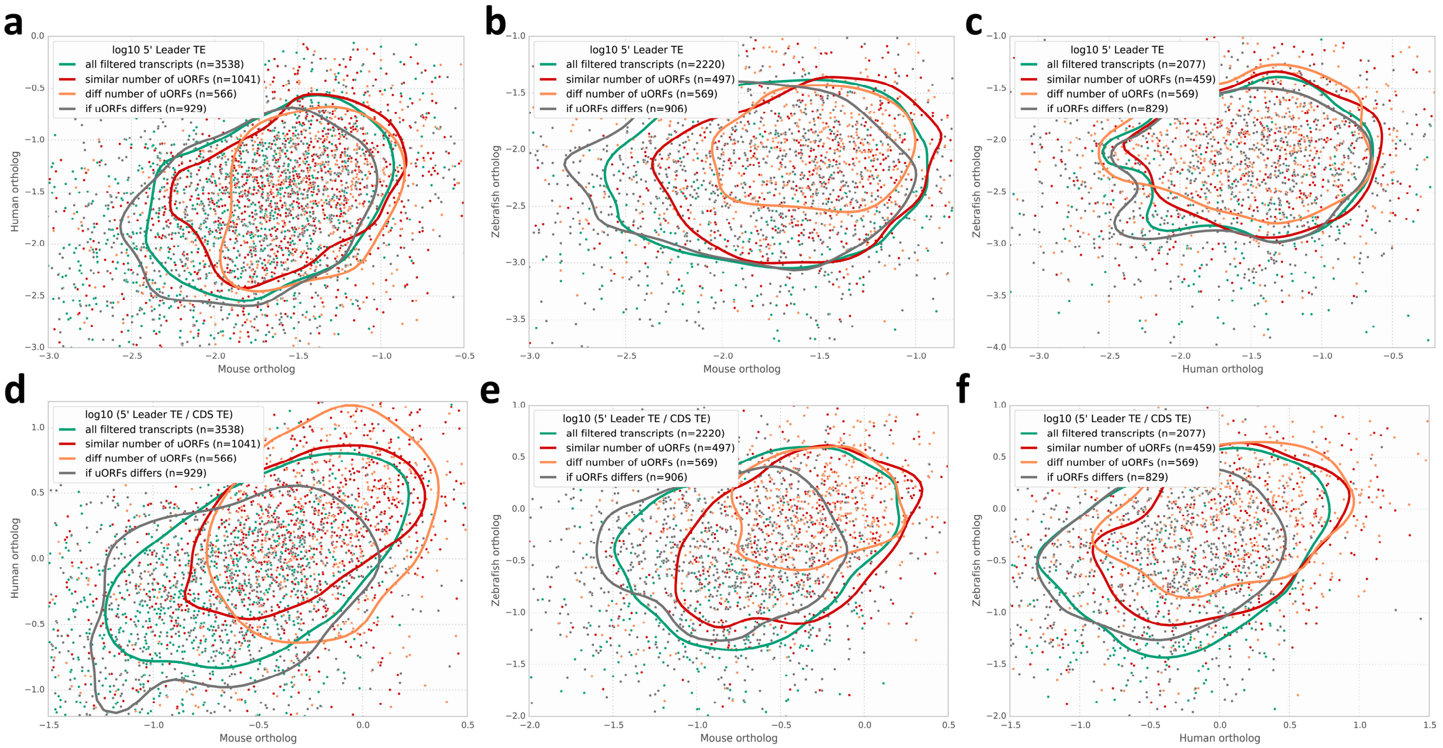

Supplementary Fig. 11: Conservation of 5’ leader TE and repressiveness between vertebrates

Conservation of translation over orthologous transcripts between mouse and human **(a, d)**, mouse and zebrafish **(b, e)**, and human and zebrafish **(c, f)**. Scatter plots of 5’ leader TE **(a-c)** and 5’ leader repressiveness **(d-f)** for orthologous transcripts are grouped and colored by whether transcripts have similar or differing numbers of uORFs (difference in number of uORFs ≥ 2), with contours (20th percentile values of a bivariate Gaussian kernal density estimator) indicating the distribution of the subsets.

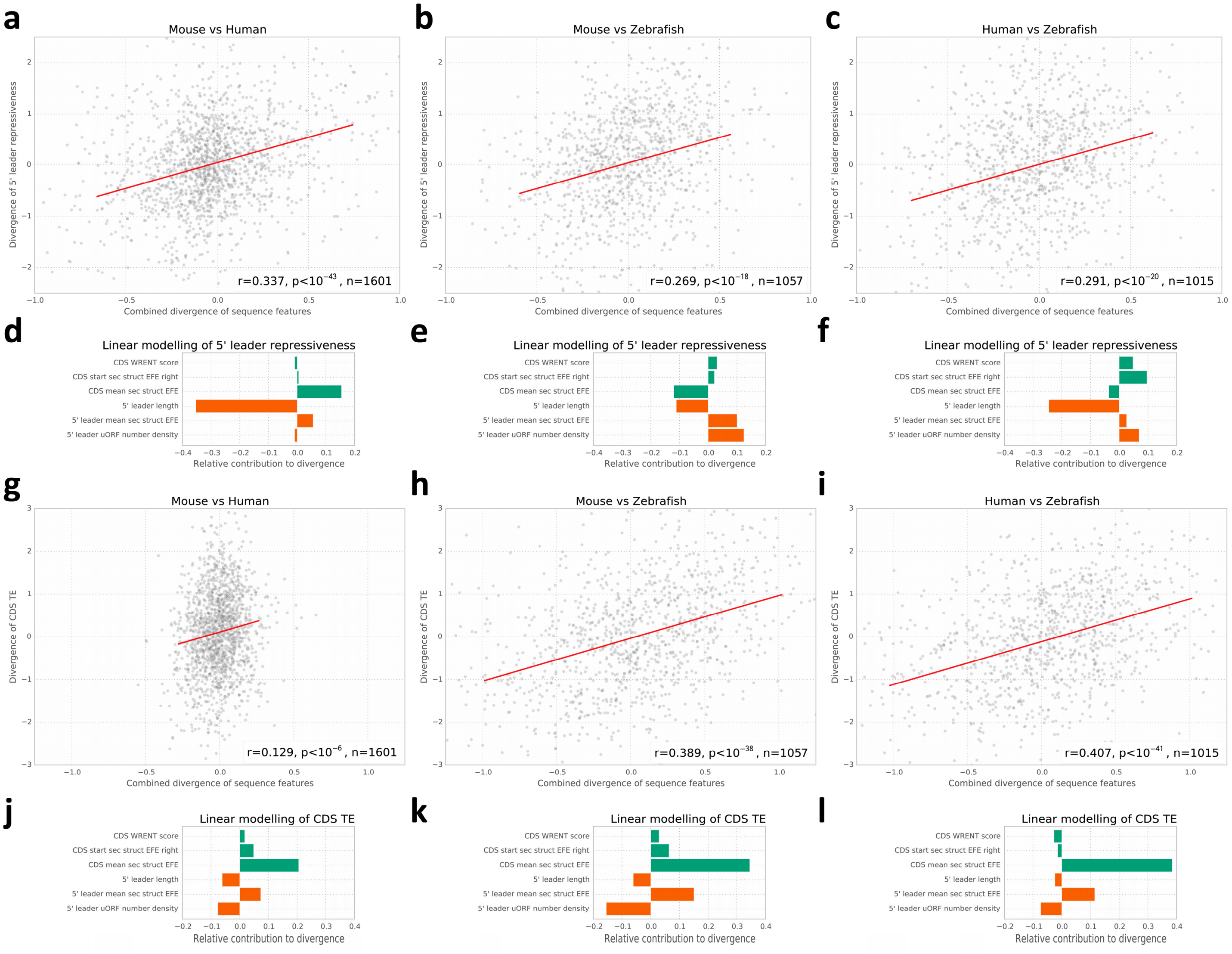

<u>Supplementary Fig. 12:</u> <u>Contribution of various transcript sequence features to the divergence of 5’ leader repressiveness and CDS TE between species</u>

Linear modeling of the divergence of 5’ leader repressiveness **(a-f)** and CDS TE **(g-l)** with the divergences of various 5’ leader and CDS sequence features, in pairwise comparisons of orthologous transcripts between mouse and human **(a, d, g, j)**, mouse and zebrafish **(b, e, h, k)**, and human and zebrafish **(c, f, I, l).** Figures shown are similar to those in Supplementary Fig. 7, 8 and 11.

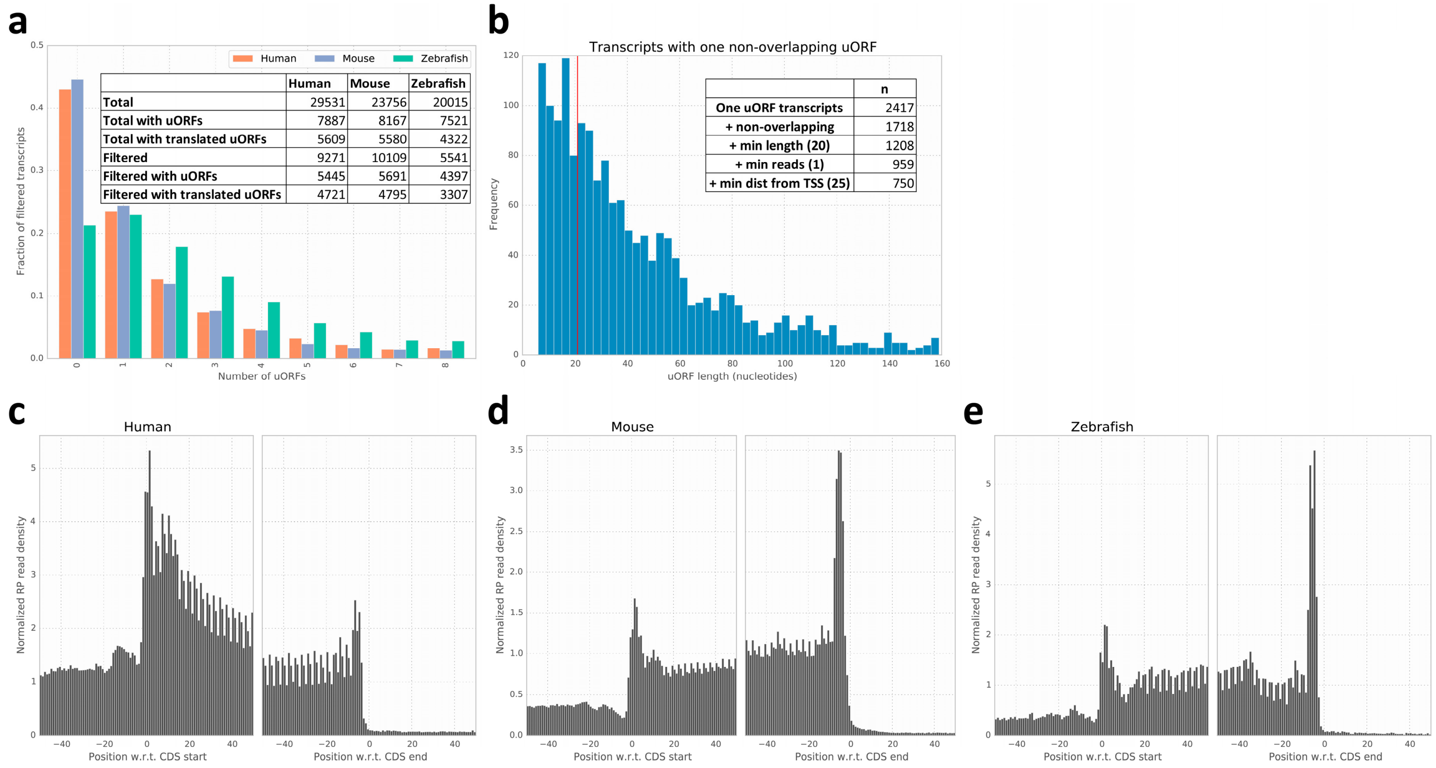

Supplementary Fig. 13: Analysis of uORF numbers and quality of ribosome profiling data

a. Histograms of number of uORFs, for human, mouse and zebrafish filtered transcripts. About half of mammalian transcripts and two-thirds of zebrafish transcripts contain uORFs. Inset shows summary of filtering for 3 vertebrates.

b. Distribution of uORF lengths, with the red line indicating cut-off of 21 nucleotides (6 amino acids + stop). Inset shows summary of filtering to obtain the subset of mouse transcripts with one non-overlapping uORF.

c-e. Metagene profiles of ribosome profiling reads around CDS starts and ends, for three ribosome profiling datasets (**c.** Human – HeLa cells; **d.** Mouse – mES cells; **e.** Zebrafish – shield stage embryos). Triplet phasing is detected, as are differing 5' and 3' biases in ribosome profiling reads around the starts and ends (caused by differing cycloheximide treatment; HeLa cells were pre-treated prior to lysis, mES cells and shield stage embryos were treated during lysis).

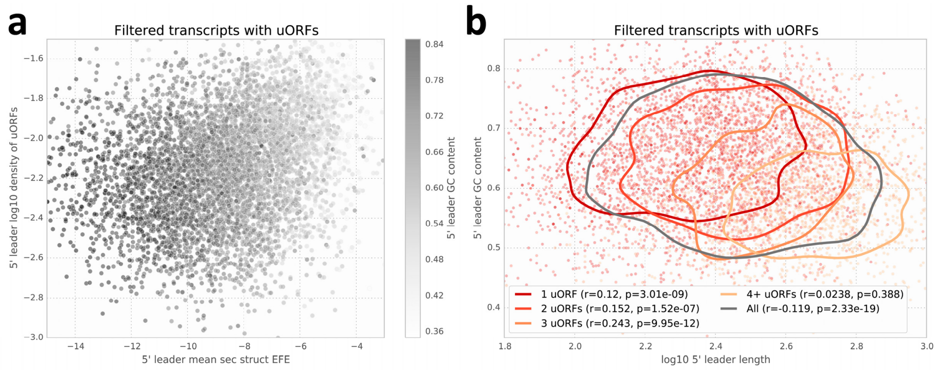

Supplementary Fig. 14: Complex relationship between 5' leader uORF density, secondary structure and GC content

a. Scatter plot of 5' leader density of uORFs against 5' leader mean secondary structure EFE. Scatter points are colored by their 5' leader GC content, which anti-correlates with mean secondary structure EFE. Significant correlation between density of uORFs and mean secondary structure EFE in 5' leaders is evident.

b. Simpson's paradox type relationship amongst 5' leader GC content, 5' leader length, and number of uORFs. While there are significant positive correlations between 5' leader GC content and length for individual subsets of the data (grouped by their number of uORFs), there is an overall negative correlation between GC content and 5' leader length.

SUPPLEMENTARY TABLES

Supplementary Table 1: Summary statistics of linear correlations with uORF WRENT score, uORF secondary structure EFE at +1, and uORF end position with respect to CDS start

	Parameter	Pearson r	p	Fold change
**uORF WRENT score**	*uORF TE / CDS TE*	0.138	0.000151	2.352
	CDS TE	-0.09225	0.01148	-1.383
	uORF TE	0.08458	0.02053	1.7
**uORF sec. struct. EFE at +1 position**	*uORF TE / CDS TE*	0.1544	2.17E-05	2.554
	CDS TE	0.1564	1.68E-05	1.714
	uORF TE	0.2402	2.67E-11	4.379
**uORF end position w.r.t. CDS**	*uORF TE / CDS TE*	0.07774	0.03328	1.53
	CDS TE	-0.1394	0.000129	-1.541
	uORF TE	-0.00129	0.9718	-1.007

Includes fold change over 95% of data (rightmost column).

Supplementary Table 2: Individual correlations of sequence features with CDS TE for various transcript sets

	**All filtered transcripts**			**Filtered transcripts with uORFs**			**Filtered transcripts without uORFs**		
	**Pearson r**	**p**	**Fold change**	**Pearson r**	**p**	**Fold change**	**Pearson r**	**p**	**Fold change**
**Gene Expression (log10 FPKM)**	-0.1349	2.82E-42	0.5764	-0.1335	4.89E-24	0.5902	-0.2152	1.92E-47	0.4102
**log10 uORF density**	-0.1967	1.02E-88	0.5096	-0.09506	6.66E-13	0.6926	N.A.	N.A.	N.A.
**5' leader mean sec struct EFE**	0.1171	9.72E-32	1.591	0.1196	1.53E-19	1.59	0.1858	1.47E-34	2.078
**5' leader log10 length**	-0.1067	5.75E-27	0.6481	-0.04852	0.000251	0.8242	0.03289	0.02879	1.142
**CDS mean sec struct EFE**	0.2386	7.77E-129	2.534	0.2634	1.10E-90	2.74	0.2259	1.15E-50	2.352
**CDS start sec struct EFE (-25)**	0.103	3.09E-25	1.507	0.1122	2.09E-17	1.552	0.1416	3.14E-21	1.749
**CDS start sec struct EFE (+1)**	0.1069	4.52E-27	1.537	0.1101	8.36E-17	1.532	0.1726	7.16E-31	1.983
**CDS WRENT score**	0.1533	3.50E-54	1.85	0.134	3.22E-24	1.699	0.154	7.24E-25	1.828

Supplementary Table 3: Summary statistics of linear modelling of CDS TE with various sets of transcript sequence features, for various transcript sets

	Parameters	Pearson r	p	Fold change	PRESS	RESS	n
**All filtered transcripts**	*CDS*	0.2756	5.91E-173	2.985	1.99E+04	1.99E+04	9,954
	CDS + 5' leader	0.3769	0	4.349	1.85E+04	1.85E+04	
**With uORFs**	*CDS*	0.2874	2.44E-108	3.055	1.10E+04	1.10E+04	5,673
	CDS + 5' leader	0.3489	3.97E-162	3.92	1.05E+04	1.05E+04	
**Without uORFs**	*CDS*	0.2948	1.38E-86	3.078	8,058	8,040	4,281
	CDS + 5' leader	0.3248	9.75E-106	3.474	7,903	7,876	

Supplementary Table 4: Summary of the correlations for whole and subsets of datasets for various measures of gene expression, subsetted by number of uORFs

	CDS TE	5' Leader TE	(5' Leader TE / CDS TE)	CDS RP read density	5' leader RP read density	Transcript expression	n
**all filtered transcripts**	0.494***	0.358***	0.515***	0.727***	0.442***	0.6***	3538
**without uORFs**	0.495***	0.423***	0.463***	0.691***	0.459***	0.591***	1002
**with 1-2 uORFs**	0.496***	0.325***	0.421***	0.665***	0.483***	0.559***	734
**with 3+ uORFs**	0.382***	0.427***	0.653***	0.682***	0.484***	0.609***	403

*: $p < 0.05$; **: $p < 0.01$; ***: $p < 0.001$

Corresponds to Fig. 4 and Supplementary Fig. 9.

Supplementary Table 5: Summary of the correlations for whole and subsets of datasets for various measures of gene expression, subsetted by similarity in the number of uORFs, Mouse vs. Human

	CDS TE	5' Leader TE	(5' Leader TE / CDS TE)	CDS RP read density	5' leader RP read density	Transcript expression	n
**all filtered transcripts**	0.494***	0.358***	0.515***	0.727***	0.442***	0.6***	3538
**similar number of uORFs**	0.457***	0.335***	0.503***	0.677***	0.496***	0.571***	1041
**diff number of uORFs**	0.516***	0.364***	0.514***	0.686***	0.365***	0.592***	566
**if uORFs differs**	0.408***	0.274***	0.363***	0.733***	0.42***	0.589***	929

*: $p < 0.05$; **: $p < 0.01$; ***: $p < 0.001$

Supplementary Table 6: Summary of the correlations for whole and subsets of datasets for various measures of gene expression, subsetted by similarity in the number of uORFs, Mouse vs. Zebrafish

	CDS TE	5' Leader TE	(5' Leader TE / CDS TE)	CDS RP read density	5' leader RP read density	Transcript expression	n
**all filtered transcripts**	0.235***	0.128***	0.286***	0.57***	0.177***	0.485***	2220
**similar number of uORFs**	0.271***	0.161***	0.322***	0.547***	0.172***	0.438***	497
**diff number of uORFs**	0.165***	0.124**	0.325***	0.482***	0.181***	0.42***	569
**if uORFs differs**	0.152***	0.0434	0.105**	0.53***	0.156***	0.488***	906

*: $p < 0.05$; **: $p < 0.01$; ***: $p < 0.001$

Supplementary Table 7: Summary of the correlations for whole and subsets of datasets for various measures of gene expression, subsetted by similarity in the number of uORFs, Zebrafish vs. Human

	CDS TE	5' Leader TE	(5' Leader TE / CDS TE)	CDS RP read density	5' leader RP read density	Transcript expression	n
**all filtered transcripts**	0.231***	0.11***	0.307***	0.526***	0.148***	0.419***	2077
**similar number of uORFs**	0.242***	0.0525	0.298***	0.524***	0.113*	0.335***	459
**diff number of uORFs**	0.277***	0.183***	0.364***	0.479***	0.152***	0.339***	569
**if uORFs differs**	0.15***	0.0948**	0.18***	0.484***	0.153***	0.446***	829

*: $p < 0.05$; **: $p < 0.01$; ***: $p < 0.001$

SUPPLEMENTARY NOTE 1

Investigating the effects of 5' leader length on CDS TE revealed a complex relationship amongst the length, secondary structure stability, and number of uORFs within the 5' leaders (Supplementary Fig. 14a). Considering that:

1. longer 5' leaders tend to have more uORFs,
2. GC-rich 5' leaders tend to have more stable secondary structures
3. AU-rich 5' leaders tend to have more ORFs per unit length (because ORFs are defined by AU-rich sequences: AUG, UAA, UAG, UGA)

it becomes apparent that individual sequence features are not independent. Indeed, there is a strong negative correlation ($r=-0.876$, $p<10^{-323}$) between GC content and mean secondary structure EFE in the 5' leader. The density of uORFs is also negatively correlated 5' leader GC content ($r=-0.346$, $p<10^{-158}$), while positively correlated with 5' leader mean secondary structure EFE ($r=0.242$, $p<10^{-75}$).

Thus, the interpretation of correlations between individual sequences features with CDS TE is complicated by the fact that constraining any one particular sequence feature (e.g. fixing the number of uORFs at one), also constrains other sequence features. These dependencies result in apparent correlations in a subset of transcripts that may not exist in the entire dataset (an example of Simpson's paradox). For example, while 5' leader length correlated positively with GC content in transcriptome subsets with only 1, 2, or 3 uORFs, 5' leader length correlated negatively with GC content when all transcripts were considered (Supplementary Fig. 14c).

The effect of these interactions is most evident when modeling the influence of 5' leader length on CDS TE: while the length of 5' leaders unexpectedly correlated with CDS TE in the subset of transcripts with only one non-overlapping uORF and in transcripts lacking uORFs (Fig. 3g and Supplementary Fig. 8g, h), the expected negative correlation was observed over the larger subset of transcripts containing any uORFs (Supplementary Fig. 8d, e), as well as in the entire set of filtered transcripts (Supplementary Fig. 8a, b).
