# Supplementary material for: Conservation of uORF repressiveness and sequence features in mouse, human and zebrafish
Source: Nat Commun. 2016 May 24;7:11663. doi: 10.1038/ncomms11663 (PMC4890304; doi:10.1038/ncomms11663)
Supplement: Supplementary Software — Conversions of iPython/Jupyter notebooks documenting all analysis for manuscript. Latest versions of iPython/Jupyer notebooks are available at https://github.com/chewgl/uORF_repressiveness_supplemental [file ncomms11663-s2.zip › 2. RNA folding profiles in windows over transcriptome.html]

2. RNA folding profiles in windows over transcriptome


# 2. RNA folding profiles in windows over transcriptome¶

RNA folding on the scale of a whole transcriptome (to obtain profiles over uORFs and CDSes) was computationally intensive, and is thus documented in a separate iPython notebook here. Each transcriptome was split into 1000-transcript segments and folded in individual cluster jobs, before being re-collated.

Secondary structure profiles were then compiled around AUGs throughout the transcript, further subdivided by how many uORFs were present in the transcript. Mean secondary structure ensemble folding energies were also determined over entire 5' leaders, uORFs and CDSes.

### Calculating transcript 5' leader, CDS and 3' UTR lengths, number/position of uORFs¶

In [ ]:

```
DATA_DIR = "./data/"
ANNOTATIONS_DIR = "./annotations/"
FOLDED_DIR = "./folded/"
TO_FOLD_DIR = "./to_fold/"

species_assembly = {"mm": "GRCm38_ens",
                    "hs": "GRCh37_ens",
                    "dr": "Zv9_ens"}
stage_assembly = {"HeLa": "GRCh37_ens",
                  "mES": "GRCm38_ens",
                  "Shield": "Zv9_ens"}
species_stage = {"mm": "mES",
                 "hs": "HeLa",
                 "dr": "Shield"}

stages = ["mES", "HeLa", "Shield"]
species = ["mm", "hs", "dr"]
```

In [ ]:

```
import re
import pandas as pd
import numpy as np

from Bio import SeqIO
from pandas import DataFrame, Series
from numpy import zeros
from ast import literal_eval
```

Lengths of 5' leaders, CDSes and 3' UTRs were calculated, along with the number of uORFs. These were stored in **\_tlengths\_uORFs.df** files for later reference.

In [ ]:

```
INDEX = "Transcript"
COLUMNS = ["Gene", "Gene_Name", "Gene_Expression_FPKM", "UTR5_length", "UTR3_length", "CDS_length",
           "num_uORFs", "uORFs_starts", "uORFs_ends"]

CONVERTERS = {i:literal_eval for i in ("ORF_starts", "ORF_ends", "RPF_csvProfile", "CDS")}
```

In [ ]:

```
for s in species:
    stage = species_stage[s]
    df_main = DataFrame(columns=COLUMNS)
    df_main.index.name = INDEX

    # FILE ITERATOR FOR .trpedf
    trpedf_file_iterator = pd.read_table(DATA_DIR + s + "/" + stage + "_canonical.trpedf",
                                         converters=CONVERTERS, chunksize=1)

    # ITERATE OVER .trpedf
    for transcript in trpedf_file_iterator:

        # Reads in data from each transcript
        RPF_csvProfile = transcript["RPF_csvProfile"][0]
        ORF_starts = transcript["ORF_starts"][0]
        ORF_ends = transcript["ORF_ends"][0]
        if type(ORF_starts) is np.int64:      # corrects for single-entry
            ORF_starts = (ORF_starts,)
            ORF_ends = (ORF_ends,)
        CDS = transcript["CDS"][0]
        ORFs = zip(ORF_starts, ORF_ends)
        uORFs = [ORF for ORF in ORFs if ORF[0] < CDS[0]]  # uORFs defined as beginning before CDS

        # Calculate and store values in main dictionary
        entry = {}
        for j in ("Gene", "Gene_Name", "Gene_Expression_FPKM"):
            entry[j] = transcript[j][0]
        entry["UTR5_length"] = CDS[0]
        entry["UTR3_length"] = len(RPF_csvProfile) - CDS[1]
        entry["CDS_length"] = CDS[1] - CDS[0]
        entry["num_uORFs"] = len(uORFs)
        entry["uORFs_starts"] = [int(uORF[0]) for uORF in uORFs]
        entry["uORFs_ends"] = [int(uORF[1]) for uORF in uORFs]

        df_main.loc[transcript["Transcript"][0]] = Series(entry)
    df_main.to_csv(DATA_DIR + s + "/" + stage + "_tlengths_uORFs.df", sep="\t")
```

## Folding RNAs in sliding windows¶

The ViennaRNA Package (Version 2.1.7) was compiled for python use (`./configure --with-python` followed by `make` and `make install`), and used to fold sliding windows of transcripts in the whole transcriptome.

FASTA files were split into 1000-entry files to be folded in parallel on a computational cluster.

In [ ]:

```
for species, assembly in species_assembly.iteritems():
    seqs = SeqIO.index(ANNOTATIONS_DIR + assembly + "_gene_canonical.fasta", "fasta")
    length = len(seqs)
    
    seqs = SeqIO.parse(ANNOTATIONS_DIR + assembly + "_gene_canonical.fasta", "fasta")
    num_files = length / 1000 + 1
    for num in xrange(num_files):
        with open(TO_FOLD_DIR + species + "_" + str(num) + ".fasta", "w+") as f:
            for i in xrange(1000):
                record = seqs.next()
                f.write(">" + record.id + "\n" + str(record.seq) + "\n")
```

The following small python script was used to fold the split fasta files in parallel.

This results in **\_NN.fasta** files (e.g. **mm\_25.fasta**) in the **./to\_fold/** directory.

In [ ]:

```
# Create fold_all.py from the following
import sys
import RNA

from Bio import SeqIO
from numpy import zeros
from ast import literal_eval

s = sys.argv[1]
WINDOW = literal_eval(sys.argv[2])
BLOCK = literal_eval(sys.argv[3])
FASTA_FILE = s + "_" + str(BLOCK) + ".fasta"
SEQS = SeqIO.index(FASTA_FILE, "fasta")

def fold_transcript(seq, WINDOW):
    energies = zeros(len(seq) - WINDOW)
    for i in range(len(seq) - WINDOW):
        energies[i] = RNA.pf_fold(seq[i:i + WINDOW])[1]
    return energies

with open("_".join([s, str(WINDOW), str(BLOCK)]) + ".df", "w+") as f:
    f.write("\t".join(("Transcript", "ss_efe_profile")) + "\n")
    for transcript in SEQS:
        seq = str(SEQS[transcript].seq)
        if len(seq) < 100: continue
        f.write(transcript + "\t" +  ",".join([str(i) for i in fold_transcript(seq, WINDOW)]) + "\n")
```

The following commands folds the split fasta files. The commands should be modified for specific cluster job management software (e.g. SLURM, LSF).

The transcript sequences were folded in various window sizes (25, 30, 35, 40).

In [ ]:

```
%%bash
SPECIES="mm"
for WINDOW in 25 30 35 40; do
for BLOCK in {0..37}; do
python fold_all.py ${SPECIES} ${WINDOW} ${BLOCK}
done
done

SPECIES="hs"
for WINDOW in 25 30 35 40; do
for BLOCK in {0..55}; do
python fold_all.py ${SPECIES} ${WINDOW} ${BLOCK}
done
done

SPECIES="dr"
for WINDOW in 25 30 35 40; do
for BLOCK in {0..31}; do
python fold_all.py ${SPECIES} ${WINDOW} ${BLOCK}
done
done
```

Folded transcripts were collated into single files. Due to their size, these files are not included in the supplemental data, but may be similarly generated from the above code. The data needed for the data analyses ipython notebooks are extracted into their own files.

In [ ]:

```
%%bash
SPECIES="mm"
for WINDOW in 25 30 35 40; do
printf 'Transcript\tss_efe_profile\n' > ./folded/${SPECIES}_${WINDOW}_ssefes.df
cat ${SPECIES}_${WINDOW}_{0..37}.df | grep -v Transcript >> ./folded/${SPECIES}_${WINDOW}_ssefes.df
done

SPECIES="hs"
for WINDOW in 25 30 35 40; do
printf 'Transcript\tss_efe_profile\n' > ./folded/${SPECIES}_${WINDOW}_ssefes.df
cat ${SPECIES}_${WINDOW}_{0..55}.df | grep -v Transcript >> ./folded/${SPECIES}_${WINDOW}_ssefes.df
done

SPECIES="dr"
for WINDOW in 25 30 35 40; do
printf 'Transcript\tss_efe_profile\n' > ./folded/${SPECIES}_${WINDOW}_ssefes.df
cat ${SPECIES}_${WINDOW}_{0..31}.df | grep -v Transcript >> ./folded/${SPECIES}_${WINDOW}_ssefes.df
done
```

## Profiles around AUGs, subdivided by position in transcript and number of uORFs¶

The following cells collates secondary structure profiles over ORF starts (for Fig 1C), subdivided by their position in the transcript (5' leader, CDS start, within the CDS, or in the 3' UTR), as well as (for uORFs) by the number of uORFs within the transcript (for Fig S2B).

In [ ]:

```
windows = (25, 30, 35, 40)

converter = {"ss_efe_profile": literal_eval}
CONVERTERS = {i:literal_eval for i in ("uORFs_starts", "uORFs_ends")}
```

In [ ]:

```
def ATG_poss(string):
    return [ATG.start() for ATG in re.finditer("ATG", string)]

def ATG_5CI3(ATG_positions, CDS):
    return "".join(['C' if ATG_pos == CDS[0] else \
                    '5' if ATG_pos < CDS[0] else \
                    'I' if CDS[0] < ATG_pos < CDS[1] else \
                    '3' for ATG_pos in ATG_positions])
```

In [ ]:

```
for s in species:
    stage = species_stage[s]
    tlengths = pd.read_table(DATA_DIR + s + "/" + stage + "_tlengths_uORFs.df",
                             sep="\t", index_col=0, converters=CONVERTERS)
    FASTA_FILE = ANNOTATIONS_DIR + stage_assembly[stage] + "_genes_canonical.fasta"
    SEQS = SeqIO.index(FASTA_FILE, "fasta")
    
    for window in windows:
        data_iterator = pd.read_table(FOLDED_DIR + s + "_" + str(window) + "_ssefes.df",
                                      sep="\t", index_col=0,
                                      converters={"ss_efe_profile": literal_eval},
                                      chunksize=100)

        ATG_5CI3_profiles = {pos: Series(np.zeros(100), index=np.arange(-50, 50)) for pos in "5CI3"}
        ATG_5CI3_count = {pos: 0 for pos in "5CI3"}
        
        uORF_profiles = {num: Series(np.zeros(100), index=np.arange(-50, 50)) for num in range(1, 5)}
        uORF_count = {num: 0 for num in range(1, 5)}
        
        for data in data_iterator:
            data = data[data.index.isin(tlengths.index)]
            data["UTR5_length"] = tlengths.UTR5_length[data.index]
            data["UTR3_length"] = tlengths.UTR3_length[data.index]
            data["CDS_length"] = tlengths.CDS_length[data.index]
            data["fold_length"] = data.ss_efe_profile.apply(len)
            data["transcript_length"] = data.UTR5_length + data.UTR3_length + data.CDS_length
            data["CDS"] = data.apply(lambda x: (x.UTR5_length, x.UTR5_length + x.CDS_length), axis=1)
            data["seq"] = Series({i:str(SEQS[i].seq) for i in data.index})
            data["ATG_positions"] = data.seq.apply(ATG_poss)
            data["ATG_5CI3"] = data.apply(lambda x: ATG_5CI3(x.ATG_positions, x.CDS), axis=1)
            data["num_uORFs"] = tlengths.num_uORFs[data.index]
            
            for _, entry in data.iterrows():
                for pos, ATG_start in zip(entry.ATG_5CI3, entry.ATG_positions):
                    if (ATG_start > 50) and (ATG_start + 50 + window < entry.transcript_length):
                        slice_ATG = Series(np.array(entry.ss_efe_profile[ATG_start - 50: ATG_start + 50]),
                                           index=np.arange(-50, 50))
                        ATG_5CI3_profiles[pos] = ATG_5CI3_profiles[pos].add(slice_ATG)
                        ATG_5CI3_count[pos] = ATG_5CI3_count[pos] + 1
                
                for num in range(1, 5):
                    if entry.num_uORFs == 0: break
                    if entry.num_uORFs <= num:
                        uORF_starts = [uORF_start for pos, uORF_start in \
                                       zip(entry.ATG_5CI3, entry.ATG_positions) if pos == "5"]
                        for uORF_start in uORF_starts:
                            if (uORF_start > 50) and (uORF_start + 50 + window < entry.transcript_length):
                                slice_uORF = Series(np.array(entry.ss_efe_profile[uORF_start - 50: uORF_start + 50]),
                                                    index=np.arange(-50, 50))
                                uORF_profiles[num] = uORF_profiles[num].add(slice_uORF)
                                uORF_count[num] = uORF_count[num] + 1
                        break
                            
        for pos in ATG_5CI3_profiles:
            ATG_5CI3_profiles[pos] = ATG_5CI3_profiles[pos].divide(ATG_5CI3_count[pos])
        for num in uORF_profiles:
            uORF_profiles[num] = uORF_profiles[num].divide(uORF_count[num])
        
        ATG_5CI3_out = DATA_DIR + s + "/" + s + "_" + str(window) + "_RNA_fold_ATG_profiles_by_pos"
        DataFrame(ATG_5CI3_profiles).to_csv(ATG_5CI3_out, sep="\t")
        uORF_profiles_out = DATA_DIR + s + "/" + s + "_" + str(window) + "_RNA_fold_uORF_profiles_by_num"
        DataFrame(uORF_profiles).to_csv(uORF_profiles_out, sep="\t")
```

### Calculate mean secondary structure EFE over 5' leader and CDS¶

The following cells calculate the mean secondary structure EFEs in various window sizes over the lengths of all 5' leaders and CDSes (for Fig S2G, also used in linear modelling).

In [ ]:

```
for s in species:
    stage = species_stage[s]
    tlengths = pd.read_table(DATA_DIR + s + "/" + stage + "_tlengths_uORFs.df",
                             sep="\t", index_col=0, converters=CONVERTERS)
    UTR5_mean_ssefe = {"UTR5_mean_ssefe_" + str(i):{} for i in [25, 30, 35, 40]}
    CDS_mean_ssefe = {"CDS_mean_ssefe_" + str(i):{} for i in [25, 30, 35, 40]}
    
    for window in windows:
        data_iterator = pd.read_table(FOLDED_DIR + s + "_" + str(window) + "_ssefes.df",
                                      sep="\t", index_col=0,
                                      converters={"ss_efe_profile": literal_eval}, chunksize=100)
    
        for data in data_iterator:
            data = data[data.index.isin(tlengths.index)]
            data["UTR5_length"] = tlengths.UTR5_length[data.index].apply(int)
            data["CDS_length"] = tlengths.CDS_length[data.index].apply(int)
            
            chunk = data.apply(lambda x: None if (x.UTR5_length <= window) or (x.CDS_length <= 0) \
                               else np.mean(x.ss_efe_profile[:x.UTR5_length - window]),
                               axis=1)
            UTR5_mean_ssefe["UTR5_mean_ssefe_" + str(window)].update(chunk.to_dict())
            
            chunk = data.apply(lambda x: None if (x.UTR5_length <= window) or (x.CDS_length <= 0) \
                               else np.mean(x.ss_efe_profile[x.UTR5_length: x.UTR5_length + x.CDS_length - window]),
                               axis=1)
            CDS_mean_ssefe["CDS_mean_ssefe_" + str(window)].update(chunk.to_dict())
            
    UTR5_mean_ssefe = DataFrame(UTR5_mean_ssefe)
    CDS_mean_ssefe = DataFrame(CDS_mean_ssefe)
    gene_names = tlengths.Gene[tlengths.index.isin(UTR5_mean_ssefe.index)]
    mean_ssefes = pd.concat([gene_names, UTR5_mean_ssefe, CDS_mean_ssefe], axis=1)
    mean_ssefes[~np.isnan(mean_ssefes.UTR5_mean_ssefe_25)].to_csv(DATA_DIR + s + "/" + s + "_mean_ssefes.df", sep="\t")
```

### Compile SS\_EFE profiles over CDS starts on a per-transcript basis¶

For Fig S2B.

In [ ]:

```
for s in species:
    stage = species_stage[s]
    tlengths = pd.read_table(DATA_DIR + s + "/" + stage + "_tlengths_uORFs.df",
                             sep="\t", index_col=0, converters=CONVERTERS)
    
    for window in windows:
        data_iterator = pd.read_table(FOLDED_DIR + s + "_" + str(window) + "_ssefes.df",
                                      sep="\t", index_col=0,
                                      converters={"ss_efe_profile": literal_eval}, chunksize=500)
        CDS_profiles = {}
        
        for data in data_iterator:
            data = data[data.index.isin(tlengths.index)]
            data["CDS_start"] = tlengths.UTR5_length[data.index].map(int)
            data["transcript_length"] = (tlengths.UTR5_length[data.index] + \
                                         tlengths.UTR3_length[data.index] + \
                                         tlengths.CDS_length[data.index]).map(int)
            
            for transcript, entry in data.iterrows():
                if (entry.CDS_start > 50) and (entry.CDS_start + 100 + window < entry.transcript_length):
                    CDS_profiles[transcript] = Series(np.array(entry.ss_efe_profile[entry.CDS_start - 50: entry.CDS_start + 100]),
                                                      index=np.arange(-50, 100))
                    
        DataFrame(CDS_profiles).to_csv(DATA_DIR + s + "/" + s + "_" + str(window) + "_RNA_fold_CDS_start_profiles", sep="\t")
```
