# Supplementary material for: Conservation of uORF repressiveness and sequence features in mouse, human and zebrafish
Source: Nat Commun. 2016 May 24;7:11663. doi: 10.1038/ncomms11663 (PMC4890304; doi:10.1038/ncomms11663)
Supplement: Supplementary Software — Conversions of iPython/Jupyter notebooks documenting all analysis for manuscript. Latest versions of iPython/Jupyer notebooks are available at https://github.com/chewgl/uORF_repressiveness_supplemental [file ncomms11663-s2.zip › 3. Data preprocessing - transcript profiles.html]

3. Data preprocessing - transcript profiles


# 3. Data preprocessing - transcript metaprofiles¶

The initial data preprocessing steps calculate relevant data and organizes them in a format that is easy for later analysis: tab- and comma-separated files compatible with the DataFrame format ("\_main.df" and "\_profile.df"). These may be easily read by either R or Python (using the Pandas package).

This data preprocessing step collates transcript metaprofile related data for downstream analyses.

These include: ribosome metaprofiles over transcript CDS starts and ends, trinucleotide bias over 5' leaders, and uORF distribution over 5' leaders.

## Dataset¶

Mouse ES cell data from Ingolia et al. 2011 as the examplar for data preprocessing and downstream analyses. The stage and assembly parameters may be changed to preprocess and analyze HeLa cell (Human - GRCh37) and Shield stage (Zebrafish - Zv9) data.

In [ ]:

```
s = "mm"
stage = "mES"
ASSEMBLY = "GRCm38_ens"

# s = "dr"
# stage = "Shield"
# ASSEMBLY = "Zv9_ens"

# s = "hs"
# stage = "HeLa"
# ASSEMBLY = "GRCh37_ens"
```

In [ ]:

```
DATA_DIR = "./data/"
ANNOTATIONS_DIR = "./annotations/"
FOLDED_DIR = "./folded/"
TO_FOLD_DIR = "./to_fold/"
```

## Imports and parameters¶

In [ ]:

```
# IMPORTS
import pandas as pd
import numpy as np

from Bio import SeqIO
from numpy import log1p, log2
from ast import literal_eval
from pandas import Series, DataFrame
from random import sample
```

In [ ]:

```
# FILENAME PARAMETERS
TRPEDF_FILE = DATA_DIR + s + "/" + stage + "_canonical.trpedf"
CONVERTERS = {i:literal_eval for i in ("ORF_starts", "ORF_ends", "RPF_csvProfile", "CDS")}

FASTA_FILE = ANNOTATIONS_DIR + ASSEMBLY + "_genes_canonical.fasta"
SEQS = SeqIO.index(FASTA_FILE, "fasta")
```

In [ ]:

```
# OTHER PARAMETERS
UTR5_LENGTH_MIN = 12
FPKM_MIN = 0.1

CDS_LENGTH_MIN = 120
UTR_LENGTH_MIN = 60
ORF_END_TRIM = 10
```

## Trinucleotide biases and ribosome profiling metaprofiles¶

DataFrames for storing nucleotide and tri-nucleotide frequency (for calculation of trinucleotide biases), as well as DataFrames for ribosome profiling transcript metaprofile around CDSes are initialized here.

In [ ]:

```
trints = [nt1+nt2+nt3 for nt1 in "ATCG" for nt2 in "ATCG" for nt3 in "ATCG"]
nt_freq = DataFrame(index=[nt for nt in 'ATCG'], columns=range(-200, 0)).fillna(0.)
trint_freq = DataFrame(index=trints, columns=range(-200, -2)).fillna(0.)

dict_rpf_profile = {"CDS_norm_start_reads": Series(index=np.arange(-UTR_LENGTH_MIN, CDS_LENGTH_MIN)).fillna(0.),
                    "CDS_norm_end_reads"  : Series(index=np.arange(-CDS_LENGTH_MIN, UTR_LENGTH_MIN)).fillna(0.)}
```

TRPEDF file is read line-by-line, filtering for transcripts expressed above a minimum (defined in parameters).

First part reads in data, determines which ORFs are uORFs.

Second part processes data for trinucleotide bias calculations over 5' leaders.

Third part processes data for ribosome profiling metaprofile.

In [ ]:

```
trpedf_file_iterator = pd.read_table(TRPEDF_FILE, converters=CONVERTERS, chunksize=1)

for transcript in trpedf_file_iterator:
    
    # Read in data, determine uORFs
    expression = transcript["Gene_Expression_FPKM"][0]
    if expression < FPKM_MIN: continue                 # filter for FPKM minimum
    
    seq = SEQS[transcript["Transcript"][0]].seq        # get transcript sequence
    RPF_csvProfile = transcript["RPF_csvProfile"][0]
    CDS = transcript["CDS"][0]

    ORF_starts = transcript["ORF_starts"][0]           
    ORF_ends = transcript["ORF_ends"][0]
    if type(ORF_starts) is np.int64:                   # corrects for single-entry
        ORF_starts = (ORF_starts,)
        ORF_ends = (ORF_ends,)
    ORFs = zip(ORF_starts, ORF_ends)                   # zips starts and stops into ORF
    uORFs = [ORF for ORF in ORFs if (ORF[0] < CDS[0])] # uORFs defined as beginning before CDS

    # Tri-nucleotide bias
    leader_seq = str(seq[:CDS[0]][-200:])
    for pos, nt in zip(range(-len(leader_seq), 0), leader_seq):
        if nt not in "ATCG": continue
        nt_freq[pos][nt] += 1
        
    for pos, trint in zip(range(-len(leader_seq), -2),
                          [leader_seq[i:i+3] for i in range(len(leader_seq)-2)]):
        if trint not in trints: continue
        trint_freq[pos][trint] += 1
    
    
    # Ribosome profiling metaprofile over CDS start and ends
    if ((CDS[1] - CDS[0]) > CDS_LENGTH_MIN + ORF_END_TRIM
        and CDS[0] >= UTR_LENGTH_MIN and len(RPF_csvProfile) - CDS[1] >= UTR_LENGTH_MIN
        and sum(RPF_csvProfile[CDS[0]: CDS[0] + CDS_LENGTH_MIN] \
                + RPF_csvProfile[CDS[1] - CDS_LENGTH_MIN: CDS[1]]) > 100):
        
        norm = np.mean(RPF_csvProfile[CDS[0]: CDS[0] + CDS_LENGTH_MIN] + \
                       RPF_csvProfile[CDS[1] - CDS_LENGTH_MIN: CDS[1]])
        
        start_coord = (CDS[0] - UTR_LENGTH_MIN, CDS[0] + CDS_LENGTH_MIN)
        end_coord = (CDS[1] - CDS_LENGTH_MIN, CDS[1] + UTR_LENGTH_MIN)
        norm_start_reads = np.array(RPF_csvProfile[start_coord[0]: start_coord[1]]) / norm
        norm_end_reads = np.array(RPF_csvProfile[end_coord[0]: end_coord[1]]) / norm

        dict_rpf_profile["CDS_norm_start_reads"] += log1p(norm_start_reads)
        dict_rpf_profile["CDS_norm_end_reads"] += log1p(norm_end_reads)
```

Nucleotide and trinucleotide frequency data are outputted to .df files.

Ribosome profiling metaprofiles are normalized by CDS median read frequencies, also outputted to .df files.

In [ ]:

```
trint_freq.to_csv(DATA_DIR + s + "/" + stage + "_trint_freq.df", sep="\t")
nt_freq.to_csv(DATA_DIR + s + "/" + stage + "_nt_freq.df", sep="\t")

df_profile = DataFrame(dict_rpf_profile)
df_profile /= df_profile.CDS_norm_end_reads.median()
df_profile.to_csv(DATA_DIR + s + "/" + stage + "_profile.df", sep="\t")
```

## uORF end distribution around CDS¶

The following code determines the frequency of uORF ends observed in transcriptomes and compares it against a background distribution where the 5' leaders are shuffled and reattached to their cognate transcripts.

Functions are needed for shuffled-sequence analyses: redefining ORFs and shuffling sequences.

In [ ]:

```
def def_ORFs(seq):
    ORFs = []
    seq_len = len(seq)
    for frame in xrange(3):
        trans = str(seq[frame:].translate(11))
        trans_len = len(trans)
        aa_start, aa_end = [0 for i in xrange(2)]
        while aa_start < trans_len:
            aa_start = trans.find("M", aa_start)
            if aa_start == -1:
                break
            aa_end = trans.find("*", aa_start)
            ORF_start = frame + aa_start * 3
            ORF_end = frame + aa_end * 3 + 3
            if aa_end == -1:
                ORF_end = seq_len
                aa_end = trans_len
            ORFs.append((ORF_start, ORF_end))
            aa_start = aa_start + 1
        ORFs.sort()
    return ORFs

def shuffle(seq):
    return "".join(sample(seq, len(seq)))
```

Creating Series containers for frequency of uORF ends observed, vs expected (from shuffled 5' leaders).

In [ ]:

```
uORF_ends_obs = Series(index=np.arange(-200, 101)).fillna(0.)
uORF_ends_exp = Series(index=np.arange(-200, 101)).fillna(0.)

LOOPS = 100
```

.trpedf file is read, uORF end positions are noted, and 5' leader is shuffled multiple times, with uORF end positions noted. Frequencies of uORF ends around the 5' leader are outputted to .df files.

In [ ]:

```
trpedf_file_iterator = pd.read_table(TRPEDF_FILE, converters=CONVERTERS, chunksize=1)

for transcript in trpedf_file_iterator:
    expression = transcript["Gene_Expression_FPKM"][0]
    if expression < FPKM_MIN: continue
    seq = SEQS[transcript["Transcript"][0]].seq
    CDS = transcript["CDS"][0]
    if CDS[0] <= UTR5_LENGTH_MIN: continue
          
    ORF_positions = def_ORFs(seq[:CDS[0]+100])
    uORF_ends = list(set([ORF[1] - CDS[0] for ORF in ORF_positions \
                          if (ORF[0] < CDS[0] and ORF[1] - CDS[0] >= -200)]))
    for i in uORF_ends: uORF_ends_obs[i] += 1

    for loop in xrange(LOOPS):
        shuffled_UTR5 = shuffle(seq[:CDS[0]])
        ORF_positions = def_ORFs(shuffled_UTR5 + seq[CDS[0]:CDS[0] + 100])
        uORF_ends = list(set([ORF[1] - CDS[0] for ORF in ORF_positions \
                              if (ORF[0] < CDS[0] and ORF[1] - CDS[0] >= -200)]))
        for i in uORF_ends: uORF_ends_exp[i] += 1
```

In [ ]:

```
DataFrame({"uORF_ends_obs": uORF_ends_obs,
           "uORF_ends_exp": uORF_ends_exp}).to_csv(DATA_DIR + s + "/" + stage + "_uORF_ends.df", sep="\t")
```
