# Supplementary material for: Conservation of uORF repressiveness and sequence features in mouse, human and zebrafish
Source: Nat Commun. 2016 May 24;7:11663. doi: 10.1038/ncomms11663 (PMC4890304; doi:10.1038/ncomms11663)
Supplement: Supplementary Software — Conversions of iPython/Jupyter notebooks documenting all analysis for manuscript. Latest versions of iPython/Jupyer notebooks are available at https://github.com/chewgl/uORF_repressiveness_supplemental [file ncomms11663-s2.zip › 4. Data preprocessing - ORF and transcript characteristics.html]

4. Data preprocessing - ORF and transcript characteristics


# 4. Data preprocessing - ORF and transcript characteristics¶

The initial data preprocessing steps calculate relevant data and organizes them in a format that is easy for later analysis: tab- and comma-separated files compatible with the DataFrame format ("\_main.df" and "\_profile.df"). These may be easily read by either R or Python (using the Pandas package).

This data preprocessing step collates ORF characteristics (sequence features, RNA-seq and ribosome profiling data) for downstream analyses.

ORF parameters are fully described later, but include calculations for initiation context Weighted Relative ENTropy (WRENT) score, seconday structure, length, number of reads and position within transcript.

Before ORF parameters can be calculated, the position specific scoring matrix for determining WRENT scores must first be constructed.

## Dataset¶

Both the WRENT scoring matrix computation and the collation of ORF characteristics take substantial computational time (a few hours) on a desktop computer / laptop. Copies of this notebook may be concurrently opened to run these analyses on multiple datasets (by commenting / uncommenting the cell below), especially for computers with multi-core/multi-thread capabilities.

In [ ]:

```
species = "mm"
stage = "mES"
ASSEMBLY = "GRCm38_ens"

# species = "dr"
# stage = "Shield"
# ASSEMBLY = "Zv9_ens"

# species = "hs"
# stage = "HeLa"
# ASSEMBLY = "GRCh37_ens"
```

## Imports, File Locations and Parameters¶

In [ ]:

```
# IMPORTS
import RNA
import pandas as pd
import numpy as np
import matplotlib.pyplot as plt
import corebio
import weblogolib


from Bio import SeqIO
from numpy import log1p, log2
from ast import literal_eval
from pandas import Series, DataFrame

%matplotlib inline
```

'\_canonical.trpedf' means the .trpedf file was generated based on a transcriptome that used the longest transcript with longest coding sequence (historically the "canonical" transcript in transcript annotations).

Because the ORF\_starts, ORF\_ends, RPF\_csvProfile and CDS columns in the .trpedf files are comma-separated values, **literal\_eval** is used to parse them.

Corresponding fasta file is indexed using Biopython's SeqIO module.

In [ ]:

```
# FILENAME PARAMETERS
DATA_DIR = "./data/" + species + "/"
ANNOTATIONS_DIR = "./annotations/"
TRPE_FILE = DATA_DIR + stage + "_canonical.trpedf"
CONVERTERS = {i:literal_eval for i in ("ORF_starts", "ORF_ends", "RPF_csvProfile", "CDS")}

FASTA_FILE = ANNOTATIONS_DIR + ASSEMBLY + "_genes_canonical.fasta"
SEQS = SeqIO.index(FASTA_FILE, "fasta")
```

More parameters defined. Transcripts were required to have a minimum 5' UTR and RNA-seq expression value (in FPKM) to be used in determining the weighted relative entropy (WRENT) scoring matrix.

The size/position of the initiation context used for WRENT scoring and secondary structure prediction are defined around the start codon and start position respectively.

When reads over ORFs are considered, a number of positions upstream of the stop are discarded, as determined from biases detected in metagene profiles.

For plotting ribosome profiling reads around the start and stop codon of a metagene (for quality control purposes), minimum CDS and UTR lengths are required.

In [ ]:

```
# OTHER PARAMETERS
UTR5_LENGTH_MIN = 25
FPKM_MIN = 1

WRENT_CONTEXT = (10, 10) # BEFORE START CODON, AFTER START CODON
SS_CONTEXT_L = (25, 10)     # BEFORE ORF START, AFTER ORF START
SS_CONTEXT_R = (-1, 36)

ORF_END_TRIM = 10 # NUMBER OF POSITIONS BEFORE uORF END TO
                  # DISCARD READS DUE TO ARTIFACTUAL EXPERIMENTAL
                  # ACCUMULATION OF RPF READS AT uORF END.

uORF_LENGTH_MIN = 21
uORF_FROM_TSS_MIN = 25

NT_COLOR = ('#00d700', '#df1f00', '#0226cc', '#ffb700')
```

Some helper functions to retrieve sequences of initiation contexts for determining weighted Kozak scores and RNA secondary structure, as well as calculating translational efficiency (TE) of an ORF and scoring the weighted Kozak score of a sequence.

In [ ]:

```
def wrent_context_seq(seq, ORF, WRENT_CONTEXT):
    '''Takes transcript sequence and ORF (in transcript coordinates),
    along with weighted Kozak context size as inputs,
    returns sequence'''
    left_flank, right_flank = WRENT_CONTEXT
    seq_to_return = str(seq[ORF[0] - left_flank:ORF[0]] + seq[ORF[0] + 3: ORF[0] + right_flank + 3])
    if len(seq_to_return) == left_flank + right_flank:
        return seq_to_return
    else: return "A"

def ss_context_seq(seq, ORF, SS_CONTEXT):
    '''Takes transcript sequence and ORF (in transcript coordinates),
    along with secondary structure context size as inputs,
    returns sequence'''
    left_flank, right_flank = SS_CONTEXT
    seq_to_return = str(seq[ORF[0] - left_flank : ORF[0] + right_flank])
    if len(seq_to_return) == left_flank + right_flank:
        return seq_to_return
    else: return "A"

def TE(RPF_csvProfile, ORF, expression, ORF_END_TRIM):
    '''Normalizes the density of ribosome profiling reads over ORF
    by the expression of the transcript'''
    trimmed_ORF_RPF_reads = sum(RPF_csvProfile[ORF[0]:ORF[1] - ORF_END_TRIM])
    trimmed_ORF_length = max(((ORF[1] - ORF[0]) - ORF_END_TRIM), 1)
    return float(trimmed_ORF_RPF_reads) / trimmed_ORF_length / expression

def wrent_score_seq(en_score, seq_to_score):
    '''Scores a sequence based on weighted entropy score matrix'''
    return sum([en_score[pos][nt] for pos, nt in enumerate(seq_to_score)])
```

In [ ]:

```
def UTR5_uORF_segments(CDS, uORFs):
    is_uORF = [False for i in xrange(int(CDS[0]))]
    for uORF in uORFs:
        for pos in xrange(uORF[0], min(uORF[1], CDS[0])):
            is_uORF[pos] = True
    return is_uORF
```

## Calculating weighted relative entropy (WRENT) scoring matrix¶

Using translational efficiencies as weights, we determine the sequence motif for efficient translation initiation based on information content, with the nucleotide frequencies at the initiation context as background.

To do this, we read in the frequencies (weighted and unweighted) of nucleotides at each position independently. Position-specific scoring matrices (PSSMs) are initiated as a pandas DataFrames.

A file iterator is created to read the .trpedf file line-by-line, and a counter is initiated to count the number of transcripts.

In [ ]:

```
# INITIALIZE PSSMS FOR RELATIVE ENTROPY SCORING MATRICES
en_pssm = DataFrame(index=[nt for nt in 'ATCGN'], columns=range(sum(WRENT_CONTEXT)))
en_pssm.fillna(0., inplace=True)
en_pssm_unweighted = DataFrame(index=[nt for nt in 'ATCGN'], columns=range(sum(WRENT_CONTEXT)))
en_pssm_unweighted.fillna(0., inplace=True)

uORF_en_pssm = DataFrame(index=[nt for nt in 'ATCGN'], columns=range(sum(WRENT_CONTEXT)))
uORF_en_pssm.fillna(0., inplace=True)
uORF_en_pssm_unweighted = DataFrame(index=[nt for nt in 'ATCGN'], columns=range(sum(WRENT_CONTEXT)))
uORF_en_pssm_unweighted.fillna(0., inplace=True)
```

In [ ]:

```
# INITIALIZE FILE ITERATOR, LIST OF TRANSCRIPTS
trpedf_file_iterator = pd.read_table(TRPE_FILE, converters=CONVERTERS, chunksize=1)
transcript_list = []
```

The .trpedf file is read line-by-line. ORFs are merged, and uORFs are defined as beginning before the CDS.

If the transcript passes the filter parameters defined earlier (minimum 5' UTR length and RNA-seq expression), the initiation context sequence is added to the PSSMs, either unweighted (i.e. weight of 1) weighted by the transcript CDS's translational efficiency.

In [ ]:

```
# ITERATES OVER TRANSCRIPTS IN .trpedf FILE

CDS_motif_count = 0
uORF_motif_count = 0

for transcript in trpedf_file_iterator:
    transcript_list.append(transcript["Transcript"][0])
    
    seq = SEQS[transcript["Transcript"][0]].seq        # get transcript sequence
    
    expression = transcript["Gene_Expression_FPKM"][0]
    RPF_csvProfile = transcript["RPF_csvProfile"][0]
    CDS = transcript["CDS"][0]

    ORF_starts = transcript["ORF_starts"][0]           
    ORF_ends = transcript["ORF_ends"][0]
    if type(ORF_starts) is np.int64:                   # corrects for single-entry
        ORF_starts = (ORF_starts,)
        ORF_ends = (ORF_ends,)
    ORFs = zip(ORF_starts, ORF_ends)                   # zips starts and stops into ORF
    uORFs = [ORF for ORF in ORFs if (ORF[0] < CDS[0])] # uORFs defined as beginning before CDS

    if CDS[0] >= UTR5_LENGTH_MIN and expression >= FPKM_MIN:
        if len(uORFs) == 0:                            # filter for transcripts with
                                                       # minimum 5' UTR length and expression
            weight = log1p(TE(RPF_csvProfile, CDS, expression, ORF_END_TRIM))
            context_seq = wrent_context_seq(seq, CDS, WRENT_CONTEXT)
            for pos1, nt1 in enumerate(context_seq):
                en_pssm[pos1][nt1] += weight
                en_pssm_unweighted[pos1][nt1] += 1
            CDS_motif_count += 1
        
        elif (len(uORFs) == 1)\
        and (uORFs[0][1] < CDS[0])\
        and (uORFs[0][1] - uORFs[0][0] > uORF_LENGTH_MIN)\
        and (uORFs[0][0] > uORF_FROM_TSS_MIN):             # 1 uORF, non-overlapping, min uORF length, min distance from TSS
            weight = log1p(TE(RPF_csvProfile, uORFs[0], expression, ORF_END_TRIM))
            context_seq = wrent_context_seq(seq, uORFs[0], WRENT_CONTEXT)
            for pos1, nt1 in enumerate(context_seq):
                uORF_en_pssm[pos1][nt1] += weight
                uORF_en_pssm_unweighted[pos1][nt1] += 1
            uORF_motif_count += 1
        
transcript_count = len(transcript_list)
print "%g transcripts used for CDS WRENT motif" % CDS_motif_count
print "%g transcripts used for uORF WRENT motif" % uORF_motif_count
```

We first determine relative entropy: unweighted, then weighted for TE.

'N's are ignored.

In [ ]:

```
# IGNORE N
en_pssm_unweighted.drop('N', inplace=True)
en_pssm.drop('N', inplace=True)
uORF_en_pssm_unweighted.drop('N', inplace=True)
uORF_en_pssm.drop('N', inplace=True)
```

PSSMs are stored for later.

In [ ]:

```
en_pssm_unweighted.to_csv(DATA_DIR + stage + "_pssm_unweighted.df", sep="\t")
en_pssm.to_csv(DATA_DIR + stage + "_pssm.df", sep="\t")
```

In [ ]:

```
uORF_en_pssm_unweighted.to_csv(DATA_DIR + stage + "_pssm_uORF_unweighted.df", sep="\t")
uORF_en_pssm.to_csv(DATA_DIR + stage + "_pssm_uORF.df", sep="\t")
```

For calculating both unweighted and unweighted relative entropies, the background nucleotide distribution is from the total nucleotide frequency from the unweighted PSSM.

In [ ]:

```
en_pssm = DataFrame.from_csv(DATA_DIR + stage + "_pssm.df", sep="\t")
en_pssm_unweighted = DataFrame.from_csv(DATA_DIR + stage + "_pssm_unweighted.df", sep="\t")
uORF_en_pssm = DataFrame.from_csv(DATA_DIR + stage + "_pssm_uORF.df", sep="\t")
uORF_en_pssm_unweighted = DataFrame.from_csv(DATA_DIR + stage + "_pssm_uORF_unweighted.df", sep="\t")
```

In [ ]:

```
en_unweighted_nt_freq = en_pssm_unweighted.mean(axis='columns')  # background model is the unweighted PSSM's total nucleotide freq
en_unweighted_nt_prob = en_unweighted_nt_freq / en_unweighted_nt_freq.sum(axis='rows')

en_pssm_unweighted_prob = en_pssm_unweighted / en_pssm_unweighted.sum(axis="rows")
en_unweighted_score = en_pssm_unweighted_prob.divide(en_unweighted_nt_prob, axis='rows').apply(log2)

en_pssm_prob = en_pssm / en_pssm.sum(axis='rows')             # normalized nucleotide probabilities
en_score = en_pssm_prob.divide(en_unweighted_nt_prob, axis='rows').apply(log2)
```

Stores the scoring matrices (weighted and unweighted) for later.

In [ ]:

```
en_score.to_csv(DATA_DIR + stage + "_en_score.df", sep="\t")
en_unweighted_score.to_csv(DATA_DIR + stage + "_en_unweighted_score.df", sep="\t")
```

In [ ]:

```
en_score = DataFrame.from_csv(DATA_DIR + stage + "_en_score.df", sep="\t")
en_unweighted_score = DataFrame.from_csv(DATA_DIR + stage + "_en_unweighted_score.df", sep="\t")

en_score.columns = [int(i) for i in en_score.columns]
en_unweighted_score.columns = [int(i) for i in en_unweighted_score.columns]
```

**en\_score** is subsequently used to score all ORFs.

## Precalculating weighted relative entropy (WRENT) scores, secondary structure ensemble free energies¶

For faster downstream analysis, weighted relative entropy (WRENT) scores and the computationally-predicted ensemble free energies of secondary structure around initiation contexts of all ORFs are precalculated and stored.

Ribosome profiling reads contained within ORFs, as well as other ORF and transcript parameters are also determined and stored.

Dataframe columns elaborated in following table:

|  |  |
| --- | --- |
| \*\*Column\*\* | \*\*Description\*\* |
| Transcript | Transcript ID |
| Gene | Gene ID |
| Gene\_Name | Gene Name |
| Gene\_Expression\_FPKM | Expression at gene level (from corresponding RNA-seq data; Tophat + Cufflinks) |
| UTR5\_length | Length of 5' UTR |
| UTR3\_length | Length of 3' UTR |
| UTR5\_reads | Ribosome profiling reads in 5' UTR |
| UTR5\_reads\_trunc | Ribosome profiling reads in 5' UTR, truncated |
| UTR5\_GC | Total GC content of 5' leader |
| num\_uORFs | Number of uORFs (ORFs beginning before CDS) |
| uORFs\_read | Number of reads in each uORF, comma-separated values (CSV) |
| UTR5\_uORF\_reads | Number of reads in 5' leader within uORFs |
| UTR5\_uORF\_tlength | Total length of uORFs within 5' leader |
| uORFs\_length | Length of uORFs, CSV |
| uORFs\_wrent\_score | Calculated WRENT score of uORFs, CSV |
| uORFs\_urent\_score | Calculated URENT (unweighted) score of uORFs, CSV |
| uORFs\_wrent\_seq | Sequences used to calculate WRENT scores, CSV |
| uORFs\_sec\_struct\_EFE\_L | Computationally predicted ensemble free energy of secondary structure around uORF start left (-25), CSV |
| uORFs\_sec\_struct\_EFE\_R | Computationally predicted ensemble free energy of secondary structure around uORF start right (+1), CSV |
| uORFs\_start\_pos\_wrt\_tss | Start position of uORFs with respect to 5' end of transcript, CSV |
| uORFs\_end\_pos\_wrt\_CDS | End position of uORFs with respect to start of CDS, CSV |
| CDS\_read | Number of reads in CDS |
| CDS\_GC | Total GC content of CDS |
| CDS\_length | Length of CDS |
| CDS\_wrent\_score | Calculated WRENT score of CDS |
| CDS\_urent\_score | Calculated URENT score of CDS |
| CDS\_wrent\_seq | Sequence used to calculate WRENT score, CSV |
| CDS\_sec\_struct\_EFE\_L | Computationally predicted ensemble free energy of secondary structure around CDS start, left (-25) |
| CDS\_sec\_struct\_EFE\_R | Computationally predicted ensemble free energy of secondary structure around CDS start, right (+1) |
| ORF\_5CI3 | Annotation of ORF within transcript, if begins in \*\*5\*\*' UTR, is \*\*C\*\*DS, begins with\*\*I\*\*n CDS, or in \*\*3\*\*' UTR, string of letters |
| ORFs\_wrent\_score | Calculated WRENT score of ORFs, CSV |
| ORFs\_urent\_score | Calculated URENT score of ORFs, CSV |
| ORFs\_wrent\_seq | Sequences used to calculate WRENT score, CSV |
| ORFs\_sec\_struct\_EFE\_L | Computationally predicted ensemble free energy of secondary structure around ORF start, left (-25), CSV |
| ORFs\_sec\_struct\_EFE\_R | Computationally predicted ensemble free energy of secondary structure around ORF start, right (+1), CSV |

In [ ]:

```
INDEX = "Transcript"
COLUMNS = ["Gene", "Gene_Name", "Gene_Expression_FPKM", "UTR5_length", "UTR3_length", "UTR5_reads", "UTR5_reads_trunc",
           "UTR5_GC", "num_uORFs", "uORFs_reads", "UTR5_uORF_reads", "UTR5_uORF_tlength", "uORFs_length",
           "uORFs_wrent_score", "uORFs_urent_score", "uORFs_wrent_seq", "uORFs_sec_struct_EFE_L", "uORFs_sec_struct_EFE_R",
           "uORFs_start_pos_wrt_tss", "uORFs_end_pos_wrt_CDS",
           "CDS_reads", "CDS_length", "CDS_GC",
           "CDS_wrent_score", "CDS_urent_score", "CDS_wrent_seq", "CDS_sec_struct_EFE_L", "CDS_sec_struct_EFE_R",
           "ORFs_5CI3", "ORFs_wrent_score", "ORFs_urent_score", "ORFs_wrent_seq",
           "ORFs_sec_struct_EFE_L", "ORFs_sec_struct_EFE_R"]
```

Calculated values are first stored in a dictionary, and a DataFrame is constructed afterward.

In [ ]:

```
# INITIALIZE DICTIONARIES FOR STORAGE
df_main = DataFrame(columns=COLUMNS)
df_main.index.name = INDEX

# FILE ITERATOR FOR .trpedf
trpedf_file_iterator = pd.read_table(TRPE_FILE, converters=CONVERTERS, chunksize=1)
transcript_count2 = 0      # counter
```

A counter counts every 500 transcripts and prints to output.

Initiation contexts for weighted Kozak scoring and secondary structure prediction are first determined using the earlier helper functions. uORFs, CDSes, then all ORFs are scored, with ORFs annotated for whether they begin in the 5' UTR, are the CDS, begin within the CDS, or in the 3' UTR.

An additional dictionary stores the profile of ribosome profiling reads around the starts and ends of transcript CDSes, with equal weight to each transcript, to generate a metaprofile.

In [ ]:

```
# ITERATE OVER .trpedf
for transcript in trpedf_file_iterator:
    
    # Counter for iPython Notebook display, every 100 transcripts
    transcript_count2 += 1
    if transcript_count2 % 100 == 0:
        print "%d transcripts read" % transcript_count2
    
    # Reads in data from each transcript
    RPF_csvProfile = transcript["RPF_csvProfile"][0]
    seq = SEQS[transcript["Transcript"][0]].seq     # get transcript sequence
    if "N" in seq: continue
    ORF_starts = transcript["ORF_starts"][0]
    ORF_ends = transcript["ORF_ends"][0]
    if type(ORF_starts) is np.int64:      # corrects for single-entry
        ORF_starts = (ORF_starts,)
        ORF_ends = (ORF_ends,)
    CDS = transcript["CDS"][0]
    ORFs = zip(ORF_starts, ORF_ends)
    uORFs = [ORF for ORF in ORFs if ORF[0] < CDS[0]]  # uORFs defined as beginning before CDS

    # Determines initiation contexts for weighted Kozak scoring of:
    # uORFs
    uORF_wrent_seqs = [wrent_context_seq(seq, uORF, WRENT_CONTEXT) for uORF in uORFs]
        
    # CDS
    CDS_wrent_seq = wrent_context_seq(seq, CDS, WRENT_CONTEXT)
    
    # All ORFs
    ORF_wrent_seqs = [wrent_context_seq(seq, ORF, WRENT_CONTEXT) for ORF in ORFs]
        
    # Determines initiation contexts for secondary structure prediction of:
    # uORFs
    uORF_ss_seqs_l = [ss_context_seq(seq, uORF, SS_CONTEXT_L) for uORF in uORFs]
    uORF_ss_seqs_r = [ss_context_seq(seq, uORF, SS_CONTEXT_R) for uORF in uORFs]
    
    # CDS
    CDS_ss_seq_l = ss_context_seq(seq, CDS, SS_CONTEXT_L)
    CDS_ss_seq_r = ss_context_seq(seq, CDS, SS_CONTEXT_R)
    
    # All ORFs
    ORF_ss_seqs_l = [ss_context_seq(seq, ORF, SS_CONTEXT_L) for ORF in ORFs]
    ORF_ss_seqs_r = [ss_context_seq(seq, ORF, SS_CONTEXT_R) for ORF in ORFs]
    
    # Calculate and store values in main dictionary
    entry = {}
    for j in ("Gene", "Gene_Name", "Gene_Expression_FPKM"):
        entry[j] = transcript[j][0]
    entry["UTR5_length"] = CDS[0]
    entry["UTR3_length"] = len(RPF_csvProfile) - CDS[1]
    entry["UTR5_reads"] = sum(RPF_csvProfile[:CDS[0]])
    entry["UTR5_reads_trunc"] = sum(RPF_csvProfile[:CDS[0]-ORF_END_TRIM])
    entry["UTR5_GC"] = 0.0 if CDS[0] == 0 else float(seq[:CDS[0]].count("G") + seq[:CDS[0]].count("C")) / CDS[0]
    entry["num_uORFs"] = len(uORFs)

    entry["uORFs_reads"] = [sum(RPF_csvProfile[uORF[0]:uORF[1]-ORF_END_TRIM]) for uORF in uORFs]
    
    transcript_UTR5_uORF_segments = UTR5_uORF_segments(CDS, uORFs)
    entry["UTR5_uORF_reads"] = sum([i for i, j in zip(RPF_csvProfile, transcript_UTR5_uORF_segments) if j])
    entry["UTR5_uORF_tlength"] = sum(transcript_UTR5_uORF_segments)
    
    entry["uORFs_length"] = [uORF[1] - uORF[0] for uORF in uORFs]
    entry["uORFs_wrent_score"] = [wrent_score_seq(en_score, init_seq) \
                                            for init_seq in uORF_wrent_seqs]
    entry["uORFs_urent_score"] = [wrent_score_seq(en_unweighted_score, init_seq) \
                                            for init_seq in uORF_wrent_seqs]
    entry["uORFs_wrent_seq"] = uORF_wrent_seqs
    entry["uORFs_sec_struct_EFE_L"] = [RNA.pf_fold(init_seq)[1] for init_seq in uORF_ss_seqs_l]
    entry["uORFs_sec_struct_EFE_R"] = [RNA.pf_fold(init_seq)[1] for init_seq in uORF_ss_seqs_r]
    entry["uORFs_start_pos_wrt_tss"] = [uORF[0] for uORF in uORFs]
    entry["uORFs_end_pos_wrt_CDS"] = [uORF[1] - CDS[0] for uORF in uORFs]

    entry["CDS_reads"] = sum(RPF_csvProfile[CDS[0]:CDS[1]-ORF_END_TRIM])
    entry["CDS_GC"] = 0.0 if CDS[0] == CDS[1] else float(seq[CDS[0]:CDS[1]].count("G") \
                                                         + seq[CDS[0]:CDS[1]].count("C")) \
                                                         / (CDS[1] - CDS[0])
    entry["CDS_length"] = CDS[1] - CDS[0]
    entry["CDS_wrent_score"] = wrent_score_seq(en_score, CDS_wrent_seq)
    entry["CDS_urent_score"] = wrent_score_seq(en_unweighted_score, CDS_wrent_seq)
    entry["CDS_wrent_seq"] = CDS_wrent_seq
    entry["CDS_sec_struct_EFE_L"] = RNA.pf_fold(CDS_ss_seq_l)[1]
    entry["CDS_sec_struct_EFE_R"] = RNA.pf_fold(CDS_ss_seq_r)[1]

    # 5CIS refers to annotation of ORF as beginning in 5' UTR ('5'),
    # being the CDS ('C'),
    # beginning in the CDS ('I'), or
    # beginning in the 3' UTR ('3')
    entry["ORFs_5CI3"] = "".join(['C' if ORF_start == CDS[0] else \
                                  '5' if ORF_start < CDS[0] else \
                                  'I' if CDS[0] < ORF_start < CDS[1] else \
                                  '3' for ORF_start in ORF_starts])
    entry["ORFs_wrent_score"] = [wrent_score_seq(en_score, init_seq) \
                                           for init_seq in ORF_wrent_seqs]
    entry["ORFs_urent_score"] = [wrent_score_seq(en_unweighted_score, init_seq) \
                                           for init_seq in ORF_wrent_seqs]
    entry["ORFs_wrent_seq"] = ORF_wrent_seqs
    entry["ORFs_sec_struct_EFE_L"] = [RNA.pf_fold(init_seq)[1] for init_seq in ORF_ss_seqs_l]
    entry["ORFs_sec_struct_EFE_R"] = [RNA.pf_fold(init_seq)[1] for init_seq in ORF_ss_seqs_r]
    
    df_main.loc[transcript["Transcript"][0]] = Series(entry)
```

In [ ]:

```
df_main.to_csv(DATA_DIR + stage + "_main.df", sep="\t")
```
