# Supplementary material for: Conservation of uORF repressiveness and sequence features in mouse, human and zebrafish
Source: Nat Commun. 2016 May 24;7:11663. doi: 10.1038/ncomms11663 (PMC4890304; doi:10.1038/ncomms11663)
Supplement: Supplementary Software — Conversions of iPython/Jupyter notebooks documenting all analysis for manuscript. Latest versions of iPython/Jupyer notebooks are available at https://github.com/chewgl/uORF_repressiveness_supplemental [file ncomms11663-s2.zip › Fig 1 - Trends in uORF sequence characteristics - Human.html]

Fig 1 - Trends in uORF sequence characteristics - Human


# Fig 1 - Trends in uORF sequence characteristics¶

## Dataset¶

In [1]:

```
# s = "mm"
# stage = "mES"
# ASSEMBLY = "GRCm38_ens"

# s = "dr"
# stage = "Shield"
# ASSEMBLY = "Zv9_ens"

s = "hs"
stage = "HeLa"
ASSEMBLY = "GRCh37_ens"
```

## Imports, parameters and functions¶

Parameters for filtering transcripts are defined in the following cell.

Due to incomplete annotation of transcripts, as well as to analyze transcripts with canonical forms of translation initiation (e.g. not TISU translation - Elfakess et al. NAR 2011) transcripts are filtered to have minimum UTR lengths.

In addition, to reduce stochastic noise from analysis of sparse data, transcripts were required to be expressed at a threshold of FPKM > 0.1, a minimum CDS length (containing a minumum number of ribosome profiling reads); and where translation of uORFs were analyzed quantitatively, a minimum uORF length.

In [2]:

```
# FILTER PARAMETERS
UTR5_LENGTH_MIN = 25
UTR3_LENGTH_MIN = 25

FPKM_MIN = 0.1
CDS_LENGTH_MIN = 100
CDS_READS_MIN = 1

UORF_LENGTH_MIN = 20
UORF_FROM_TRANSCRIPT_START_MIN = UTR5_LENGTH_MIN
UORF_READS_MIN = 1

ORF_END_TRIM = 10
```

A number of external Python packages are used:  
**pandas** for data analysis  
**matplotlib** (Version 1.4.3) and **seaborn** for plotting  
**numpy** for numerical array manipulations  
**scipy.stats** and **statsmodels** for statistics  
**sklearn** for machine-learning  
**Biopython** and **weblogo** for sequence manipulation and logo display

In [3]:

```
# IMPORTS
import corebio
import weblogolib

import matplotlib as mpl
import matplotlib.pyplot as plt
import numpy as np
import pandas as pd
import seaborn.apionly as sns

from Bio import SeqIO
from IPython.display import display, HTML, Markdown, Image
from ast import literal_eval
from numpy import argmax, mean, log10, log2, inf, nan, array, float64, subtract, multiply, divide, sign
from pandas import Panel, DataFrame, Series
from pandas.stats.moments import rolling_mean
from scipy.stats import spearmanr, ks_2samp, pearsonr, linregress, zscore, kendalltau, kde
from scipy.stats.mstats import trimboth
from sklearn.linear_model import Ridge, RidgeCV
from statsmodels.distributions import ECDF
from statsmodels.nonparametric.smoothers_lowess import lowess

%matplotlib inline
```

Some default plotting parameters and colors are defined in the following cells.

In [4]:

```
pd.options.display.mpl_style = 'default'
pd.options.mode.chained_assignment = None
mpl.rcParams['figure.figsize'] = 8, 6
mpl.rcParams['axes.labelsize'] = 12
mpl.rcParams['axes.titlesize'] = 16
mpl.rcParams['axes.facecolor'] = "#fdfdfd"
mpl.rcParams['grid.alpha'] = 0.5
mpl.rcParams['legend.fontsize'] = 12
mpl.rcParams['font.family'] = 'sans-serif'
mpl.rcParams['figure.autolayout'] = True
mpl.rcParams['savefig.dpi'] = 50
```

In [5]:

```
C_NT = ("#00d700", "#df1f00", "#0226cc", "#ffb700")
C_UORF = ('#b30000', '#e34a33', '#fc8d59', '#fdbb84', '#fdd49e')
C_CDS = ("#105e47", "#157e5f", "#1b9e77", "#48b192", "#76c4ad")
C_RATIO = "#7570b3"
C_BG = ("#000000", "#525252", "#969696", "#d9d9d9")
C_SP = {"hs": "#97543a", "mm": "#546079", "dr": "#3d7463"}
C_SP_BAR = {"hs": "#fc8d62", "mm": "#8da0cb", "dr": "#66c2a5"}
C_NOBIAS = "#ffd92f"
C_SCATTER = "#737373"
C_TREND = "#e41a1c"
C_TLOC = {"5' UTR": "#d95f02", "CDS start": "#1b9e77", "CDS internal": "#e7298a", "3' UTR": "#e6ab02"}
C_STARTS = ("#276419", "#4d9221", "#7fbc41")
C_STOPS = ("#8e0152", "#c51b7d", "#de77ae")
```

## Helper functions¶

In [6]:

```
def scatter_linear_trend(x, y, x_label, y_label, plot_trend=True, corr_table=True):
    data = DataFrame({"x": x, "y": y}).dropna()
    
    plt.figure()
    plt.scatter(data.x, data.y, alpha=0.25, c=C_SCATTER)

    mod = Ridge(normalize=False).fit(zip(*[data.x,]), data.y)
    x1, x2 = (np.percentile(data.x, 2.5), np.percentile(data.x, 97.5))
    y1, y2 = mod.predict([(x1,), (x2,)])
    
    plt.ylim(np.percentile(data.y, 1), np.percentile(data.y, 99))
    plt.xlabel(x_label)
    plt.ylabel(y_label)
    
    if plot_trend:
        plt.plot((x1, x2), (y1, y2), c=C_TREND, lw=2)

    if corr_table:
        to_display = DataFrame(columns=["Correlation\ncoefficient", "p-value"])

        corr, p = pearsonr(data.x, data.y)
        to_display.loc["Pearson linear"] = {"Correlation\ncoefficient": corr,
                                    "p-value": p}
        corr, p = spearmanr(data.x, data.y)
        to_display.loc["Spearman rank"] = {"Correlation\ncoefficient": corr,
                                  "p-value": p}

        display(Markdown(y_label + " against " + x_label))
        display(to_display.applymap('{:,.4g}'.format))
    
    return abs(y2 - y1), sign(y2 - y1)
```

## Reading in ORF characteristics data¶

The following cells read the ORF characteristic data into DataFrames.

ORF characteristics are read in from "\_main.df" files. A number of fields are comma-separated, and will need to be literally evaluated.

Secondary structure ensemble folding energies (EFEs) were read in from "\_mean\_ssefes.df" files.

CDS TE was calculated to determine correlation with initiation context scoring metrics (Unweighted and Weighted relative entropy scores).

In [7]:

```
DATA_DIR = "./data/" + s + "/"
ANNOTATIONS_DIR = "./annotations/"
```

In [8]:

```
CONVERTERS = {i:literal_eval for i in ("uORFs_reads", "uORFs_length", "uORFs_wrent_score", "uORFs_urent_score",
                                       "uORFs_wrent_seq", "uORFs_sec_struct_EFE_L", "uORFs_sec_struct_EFE_R",
                                       "uORFs_start_pos_wrt_tss", "uORFs_end_pos_wrt_CDS",
                                       "ORFs_wrent_score", "ORFs_urent_score", "ORFs_wrent_seq",
                                       "ORFs_sec_struct_EFE_L", "ORFs_sec_struct_EFE_R")}
```

In [9]:

```
mean_ssefes = pd.read_table(DATA_DIR + s + "_mean_ssefes.df", sep="\t", index_col=[0])
```

In [10]:

```
windows = [25, 30, 35, 40]
df_main = pd.read_csv(DATA_DIR + stage + "_main.df", index_col="Transcript", sep="\t", converters=CONVERTERS)
for window in windows:
    for pos in ("UTR5", "CDS"):
        field = pos + "_mean_ssefe_" + str(window)
        df_main[field] = mean_ssefes[field][mean_ssefes.index.isin(df_main.index)]
        
df_main["CDS_TE"] = df_main.CDS_reads / \
                    df_main.CDS_length.subtract(ORF_END_TRIM) / \
                    df_main.Gene_Expression_FPKM
```

Transcripts are filtered for minimum UTR lengths, gene expression, CDS length, and ribosome profiling reads in CDS.

In [11]:

```
df_main_filtered = df_main[(df_main.UTR5_length >= UTR5_LENGTH_MIN) &
                           (df_main.UTR3_length >= UTR3_LENGTH_MIN) &
                           (df_main.Gene_Expression_FPKM >= FPKM_MIN) &
                           (df_main.CDS_length >= CDS_LENGTH_MIN) &
                           (df_main.CDS_reads >= CDS_READS_MIN)]
```

## Reading in profile data¶

The following cells read secondary structure folding data and sorts them by number of uORFs per transcript.

In [12]:

```
# RNAs were folded in windows of sizes 25, 30, 35 and 40 nucleotides
# Choose which window size to analyze (35nt ~= size of ribosome footprint)
windows = (25, 30, 35, 40)
window = 35
```

In [13]:

```
ATG_5CI3_profiles = pd.read_table(DATA_DIR + s + "_" + str(window)
                                  + "_RNA_fold_ATG_profiles_by_pos",
                                  sep="\t", index_col=0)
ATG_5CI3_profiles.columns = ["3' UTR", "5' Leader", "CDS", "CDS Internal"]

uORF_profiles = data = pd.read_table(DATA_DIR + s + "_" + str(window)
                                     + "_RNA_fold_uORF_profiles_by_num",
                                     sep="\t", index_col=0)
data.columns = ["1 uORF", "2 uORFs", "3 uORFs", "4+ uORFs"]
```

The following cells read nucleotide / tri-nucleotide frequency data, for all three species, and calculates biases.

In [14]:

```
species = ("hs", "mm", "dr")
species_label = {"hs": "Human", "mm": "Mouse", "dr": "Zebrafish"}
species_stage = {"hs": "HeLa", "mm": "mES", "dr": "Shield"}

TRINTS = [nt1+nt2+nt3 for nt1 in "ATCG" for nt2 in "ATCG" for nt3 in "ATCG"]
STOP_TRINTS = ["TAG", "TGA", "TAA"]
```

In [15]:

```
uORF_start_trint, uORF_end_trint = [{}, {}]
trint_freq_sp, trint_pos_expected_freq_sp = [{}, {}]

for sp in species:
    nt_freq = DataFrame.from_csv("./data/" + sp + "/" + species_stage[sp] + "_nt_freq.df", sep="\t")
    trint_freq = DataFrame.from_csv("./data/" + sp + "/" + species_stage[sp] + "_trint_freq.df", sep="\t")
    
    nt_freq.columns = map(int, nt_freq.columns)
    trint_freq.columns = map(int, trint_freq.columns)
    
    total_bkgrnd_nt_freq = nt_freq.mean(axis='columns') / nt_freq.mean(axis='columns').sum()
    pos_bkgrnd_nt_freq = nt_freq / nt_freq.sum(axis='rows')
    
    trint_total_expected_freq = Series({trint: np.prod([total_bkgrnd_nt_freq[nt]\
                                                        for nt in trint])
                                        for trint in TRINTS})
    trint_pos_expected_freq = DataFrame({i:Series({trint: np.prod([pos_bkgrnd_nt_freq[i + pos][nt] \
                                                                   for pos, nt in enumerate(trint)]) \
                                                         for trint in TRINTS}) for i in trint_freq})
    trint_freq = trint_freq / trint_freq.sum(axis='rows')
    
    trint_freq_sp[sp] = trint_freq
    trint_pos_expected_freq_sp[sp] = trint_pos_expected_freq
    
    uORF_start_trint[sp] = log2((trint_freq / trint_pos_expected_freq).T["ATG"])
    uORF_end_trint[sp] = log2((trint_freq / trint_pos_expected_freq).T[STOP_TRINTS]).mean(axis=1)
```

The following cell reads in uORF end frequency data and calculate uORF end depletion (absolute reduction) and bias (relative reduction).

In [16]:

```
uORF_end_freq = dict()
LOOPS = 100

for sp in species:
    uORF_end_freq[sp] = DataFrame.from_csv("./data/" + sp + "/" + species_stage[sp] + "_uORF_ends.df", sep="\t")
    uORF_end_freq[sp].uORF_ends_exp = uORF_end_freq[sp].uORF_ends_exp / LOOPS
    uORF_end_freq[sp]["bias"] = log2(uORF_end_freq[sp].uORF_ends_obs / uORF_end_freq[sp].uORF_ends_exp)
    uORF_end_freq[sp]["dep"] = uORF_end_freq[sp].uORF_ends_obs - uORF_end_freq[sp].uORF_ends_exp
```

The following cell reads in the PSSMs for weighted and unweighted motifs around uORF and CDS starts, creates logos using **weblogolib**.

In [17]:

```
pssm = DataFrame.from_csv(DATA_DIR + stage + "_pssm.df", sep="\t")
unweighted_pssm = DataFrame.from_csv(DATA_DIR + stage + "_pssm_unweighted.df", sep="\t")
uORF_pssm = DataFrame.from_csv(DATA_DIR + stage + "_pssm_uORF.df", sep="\t")
uORF_unweighted_pssm = DataFrame.from_csv(DATA_DIR + stage + "_pssm_uORF_unweighted.df", sep="\t")
```

In [18]:

```
logo_pngs = {}
for data, label, xaxis_label, y in zip((pssm, unweighted_pssm, uORF_pssm, uORF_unweighted_pssm),
                                       ("Weighted", "Unweighted", "uORF weighted", "uORF unweighted"),
                                       ("CDS", "CDS", "uORF", "uORF"),
                                       (0.6, 0.6, 0.6, 0.6)):
    pssm_np_array = np.array(data.loc[["A", "C", "G", "T"]]).T
    pssm_np_array = np.concatenate((pssm_np_array[:10], np.ones((3, 4)), pssm_np_array[10:]), axis=0)
    
    logo = weblogolib.LogoData.from_counts(alphabet=corebio.seq.unambiguous_dna_alphabet, counts=pssm_np_array)
    options = weblogolib.LogoOptions(fineprint=None, resolution=195, yaxis_scale=y, first_index=-10,
                                     ticmarks=0.1, color_scheme=weblogolib.classic,
                                     yaxis_tic_interval=0.5, yaxis_minor_tic_ratio=5,
                                     xaxis_label="Position w.r.t. " + xaxis_label + " start",
                                     stack_aspect_ratio=15)
    logo_out = weblogolib.LogoFormat(logo, options)
    logo_pngs[label] = weblogolib.png_formatter(logo, logo_out)
```

## Defining various transcriptome subsets¶

In [19]:

```
# Define various transcript sets based on number of uORFs
no_uORFs = df_main_filtered[df_main_filtered.num_uORFs == 0]
with_uORFs = df_main_filtered[df_main_filtered.num_uORFs > 0]

one_uORF = df_main_filtered[df_main_filtered.num_uORFs == 1]
two_uORF = df_main_filtered[df_main_filtered.num_uORFs == 2]
three_uORF = df_main_filtered[df_main_filtered.num_uORFs == 3]
more_uORF = df_main_filtered[df_main_filtered.num_uORFs >= 4]
```

In [20]:

```
# Creates set of 1 non-overlapping uORF
one_non_overlapping_uORF = df_main_filtered[(df_main_filtered.num_uORFs == 1)]   # 1 uORF

# Since set has only transcripts with 1 uORF, "singularize" some columns of data
for variable in ("uORFs_reads", "uORFs_length", "uORFs_wrent_score",
                 "uORFs_sec_struct_EFE_L", "uORFs_sec_struct_EFE_R",
                 "uORFs_start_pos_wrt_tss", "uORFs_end_pos_wrt_CDS"):
    one_non_overlapping_uORF[variable] = one_non_overlapping_uORF[variable].apply(lambda x:x[0])

# Filter set for non-overlapping uORF, with uORFs of minimum length, reads and distance from transcript start
one_non_overlapping_uORF = one_non_overlapping_uORF[\
    (one_non_overlapping_uORF.uORFs_end_pos_wrt_CDS < 0) &
    (one_non_overlapping_uORF.uORFs_reads >= UORF_READS_MIN) &
    (one_non_overlapping_uORF.uORFs_length >= UORF_LENGTH_MIN) &
    (one_non_overlapping_uORF.uORFs_start_pos_wrt_tss >= UORF_FROM_TRANSCRIPT_START_MIN)]
display(Markdown("Number of one non-overlapping uORF Transcripts = %d" % len(one_non_overlapping_uORF)))

# Calculate uORF TEs for set
one_non_overlapping_uORF["uORF_TE"] = one_non_overlapping_uORF.uORFs_reads / \
                                      one_non_overlapping_uORF.uORFs_length.subtract(ORF_END_TRIM) / \
                                      one_non_overlapping_uORF.Gene_Expression_FPKM
```

Number of one non-overlapping uORF Transcripts = 677

## Figures¶

### uORF initiation context sequence¶

**Fig 1a and Supp Fig 1a insert:** Relative entropy (weighted) scoring matrix represented as a sequence logo

In [21]:

```
Image(logo_pngs["Weighted"])
```

Out[21]:

**Supp Fig 1b insert:** Relative entropy (unweighted) scoring matrix represented as a sequence logo

In [22]:

```
Image(logo_pngs["Unweighted"])
```

Out[22]:

**Supp Fig 1a-b:** Scatter plots of CDS TE against CDS initiation context relative entropy score (weighted and unweighted; WRENT and URENT respectively), in transcripts with uORFs

In [23]:

```
for data, if_weighted in zip((with_uORFs.CDS_wrent_score, with_uORFs.CDS_urent_score),
                             ("Weighted Relative ENTropy (WRENT)", "Unweighted Relative ENTropy (URENT)")):

    y_range = scatter_linear_trend(data, log2(with_uORFs.CDS_TE),
                                   "CDS " + if_weighted + " Score", "log2 CDS TE")
    plt.xlim(-7, 7)
    plt.ylim(-10, 0)
    plt.title("Filtered transcripts with uORFs")
    display(Markdown("{:2.2f}-fold change over 95% of data.".format(2**y_range[0])))
```

log2 CDS TE against CDS Weighted Relative ENTropy (WRENT) Score

|  | Correlation coefficient | p-value |
| --- | --- | --- |
| Pearson linear | 0.083 | 3.354e-11 |
| Spearman rank | 0.09316 | 9.679e-14 |

1.60-fold change over 95% of data.

log2 CDS TE against CDS Unweighted Relative ENTropy (URENT) Score

|  | Correlation coefficient | p-value |
| --- | --- | --- |
| Pearson linear | 0.04909 | 8.964e-05 |
| Spearman rank | 0.05315 | 2.223e-05 |

1.32-fold change over 95% of data.

```
c:\Anaconda2\lib\site-packages\matplotlib\collections.py:590: FutureWarning: elementwise comparison failed; returning scalar instead, but in the future will perform elementwise comparison
  if self._edgecolors == str('face'):
c:\Anaconda2\lib\site-packages\matplotlib\figure.py:1653: UserWarning: This figure includes Axes that are not compatible with tight_layout, so its results might be incorrect.
  warnings.warn("This figure includes Axes that are not "
```

**Fig 1a inset**: WRENT scoring matrix of uORFs represented as sequence logo.

In [24]:

```
Image(logo_pngs["uORF weighted"])
```

Out[24]:

**Fig 1b:** WRENT scores at all AUGs in transcripts, as classified by their position within the transcript: within the 5' UTR, defining the start of the CDS, within the CDS, or within the 3' UTR. Visualized as a CDF plot.

In [25]:

```
#%% ALL FILTERED WITH UORF TRANSCRIPTS ANALYSIS
ORFs_by_position = [with_uORFs.ORFs_5CI3.apply(lambda x:np.array([i == pos for i in x]))\
                    for pos in "5CI3"]
wrent_seq_lengths = with_uORFs.ORFs_wrent_seq.apply(lambda x:np.array([len(i) == 20 for i in x]))
```

In [26]:

```
score, xlabel = ("wrent", "WRENT score of ORF")
labels = ("5' UTR", "CDS start", "CDS internal", "3' UTR")
scores_by_position = [np.concatenate([np.array(x)[y & z] for x, y, z in \
                      zip(with_uORFs["ORFs_" + score + "_score"], pos, wrent_seq_lengths)])\
                      for pos in ORFs_by_position]

plt.figure()
label_n = [i[0] + " (n=" + str(i[1]) + ")" \
           for i in zip(labels, [len(i) for i in scores_by_position])]

for data, labelled, label in zip(scores_by_position, label_n, labels):
    ecdf = ECDF(data)
    plt.plot(ecdf.x, ecdf.y, label=labelled, c=C_TLOC[label], lw=2)

ecdf_CDS = ECDF(scores_by_position[1])
ecdf_uORF = ECDF(scores_by_position[0])
CDS_start_med = [x for x, y in zip(ecdf_CDS.x, ecdf_CDS.y) if y < 0.5][-1]
uORF_start_med = [x for x, y in zip(ecdf_uORF.x, ecdf_uORF.y) if y < 0.5][-1]
uORF_CDS_med_val = [y for x, y in zip(ecdf_uORF.x, ecdf_uORF.y) if x > CDS_start_med][0]
plt.plot((CDS_start_med, CDS_start_med), (0, uORF_CDS_med_val), "--", c="grey")
plt.plot((-7, CDS_start_med), (0.5, 0.5), "--", c="grey")
plt.plot((uORF_start_med, uORF_start_med), (0, 0.5), "--", c="grey")
plt.plot((-7, CDS_start_med), (uORF_CDS_med_val, uORF_CDS_med_val), "--", c="grey")
    
plt.legend(title="ORF location", loc=0)
plt.xlabel(xlabel)
plt.xlim(-7, 6)
plt.ylabel("Cumulative distribution")
print uORF_CDS_med_val, uORF_start_med, CDS_start_med - uORF_start_med
```

```
0.801097200731 -1.0865993599 2.22521097085
```

**Supp Fig 1e:** WRENT scores of uORF and CDS AUGs, subsetted by the number of uORFs in each transcript, visualized as a CDF plot.

In [27]:

```
num_uORFs = (no_uORFs, one_uORF, two_uORF, three_uORF, more_uORF)

uORFs_by_uORF_num = [num_uORF.ORFs_5CI3.apply(lambda x:np.array([i == "5" for i in x])) \
                     for num_uORF in num_uORFs[1:]]

wrent_seq_lengths = [num_uORF.ORFs_wrent_seq.apply(lambda x:np.array([len(i) == 20 for i in x])) \
                     for num_uORF in num_uORFs[1:]]

uORF_wrent_by_uORF_num = [np.concatenate([np.array(x)[y & z] for x, y, z in \
                          zip(num_uORF.ORFs_wrent_score, pos, wrent_seq_length)])\
                          for pos, num_uORF, wrent_seq_length in \
                          zip(uORFs_by_uORF_num, num_uORFs[1:], wrent_seq_lengths)]
```

In [28]:

```
labelleds = ("no uORFs", "1 uORF", "2 uORFs", "3 uORFs", "4+ uORFs")
CDS_lines = {}
uORF_lines = {}

for by_uORFs, labelled, color in zip(num_uORFs, labelleds, C_CDS):
    ecdf = ECDF(by_uORFs.CDS_wrent_score)
    CDS_lines[labelled], = plt.plot(ecdf.x, ecdf.y,
                                    label=labelled + " (n=" + str(len(by_uORFs)) + ")",
                                    c=color, lw=2)

first_legend = plt.legend(handles=[CDS_lines[labelled] for labelled in labelleds],
                          title="CDS WRENT scores", loc="lower right")
ax = plt.gca().add_artist(first_legend)

for data, labelled, color in zip(uORF_wrent_by_uORF_num, labelleds[1:], C_UORF):
    ecdf = ECDF(data)
    uORF_lines[labelled], = plt.plot(ecdf.x, ecdf.y,
                                     label=labelled + " (n=" + str(len(data)) + ")",
                                     c=color, lw=2)

labelled = "1 uORF"


ecdf_CDS = ECDF(num_uORFs[1].CDS_wrent_score)
ecdf_uORF = ECDF(uORF_wrent_by_uORF_num[0])
CDS_start_med = [x for x, y in zip(ecdf_CDS.x, ecdf_CDS.y) if y < 0.5][-1]
uORF_start_med = [x for x, y in zip(ecdf_uORF.x, ecdf_uORF.y) if y < 0.5][-1]
uORF_CDS_med_val = [y for x, y in zip(ecdf_uORF.x, ecdf_uORF.y) if x > CDS_start_med][0]
plt.plot((CDS_start_med, CDS_start_med), (0, uORF_CDS_med_val), "--", c="grey")
plt.plot((-7, CDS_start_med), (0.5, 0.5), "--", c="grey")
plt.plot((uORF_start_med, uORF_start_med), (0, 0.5), "--", c="grey")
plt.plot((-7, CDS_start_med), (uORF_CDS_med_val, uORF_CDS_med_val), "--", c="grey")

    
    
plt.legend(handles=[uORF_lines[labelled] for labelled in labelleds[1:]],
           title="uORF WRENT scores", loc="upper left")
plt.xlim(-7, 6)
plt.xlabel("WRENT score")
plt.ylabel("Cumulative distribution")
print uORF_CDS_med_val, uORF_start_med, CDS_start_med - uORF_start_med
```

```
0.851683348499 -1.71677449433 2.89646687451
```

### uORF initiation context secondary structure¶

Analyses of the secondary structure trends of uORF initiation contexts, using ensemble free energy (EFE) as predicted by the Vienna RNA package. EFEs were calculated for 35 nucleotide windows unless otherwise stated (e.g. Figs S2a, c)

**Fig 1c**: Mean secondary structure EFE profiles around all AUGs as classified by their position within the transcript, determined *in silico* (using the ViennaRNA package) by folding 35-nucleotide sliding windows across all transcripts.

In [29]:

```
window = 35
data = pd.read_table(DATA_DIR + s + "_" + str(window) + "_RNA_fold_ATG_profiles_by_pos",
                     sep="\t", index_col=0)
data.columns = ["3' UTR", "5' UTR", "CDS start", "CDS internal"]
for pos in ["3' UTR", "CDS internal", "5' UTR", "CDS start"]:
    data[pos].plot(c=C_TLOC[pos], lw=2)
plt.legend(title="ATG position", loc="upper right", ncol=4)
plt.xlabel("window leftmost position w.r.t. ATG")
plt.ylabel("Mean sec struct EFE")
plt.ylim(-9, -4)

plt.plot((-25, -25), (-9, -4), "--", lw=1, c="red")
plt.plot((1, 1), (-9, -4), "--", lw=1, c="red")
```

Out[29]:

```
[<matplotlib.lines.Line2D at 0x2953cf60>]
```

**Supp Fig 2a**: Mean secondary structure EFE profiles around CDS starts using sliding windows of various sizes.

In [30]:

```
for window, color in zip(windows, C_BG[::-1]):
    data = pd.read_table(DATA_DIR + s + "_" + str(window) + "_RNA_fold_ATG_profiles_by_pos",
                         sep="\t", index_col=0)
    data.columns = ["3' UTR", "5' Leader", str(window), "CDS Internal"]
    data[str(window)].plot(c=color, lw=2)
plt.legend(title="Window size", loc="upper right", ncol=4)
plt.xlabel("Window leftmost position w.r.t. CDS start")
plt.ylabel("Mean sec struct EFE")
plt.ylim(-11, -3)
```

Out[30]:

```
(-11, -3)
```

**Supp Fig 2b:** Mean secondary structure EFE profiles over uORF and CDS starts, for transcripts with varying number of uORFs. Red lines indicate where correlation is locally maximal.

In [31]:

```
data = pd.read_table(DATA_DIR + s + "_" + str(window) + "_RNA_fold_uORF_profiles_by_num",
                     sep="\t", index_col=0)
data.columns = ["1 uORF", "2 uORFs", "3 uORFs", "4+ uORFs"]
for pos, color in zip(data.columns, C_UORF):
    data[pos].plot(c=color, lw=2)

data = pd.read_table(DATA_DIR + s + "_" + str(window) + "_RNA_fold_ATG_profiles_by_pos",
                     sep="\t", index_col=0)
data.columns = ["3' UTR", "5' UTR", "CDS start", "CDS internal"]
data["CDS start"].plot(c=C_TLOC["CDS start"])

plt.plot((-25, -25), (-12, -8), "--", lw=1, c="red")
plt.plot((1, 1), (-12, -8), "--", lw=1, c="red")

plt.legend(title="Number of uORFs", loc="lower right")
plt.xlabel("Window leftmost position w.r.t. AUG")
plt.ylabel("Mean sec struct EFE")
```

Out[31]:

```
<matplotlib.text.Text at 0x2a8cb4a8>
```

**Supp Fig 2c:** Correlation between secondary structure EFE and CDS TE for various positions around CDS start. Red lines indicate where correlation is locally maximal for a 35 nucleotide window, beginning at -25 and +1.

In [32]:

```
corr = DataFrame(columns=windows, index=np.arange(-50, 50))
CDS_TE = log2(no_uORFs.CDS_TE)
for window in windows:
    ssefe_CDS_profile = DataFrame.from_csv(DATA_DIR + s + "_" + str(window)
                                           + "_RNA_fold_CDS_start_profiles",
                                           sep="\t")
    for win_left in range(-50, 50):
        CDS_ssefe_points = ssefe_CDS_profile.T[win_left][CDS_TE.index].dropna()
        corr[window][win_left] = pearsonr(CDS_ssefe_points, CDS_TE[CDS_ssefe_points.index])[0]

for window, color in zip(windows, C_BG[::-1]):
    corr[window].plot(c=color, label=str(window), lw=2)
    plt.legend(title="window size", loc="lower right")

    plt.plot((-25, -25), (0, 0.22), "--", lw=1, c="red")
    plt.plot((1, 1), (0, 0.22), "--", lw=1, c="red")

    plt.xlabel("Window leftmost position w.r.t. CDS start")
    plt.ylabel("Correlation between sec struct EFE and CDS TE")
    plt.ylim(0, 0.22)
    #plt.xlim(-30, 5)
```

Scatter plots depicting correlation between secondary structure EFE and CDS TE at -25 and +1 w.r.t. the CDS start.

In [33]:

```
window = 35
ssefe_CDS_profile = DataFrame.from_csv(DATA_DIR + s + "_" + str(window)
                                       + "_RNA_fold_CDS_start_profiles",
                                       sep="\t")

CDS_ssefe_points = ssefe_CDS_profile.T[-25][CDS_TE.index].dropna()
scatter_linear_trend(CDS_ssefe_points, CDS_TE[CDS_ssefe_points.index],
                     "CDS sec struct EFE at -25", "log10 CDS TE")
plt.xlim(-20, 0)
plt.ylim(-9, 0)

CDS_ssefe_points = ssefe_CDS_profile.T[1][CDS_TE.index].dropna()
scatter_linear_trend(CDS_ssefe_points, CDS_TE[CDS_ssefe_points.index],
                     "CDS sec struct EFE at +1", "log10 CDS TE")
plt.xlim(-20, 0)
plt.ylim(-9, 0)
```

log10 CDS TE against CDS sec struct EFE at -25

|  | Correlation coefficient | p-value |
| --- | --- | --- |
| Pearson linear | 0.1606 | 2.158e-26 |
| Spearman rank | 0.1628 | 4.42e-27 |

log10 CDS TE against CDS sec struct EFE at +1

|  | Correlation coefficient | p-value |
| --- | --- | --- |
| Pearson linear | 0.1704 | 1.436e-29 |
| Spearman rank | 0.1722 | 3.573e-30 |

Out[33]:

```
(-9, 0)
```

### Subsetting secondary structure EFE data by number of uORFs.¶

In [34]:

```
num_uORFs = (no_uORFs, one_uORF, two_uORF, three_uORF, more_uORF)

labels = ("5' UTR", "CDS start", "CDS internal", "3' UTR")
ORFs_scores_by_position = [[np.concatenate([np.array(x)[y] for x, y in \
                                             zip(with_uORFs["ORFs_" + score].values, pos)])\
                             for pos in ORFs_by_position] \
                            for score in ("sec_struct_EFE_L", "sec_struct_EFE_R")]

uORFs_by_uORF_num = [num_uORF.ORFs_5CI3.apply(lambda x:np.array([i == "5" for i in x])) \
                     for num_uORF in num_uORFs[1:]]

uORF_SSEFE_L_by_uORF_num = [np.concatenate([np.array(x)[y] for x, y in \
                                            zip(num_uORF.ORFs_sec_struct_EFE_L, pos)])\
                            for pos, num_uORF in \
                            zip(uORFs_by_uORF_num, num_uORFs[1:])]

uORF_SSEFE_R_by_uORF_num = [np.concatenate([np.array(x)[y] for x, y in \
                                            zip(num_uORF.ORFs_sec_struct_EFE_R, pos)]) \
                            for pos, num_uORF in \
                            zip(uORFs_by_uORF_num, num_uORFs[1:])]

UTR5_mean_ssefe_35_by_uORF_num = [num_uORF.UTR5_mean_ssefe_35.dropna().values \
                                  for num_uORF in num_uORFs[1:]]

CDS_mean_ssefe_35_by_uORF_num = [num_uORF.CDS_mean_ssefe_35.dropna().values \
                                  for num_uORF in num_uORFs]
```

**Supp Fig 2d**: Secondary structure EFEs at -25 (left) and +1 (right) w.r.t. uORF starts, subdivided by the number of uORFs in a transcript, visualized as a CDF plot. Data for all uORFs and CDSes are additionally plotted with dashed lines.

In [35]:

```
uORFs_by_uORF_num = [num_uORF.ORFs_5CI3.apply(lambda x:np.array([i == "5" for i in x])) \
                     for num_uORF in (one_uORF, two_uORF, three_uORF, more_uORF)]

for lr, lr_label, scores_by_position, u_data in zip(("ORFs_sec_struct_EFE_L", "ORFs_sec_struct_EFE_R"),
                                                    ("(-25 from CDS start)", "(+1 from CDS start)"),
                                                    ORFs_scores_by_position,
                                                    (uORF_SSEFE_L_by_uORF_num, uORF_SSEFE_R_by_uORF_num)):
    plt.figure()
    for data, labelled, color in zip(u_data,
                                     ("1 uORF", "2 uORFs", "3 uORFs", "4+ uORFs"),
                                     C_UORF):
        ecdf = ECDF(data[data != 0])
        plt.plot(ecdf.x, ecdf.y,
                 label=labelled + " (n=" + str(len(data[data != 0])) + ")",
                 c=color, lw=2)

    for data, labelled, color in zip((scores_by_position[0], scores_by_position[1]),
                                     ("All uORFs", "CDSes"),
                                     (C_TLOC["5' UTR"], C_TLOC["CDS start"])):
        ecdf = ECDF(data[data != 0])
        plt.plot(ecdf.x, ecdf.y, "--",
                 label=labelled + " (n=" + str(len(data[data != 0])) + ")",
                 c=color, linewidth=3)

    plt.legend(loc=0)
    plt.xlabel("uORF start sec struct EFE " + lr_label)
    plt.xlim(-18, 0)
    plt.ylabel("Cumulative distribution")
```

**Fig 1d:** Secondary structure EFEs at -25 (left) and +1 (right) w.r.t. ORF starts, subdivided by position in a transcript, visualized as a CDF plot.

In [36]:

```
for score, xlabel, scores_by_position in zip(("sec_struct_EFE_L", "sec_struct_EFE_R"),
                                             ("sec struct EFE (-25 from CDS start)", "sec struct EFE (+1 from CDS start)"),
                                             ORFs_scores_by_position):
    plt.figure()
    labels = ("5' UTR", "CDS start", "CDS internal", "3' UTR")
    label_n = [i[0] + " (n=" + str(i[1]) + ")" \
               for i in zip(labels, [len(i[i != 0]) for i in scores_by_position])]

    for data, labelled, label in zip(scores_by_position, label_n, labels):
        ecdf = ECDF(data[data != 0])
        plt.plot(ecdf.x, ecdf.y, label=labelled, c=C_TLOC[label], lw=2)
    plt.legend(loc=0)
    plt.xlabel("ORF start " + xlabel)
    plt.xlim(-18, 0)
    plt.ylabel("Cumulative distribution")
```

**Supp Fig 2e-f**: Secondary structure EFEs at -25 (left) and +1 (right) w.r.t. ORF starts, subdivided by position in a transcript, visualized as a CDF plot.

In [37]:

```
for score, xlabel, u_data in zip(("sec_struct_EFE_L", "sec_struct_EFE_R"),
                                 ("(-25 from CDS start)", "(+1 from CDS start)"),
                                 (uORF_SSEFE_L_by_uORF_num, uORF_SSEFE_L_by_uORF_num)):
    plt.figure()
    labelleds = ("no uORFs", "1 uORF", "2 uORFs", "3 uORFs", "4+ uORFs")
    CDS_lines = {}
    uORF_lines = {}

    plt.subplot(2, 1, 1)
    for data, labelled, color in zip(u_data, labelleds[1:], C_UORF):
        ecdf = ECDF(data[data != 0])
        uORF_lines[labelled], = plt.plot(ecdf.x, ecdf.y, c=color, lw=2,
                                     label=labelled + " (n=" + str(len(data[data != 0])) + ")")

    first_legend = plt.legend(handles=[uORF_lines[labelled] for labelled in labelleds[1:]],
                              title="uORFs", loc="upper left")
    ax = plt.gca().add_artist(first_legend)
    
    plt.ylabel("Cumulative distribution")
    plt.xlim(-18, 0)
    plt.tick_params(axis='x', labelbottom='off')


    plt.subplot(2, 1, 2)
    for by_uORFs, labelled, color in zip(num_uORFs, labelleds, C_CDS):
        ecdf = ECDF(by_uORFs["CDS_" + score][by_uORFs["CDS_" + score] != 0])
        CDS_lines[labelled], = plt.plot(ecdf.x, ecdf.y, lw=2,
                                        label=labelled + " (n=" + str(len(by_uORFs[by_uORFs["CDS_" + score] != 0])) + ")",
                                        c=color)
    
    plt.legend(handles=[CDS_lines[labelled] for labelled in labelleds],
                              title="CDSes", loc="upper left")
    
    plt.xlim(-18, 0)
    plt.xlabel("ORF start " + xlabel)
    plt.ylabel("Cumulative distribution")
```

**Supp Fig 2g**: Mean secondary structure EFEs over 5' leaders and CDSes, subdivided by number of uORFs in transcript, visualized as a CDF plot.

In [38]:

```
labelleds = ("no uORFs", "1 uORF", "2 uORFs", "3 uORFs", "4+ uORFs")
CDS_lines = {}
uORF_lines = {}

for data, labelled, color in zip(CDS_mean_ssefe_35_by_uORF_num, labelleds, C_CDS):
    ecdf = ECDF(data[data != 0])
    CDS_lines[labelled], = plt.plot(ecdf.x, ecdf.y, c=color, lw=2,
                                    label=labelled + " (n=" + str(len(data[data != 0])) + ")")

first_legend = plt.legend(handles=[CDS_lines[labelled] for labelled in labelleds],
                          title="CDSes", loc="center left")
ax = plt.gca().add_artist(first_legend)

for data, labelled, color in zip(UTR5_mean_ssefe_35_by_uORF_num, labelleds[1:], C_UORF):
    ecdf = ECDF(data[data != 0])
    uORF_lines[labelled], = plt.plot(ecdf.x, ecdf.y, c=color, lw=2,
                                     label=labelled + " (n=" + str(len(data)) + ")")

plt.legend(handles=[uORF_lines[labelled] for labelled in labelleds[1:]],
           title="5' leaders", loc="upper left")
plt.xlim(-15, -3)
plt.xlabel("Mean secondary structure EFE over entire ORF")
plt.ylabel("Cumulative distribution")
```

Out[38]:

```
<matplotlib.text.Text at 0x3e4a40f0>
```

### uORF-CDS distance¶

**Fig 1e:** Trinucleotide bias (rolling mean over 15 nucleotides) of start and stop codons in the 5' leader as a function of distance w.r.t. CDS start, for all 3 vertebrates.

Codon and nucleotide frequencies were determined for each position of the 5’ leader with respect to the CDS start. Tri-nucleotide bias was calculated to be the log ratio of observed codon frequency a given position against the expected codon frequency given the nucleotide frequencies at that same position.

While this bias is not a direct measure of depletion of tri-nucleotides, it is a worst a conservative underestimate of depletion, hence any detectable bias against a tri-nucleotide (i.e. scoring below 0) can be confidently considered as to be depletion.

In [39]:

```
for sp in species:
    pd.rolling_mean(uORF_start_trint[sp], 15).plot(c=C_SP_BAR[sp], lw=3, 
                                                   label=species_label[sp] + " ATGs")
    pd.rolling_mean(uORF_end_trint[sp], 15).plot(c=C_SP_BAR[sp], style="--", lw=3,
                                                 label=species_label[sp] + " stop\ntrinucleotides")
    
plt.plot((-150, 50), (0, 0), c="k")
plt.xlim(-150, 0)
plt.ylim(-2, 0.2)
plt.xlabel("Position w.r.t. CDS start")
plt.ylabel("Trinucleotide bias")
plt.legend(loc="lower left", ncol=3)
```

Out[39]:

```
<matplotlib.legend.Legend at 0x3e961518>
```

**Supp Fig 3a-c**: Trinucleotide biases (moving average over 15 nucleotides) comparing start codons (thick solid green line) with a selection of other codons (dashed lines) with large variations in bias, for all 3 vertebrates.

In [40]:

```
TRINTS.remove("ATG")
for sp in species:
    plt.figure()
    data = log2((trint_freq_sp[sp] / trint_pos_expected_freq_sp[sp]).T["ATG"])    
    rolling_mean(data, 15).plot(label="ATG", lw=3, c=C_STARTS[0])
    for trint in TRINTS:
        data = log2((trint_freq_sp[sp] / trint_pos_expected_freq_sp[sp]).T[trint])
        if max(data) - min(data) > 1.6:
            rolling_mean(data, 15).plot(label=trint, ls="--", lw=2)
    plt.ylim(-2.0, 0.5)
    plt.xlim(-150, 0)
    plt.xlabel("Position w.r.t. CDS start")
    plt.ylabel("Trinucleotide bias (rolling mean)")
    plt.legend(title="Trinucleotides with greatest variation in bias", loc="lower left", ncol=4)
    plt.title(species_label[sp])
```

**Supp Fig 3d-f**: Trinucleotide biases (rolling mean over 15 nucleotides) of start and stop codons in 3 different frames, for each vertebrate.

In [41]:

```
frames = {frame: range(-200 + i, -2, 3) for frame, i in zip((-1, -2, -3), (0, 1, 2))}

for sp in species:
    plt.figure()
    for frame, color, style, lw in zip((-1, -2, -3),
                                       C_STOPS,
                                       ["-", "--", "-."],
                                       (2, 3, 5)):
        label = "Frame " + str(frame) + " Stops"
        if frame == -3:
            label = "Frame " + str(frame) + " Stops (in frame with CDS)"
        rm = pd.rolling_mean(uORF_end_trint[sp][frames[frame]], 15)
        rm.plot(ls=style, lw=lw, c=color, label=label)
    
    for frame, color, style, lw in zip((-1, -2, -3),
                                       C_STARTS,
                                       ["-", "--", "-."],
                                       (2, 3, 5)):
        label = "Frame " + str(frame) + " ATGs"
        if frame == -3:
            label = "Frame " + str(frame) + " ATGs (in frame with CDS)"
        rm = pd.rolling_mean(uORF_start_trint[sp][frames[frame]], 15)
        rm.plot(ls=style, lw=lw, c=color, label=label)
    
    plt.scatter(uORF_start_trint[sp].index, uORF_start_trint[sp], c=C_STARTS[2], alpha=0.5)
    plt.scatter(uORF_end_trint[sp].index, uORF_end_trint[sp], c=C_STOPS[2], alpha=0.5)

    plt.legend(loc="lower left")
    plt.xlabel("Stop position w.r.t. CDS start")
    plt.ylabel("Trinucleotide bias")
    plt.xlim(-150, 0)
    plt.ylim(-2.5, 0.5)
```

**Supp Fig 4a-c**: Frequency of uORF ends with respect to CDS start, in observed (dark magenta) and shuffled (light magenta) 5' leaders, for all 3 vertebrates. Moving averages over 15 nucleotides are plotted alongside scatterpoints of individual positions.

In [42]:

```
for sp, ymax in zip(species, (120, 120, 140)):
    plt.figure()
    
    rolling_mean(uORF_end_freq[sp].uORF_ends_exp,
                 15, center=True).plot(c=C_STOPS[2], ls="--", lw=2,
                                       label="Expected uORF ends\nfrom shuffled 5'UTRs")
    rolling_mean(uORF_end_freq[sp].uORF_ends_obs,
                 15, center=True).plot(c=C_STOPS[0], ls="-", lw=3,
                                       label="Observed uORF ends")
    
    plt.scatter(uORF_end_freq[sp].uORF_ends_obs.index,
                uORF_end_freq[sp].uORF_ends_obs, c=C_STOPS[0], alpha=0.5)
    plt.scatter(uORF_end_freq[sp].uORF_ends_exp.index,
                uORF_end_freq[sp].uORF_ends_exp, c=C_STOPS[2], alpha=0.5)
    
    plt.xlim(-150, 50)
    plt.ylim(0, ymax)
    plt.xlabel("Position w.r.t. CDS start")
    plt.ylabel("Frequency")
    plt.legend(loc="upper left")
    plt.title(species_label[sp])
```

**Fig 1f, Supp Fig 4d-f**: Depletion of uORF ends (observed - expected number of uORF ends) for 3 vertebrates, plotted as a rolling mean over 24 nucleotide positions. Individual species trends have accompanying underlying scatter plot.

In [43]:

```
for sp in species:
    rolling_mean(uORF_end_freq[sp].dep, 24).plot(label=species_label[sp], c=C_SP_BAR[sp], lw=3)

plt.plot((-150, 0), (0, 0), c="k")
plt.xlim(-150, 0)
plt.ylim(-30, 5)
plt.xlabel("Position w.r.t. CDS start")
plt.ylabel("uORF end frequency relative to expected")
plt.legend(loc="lower left", ncol=3)

for sp in species:
    plt.figure()
    plt.scatter(uORF_end_freq[sp].index, uORF_end_freq[sp].dep, c=C_SP[sp], s=15, alpha=0.5)
    rolling_mean(uORF_end_freq[sp].dep, 24).plot(label=species_label[sp], c=C_SP_BAR[sp], lw=3)
    plt.plot((-150, 0), (0, 0), c="k")
    plt.xlim(-150, 0)
    plt.ylim(-30, 5)
    plt.xlabel("Position w.r.t. CDS start")
    plt.ylabel("uORF end frequency relative to expected")
```
