# Supplementary material for: Conservation of uORF repressiveness and sequence features in mouse, human and zebrafish
Source: Nat Commun. 2016 May 24;7:11663. doi: 10.1038/ncomms11663 (PMC4890304; doi:10.1038/ncomms11663)
Supplement: Supplementary Software — Conversions of iPython/Jupyter notebooks documenting all analysis for manuscript. Latest versions of iPython/Jupyer notebooks are available at https://github.com/chewgl/uORF_repressiveness_supplemental [file ncomms11663-s2.zip › Fig 2 - Prevalence and General Effects of uORFs - Zebrafish.html]

Fig 2 - Prevalence and General Effects of uORFs - Zebrafish


# Fig 2 - Prevalence and General Effects of uORFs¶

## Dataset¶

In [1]:

```
# s = "mm"
# stage = "mES"
# ASSEMBLY = "GRCm38_ens"

s = "dr"
stage = "Shield"
ASSEMBLY = "Zv9_ens"

# s = "hs"
# stage = "HeLa"
# ASSEMBLY = "GRCh37_ens"
```

## Imports and parameters¶

In [2]:

```
# FILTER PARAMETERS
UTR5_LENGTH_MIN = 25
UTR3_LENGTH_MIN = 25

FPKM_MIN = 0.1
CDS_LENGTH_MIN = 100
CDS_READS_MIN = 1

UORF_LENGTH_MIN = 20
UORF_FROM_TRANSCRIPT_START_MIN = UTR5_LENGTH_MIN
UORF_READS_MIN = 1

UTR5_READS_MIN = 1

ORF_END_TRIM = 10
```

In [3]:

```
# IMPORTS
import corebio
import weblogolib

import matplotlib as mpl
import matplotlib.pyplot as plt
import numpy as np
import pandas as pd
import seaborn.apionly as sns

from Bio import SeqIO
from IPython.display import display, HTML, Markdown, Image
from ast import literal_eval
from numpy import argmax, mean, log10, log2, inf, nan, array, float64, subtract, multiply, divide, sign
from pandas import Panel, DataFrame, Series
from pandas.stats.moments import rolling_mean
from scipy.stats import spearmanr, ks_2samp, pearsonr, linregress, zscore, kendalltau, kde
from scipy.stats.mstats import trimboth
from sklearn.linear_model import Ridge, RidgeCV
from statsmodels.distributions import ECDF
from statsmodels.nonparametric.smoothers_lowess import lowess

%matplotlib inline
```

In [4]:

```
pd.options.display.mpl_style = 'default'
pd.options.mode.chained_assignment = None
mpl.rcParams['figure.figsize'] = 8, 6
mpl.rcParams['axes.labelsize'] = 12
mpl.rcParams['axes.titlesize'] = 16
mpl.rcParams['axes.facecolor'] = "#fdfdfd"
mpl.rcParams['grid.alpha'] = 0.5
mpl.rcParams['legend.fontsize'] = 12
mpl.rcParams['font.family'] = 'sans-serif'
mpl.rcParams['figure.autolayout'] = True
mpl.rcParams['savefig.dpi'] = 50
```

In [5]:

```
C_NT = ("#00d700", "#df1f00", "#0226cc", "#ffb700")
C_UORF = ('#b30000', '#e34a33', '#fc8d59', '#fdbb84', '#fdd49e')
C_CDS = ("#105e47", "#157e5f", "#1b9e77", "#48b192", "#76c4ad")
C_RATIO = "#7570b3"
C_BG = ("#000000", "#525252", "#969696", "#d9d9d9")
C_SP = {"hs": "#97543a", "mm": "#546079", "dr": "#3d7463"}
C_SP_BAR = {"hs": "#fc8d62", "mm": "#8da0cb", "dr": "#66c2a5"}
C_NOBIAS = "#ffd92f"
C_SCATTER = "#737373"
C_TREND = "#e41a1c"
C_TLOC = {"5' UTR": "#d95f02", "CDS start": "#1b9e77", "CDS internal": "#e7298a", "3' UTR": "#e6ab02"}
C_STARTS = ("#276419", "#4d9221", "#7fbc41")
C_STOPS = ("#8e0152", "#c51b7d", "#de77ae")
```

## Helper functions¶

In [6]:

```
def scatter_linear_trend(x, y, x_label, y_label, plot_trend=True, corr_table=True):
    data = DataFrame({"x": x, "y": y}).dropna()
    
    plt.figure()
    plt.scatter(data.x, data.y, alpha=0.25, c=C_SCATTER)

    mod = Ridge(normalize=False).fit(zip(*[data.x,]), data.y)
    x1, x2 = (np.percentile(data.x, 2.5), np.percentile(data.x, 97.5))
    y1, y2 = mod.predict([(x1,), (x2,)])
    
    plt.ylim(np.percentile(data.y, 1), np.percentile(data.y, 99))
    plt.xlabel(x_label)
    plt.ylabel(y_label)
    
    if plot_trend:
        plt.plot((x1, x2), (y1, y2), c=C_TREND, lw=2)

    if corr_table:
        to_display = DataFrame(columns=["Correlation\ncoefficient", "p-value"])

        corr, p = pearsonr(data.x, data.y)
        to_display.loc["Pearson linear"] = {"Correlation\ncoefficient": corr,
                                    "p-value": p}
        corr, p = spearmanr(data.x, data.y)
        to_display.loc["Spearman rank"] = {"Correlation\ncoefficient": corr,
                                  "p-value": p}

        display(Markdown(y_label + " against " + x_label))
        display(to_display.applymap('{:,.4g}'.format))
    
    return abs(y2 - y1), sign(y2 - y1)
```

In [7]:

```
def drop_inf(df):
    return df.replace([np.inf, -np.inf], np.nan).dropna()
```

## Reading in ORF characteristics data¶

In [8]:

```
DATA_DIR = "./data/" + s + "/"
ANNOTATIONS_DIR = "./annotations/"
```

In [9]:

```
CONVERTERS = {i:literal_eval for i in ("uORFs_reads", "uORFs_length", "uORFs_wrent_score", "uORFs_urent_score",
                                       "uORFs_wrent_seq", "uORFs_sec_struct_EFE_L", "uORFs_sec_struct_EFE_R",
                                       "uORFs_start_pos_wrt_tss", "uORFs_end_pos_wrt_CDS",
                                       "ORFs_wrent_score", "ORFs_urent_score", "ORFs_wrent_seq",
                                       "ORFs_sec_struct_EFE_L", "ORFs_sec_struct_EFE_R")}
```

In [10]:

```
species = ("hs", "mm", "dr")
species_label = {"hs": "Human", "mm": "Mouse", "dr": "Zebrafish"}
stage_species = {"HeLa": "hs", "mES": "mm", "Shield": "dr"}
species_stage = {"hs": "HeLa", "mm": "mES", "dr": "Shield"}
```

In [11]:

```
species_df = {sp: pd.read_csv("./data/" + sp + "/" + species_stage[sp] + "_main.df",
                              sep="\t", index_col=["Gene"], converters=CONVERTERS) for sp in species}
```

In [12]:

```
species_df_filtered = dict()
for i in species_df:
    species_df_filtered[i] = species_df[i][(species_df[i].UTR5_length >= UTR5_LENGTH_MIN) &
                                           (species_df[i].UTR3_length >= UTR3_LENGTH_MIN) &
                                           (species_df[i].Gene_Expression_FPKM >= FPKM_MIN) &
                                           (species_df[i].CDS_length >= CDS_LENGTH_MIN) &
                                           (species_df[i].CDS_reads >= CDS_READS_MIN) &
                                           (species_df[i].UTR5_reads_trunc >= UTR5_READS_MIN)]
```

Specific species dataset is made df\_main for subsequent analyses.

In [13]:

```
df_main = species_df[stage_species[stage]]
df_main_filtered = species_df_filtered[stage_species[stage]]
```

Translational efficiency (TE) of ORFs are calculated by dividing the number of reads in the ORF by their length and their gene expression. Ribosome profiling read densities over ORFs and entire 5' leaders are also calculated.

In [14]:

```
df_main["CDS_TE"] = df_main.CDS_reads \
                    / df_main.CDS_length.subtract(ORF_END_TRIM) \
                    / df_main.Gene_Expression_FPKM

df_main_filtered["CDS_density"] = df_main_filtered.CDS_reads / df_main_filtered.CDS_length.subtract(ORF_END_TRIM)

df_main_filtered["UTR5_density"] = df_main_filtered.UTR5_reads_trunc / df_main_filtered.UTR5_length.subtract(ORF_END_TRIM)

df_main_filtered["CDS_TE"] = df_main_filtered.CDS_density \
                             / df_main_filtered.Gene_Expression_FPKM

df_main_filtered["UTR5_TE"] = df_main_filtered.UTR5_density \
                              / df_main_filtered.Gene_Expression_FPKM

df_main_filtered["UTR5_uORF_density"] = df_main_filtered.UTR5_uORF_reads \
                                        / df_main_filtered.UTR5_uORF_tlength

df_main_filtered["UTR5_non_uORF_density"] = (df_main_filtered.UTR5_reads - df_main_filtered.UTR5_uORF_reads) \
                                            / (df_main_filtered.UTR5_length - df_main_filtered.UTR5_uORF_tlength)
    
df_main_filtered["UTR5_uORF_TE"] = df_main_filtered.UTR5_uORF_density \
                                   / df_main_filtered.Gene_Expression_FPKM

df_main_filtered["UTR5_non_uORF_TE"] = df_main_filtered.UTR5_non_uORF_density \
                                       / df_main_filtered.Gene_Expression_FPKM
```

## Defining various transcriptome subsets¶

To determine effect of the presense of uORFs, transcripts were grouped by how many uORF they had.

In [15]:

```
no_uORFs = df_main_filtered[df_main_filtered.num_uORFs == 0]
with_uORFs = df_main_filtered[df_main_filtered.num_uORFs > 0]

one_uORF = df_main_filtered[df_main_filtered.num_uORFs == 1]
two_uORF = df_main_filtered[df_main_filtered.num_uORFs == 2]
three_uORF = df_main_filtered[df_main_filtered.num_uORFs == 3]
more_uORF = df_main_filtered[df_main_filtered.num_uORFs >= 4]
```

**Supp Fig 13b**: Frequency histogram of uORF lengths for transcripts with one non-overlapping uORF.

In [16]:

```
# Creates set of 1 non-overlapping uORF
one_non_overlapping_uORF = df_main_filtered[(df_main_filtered.num_uORFs == 1)]   # 1 uORF

# Since set has only transcripts with 1 uORF, "singularize" some columns of data
for variable in ("uORFs_reads", "uORFs_length", "uORFs_wrent_score",
                 "uORFs_sec_struct_EFE_L", "uORFs_sec_struct_EFE_R", "uORFs_start_pos_wrt_tss", "uORFs_end_pos_wrt_CDS"):
    one_non_overlapping_uORF[variable] = one_non_overlapping_uORF[variable].apply(lambda x:x[0])

# Filter set for non-overlapping uORF, with uORFs of minimum length, reads and distance from transcript start
to_display = {}
to_display["    One uORF transcripts"] =  len(one_non_overlapping_uORF)

one_non_overlapping_uORF = one_non_overlapping_uORF[one_non_overlapping_uORF.uORFs_end_pos_wrt_CDS < 0]
to_display["   + non-overlapping"] = len(one_non_overlapping_uORF)

plt.bar(range(6, 159, 3), np.histogram(one_non_overlapping_uORF.uORFs_length, bins=range(6, 162, 3))[0], width=3)
plt.plot([21, 21], [0, 100], c="r")
plt.xlabel("uORF length (nucleotides)")
plt.ylabel("Frequency")
plt.title("Transcripts with one non-overlapping uORF")

one_non_overlapping_uORF = one_non_overlapping_uORF[one_non_overlapping_uORF.uORFs_length >= UORF_LENGTH_MIN]
to_display["  + min length (" + str(UORF_LENGTH_MIN) + ")"] = len(one_non_overlapping_uORF)

one_non_overlapping_uORF = one_non_overlapping_uORF[one_non_overlapping_uORF.uORFs_reads >= UORF_READS_MIN]
to_display[" + min reads (" + str(UORF_READS_MIN) + ")"] = len(one_non_overlapping_uORF)

one_non_overlapping_uORF = one_non_overlapping_uORF[one_non_overlapping_uORF.uORFs_start_pos_wrt_tss >= UORF_FROM_TRANSCRIPT_START_MIN]
to_display["+ min dist from TSS (" + str(UORF_FROM_TRANSCRIPT_START_MIN) + ")"] = len(one_non_overlapping_uORF)

# Calculate uORF TEs for set
one_non_overlapping_uORF["uORF_TE"] = one_non_overlapping_uORF.uORFs_reads / \
                                      one_non_overlapping_uORF.uORFs_length.subtract(ORF_END_TRIM) / \
                                      one_non_overlapping_uORF.Gene_Expression_FPKM
           
display(DataFrame(Series(to_display, name="n")))
```

|  | n |
| --- | --- |
| One uORF transcripts | 1236 |
| + non-overlapping | 1025 |
| + min length (20) | 612 |
| + min reads (1) | 405 |
| + min dist from TSS (25) | 257 |

```
c:\Anaconda2\lib\site-packages\matplotlib\figure.py:1653: UserWarning: This figure includes Axes that are not compatible with tight_layout, so its results might be incorrect.
  warnings.warn("This figure includes Axes that are not "
```

Creates analogous set of transcripts with two non-overlapping uORFs as a control.

In [17]:

```
# Creates set of 2 non-overlapping uORFs
two_non_overlapping_uORFs = df_main_filtered[(df_main_filtered.num_uORFs == 2)]

# uORFs not overlapping in frame
two_non_overlapping_uORFs = two_non_overlapping_uORFs[[i[0] != i[1] for i in two_non_overlapping_uORFs.uORFs_end_pos_wrt_CDS]]

# uORFs not overlapping
two_non_overlapping_uORFs = two_non_overlapping_uORFs[[i[0] + j[0] < i[1] \
                                                      for i, j in zip(two_non_overlapping_uORFs.uORFs_start_pos_wrt_tss,
                                                                      two_non_overlapping_uORFs.uORFs_length)]]

# uORFs minimum distance from transcript start, minimum length
two_non_overlapping_uORFs = two_non_overlapping_uORFs[[i[0] > UORF_LENGTH_MIN \
                                                       and i[1] > UORF_LENGTH_MIN \
                                                       for i in two_non_overlapping_uORFs.uORFs_length]]

display(Markdown("Number of two non-overlapping uORF Transcripts = %d" % len(two_non_overlapping_uORFs)))

# Calculate uORF TEs for set
for uORF in range(2):
    two_non_overlapping_uORFs["uORF_TE" + str(uORF)] = two_non_overlapping_uORFs.uORFs_reads.apply(lambda x:float(x[uORF])) / \
                                                       two_non_overlapping_uORFs.uORFs_length.apply(lambda x:x[uORF]).subtract(ORF_END_TRIM) / \
                                                       two_non_overlapping_uORFs.Gene_Expression_FPKM
```

Number of two non-overlapping uORF Transcripts = 171

## Reading in profile data¶

df\_profiles are the metagene distribution of ribosome profiling reads around the starts and stops of CDSes (as a quality control measure, showing some reads over 5' UTRs, over CDSes in triplet phasing, and few reads in 3' UTRs) for different species.

In [18]:

```
df_profiles = {sp: pd.read_csv("./data/" + sp + "/" + species_stage[sp] + "_profile.df",
                               index_col=0, sep="\t") for sp in species}
```

## Figures¶

### Number of uORFs per transcript¶

**Supp Fig 13a**: Distribution of number of uORFs per transcript for various species.

In [19]:

```
data = pd.DataFrame({species_label[i]:np.histogram(species_df_filtered[i].num_uORFs, bins=range(0, 10), normed=True)[0] \
                     for i in species})
    
data.plot(kind="bar", color=[C_SP_BAR[i] for i in species], width=0.8)
plt.xlabel("Number of uORFs")
plt.ylabel("Fraction of filtered transcripts")
plt.ylim(0, 0.5)
plt.legend()
```

Out[19]:

```
<matplotlib.legend.Legend at 0x1bbad470>
```

Some numbers describing the number of transcripts analyzed.

uORFs are defined purely by sequence: a transcript segment beginning before the annotated CDS with an ATG start and an in-frame stop codon.

"Translated uORF" here means that the number of reads is at least the minimum number of uORF reads as defined by UORF\_READS\_MIN.

In [20]:

```
to_display = DataFrame(columns=["Total", "Total with uORFs", "Total with translated uORFs",
                                "Filtered", "Filtered with uORFs", "Filtered with translated uORFs"])

for sp in species:
    to_display.loc[species_label[sp]] = \
    {"Total": len(species_df[sp]),
     "Total with uORFs": len([max(i) for i in species_df[sp].uORFs_reads.values if len(i) > 0]),
     "Total with translated uORFs": len([i for i in [max(i) for i in species_df[sp].uORFs_reads.values \
                                                     if len(i) > 0] if i != 0]),
     "Filtered": len(species_df_filtered[sp]),
     "Filtered with uORFs": len([max(i) for i in species_df_filtered[sp].uORFs_reads.values if len(i) > 0]),
     "Filtered with translated uORFs": len([i for i in [max(i) for i in species_df_filtered[sp].uORFs_reads.values \
                                                        if len(i) > 0] if i != 0])}
display(to_display.T)
```

|  | Human | Mouse | Zebrafish |
| --- | --- | --- | --- |
| Total | 29531 | 23756 | 20015 |
| Total with uORFs | 7887 | 8167 | 7521 |
| Total with translated uORFs | 5609 | 5580 | 4322 |
| Filtered | 9271 | 10109 | 5541 |
| Filtered with uORFs | 5445 | 5691 | 4397 |
| Filtered with translated uORFs | 4721 | 4795 | 3307 |

### Ribosome profiling data analyzed at nucleotide resolution¶

**Supp Fig 13c-e**: Metagene profiles of P-site reduced ribosome profiling reads around CDS start and ends for various species.

Cycloheximide pretreatment during ribosome profiling can introduce biases to ribosome profiling data (Ingolia et al. 2011), which are clearly seen when comparing cycloheximide-pretreated HeLa cell data with mESC and zebrafish data. Specifically, cycloheximide pretreatment causes broad accumulation (approximately 2-fold) of ribosomes at the beginning of ORFs, an effect extending approximately 40nt into the ORF in HeLa cell data. Similarly, the lack of cycloheximide pretreatment also results in a strong accumulation of ribosomes at the end of ORFs (approximately 4-fold) in mES and zebrafish embryo data.

We account for the latter by ignoring the reads over the last 10 nucleotides of ORFs. The effect of this accumulation near the beginning of ORFs in cycloheximide-pretreated samples can be seen in the HeLa cell data [Supp Fig 6]: uORF TE is artifically higher than CDS TE (due to uORFs being much shorter than CDSes); however, because this affects all uORFs within a sample, we can still determine how various uORF sequence features affect uORF TE, as well as the ratio of uORF vs CDS TE.

In [21]:

```
for i in species:
    ylim = max(max(df_profiles[i]["CDS_norm_start_reads"].dropna()),
               max(df_profiles[i]["CDS_norm_end_reads"].dropna())) * 1.05
    plt.figure()
    plt.subplot(121)
    plt.bar(df_profiles[i].index, df_profiles[i]["CDS_norm_start_reads"],
            color=C_BG[1], width=1)
    plt.xlim(-50, 50)
    plt.ylim(0, ylim)
    plt.ylabel("Normalized RP read density")
    plt.xlabel("Position w.r.t. CDS start")
    plt.title(species_label[i])
    
    ax = plt.subplot(122)
    plt.bar(df_profiles[i].index, df_profiles[i]["CDS_norm_end_reads"],
            color=C_BG[1], label=species_stage[i], width=1)
    plt.xlim(-50, 50)
    plt.ylim(0, ylim)
    plt.xlabel("Position w.r.t. CDS end")
    ax.set_yticklabels(())
```

### Effect of uORF presence on translational efficiency¶

**Fig 2a-b, Supp Fig 5a-b**: Distributions of CDS TE, CDS ribosome profiling read density, 5' leader TE and gene expression as a function of number of uORFs in a transcript, visualized as CDF plots.

In [22]:

```
for parameter, para_label, limits in zip(("CDS_TE", "CDS_density", "UTR5_TE", "Gene_Expression_FPKM"),
                                         ("CDS TE", "CDS RP read density",
                                          "5' leader TE", "gene expression (FPKM)"),
                                         [(-3, -0), (-3, 2), (-4, -0.5), (-1, 3.5)]):
    plt.figure()
    for data, labelled, color in zip((no_uORFs, one_uORF, two_uORF, three_uORF, more_uORF),
                                  ("No uORFs", "1 uORF", "2 uORFs", "3 uORFs", "4+ uORFs"),
                                  [C_SCATTER,] + list(C_UORF[:-1])):
        ecdf = ECDF(log10(data[parameter]))
        plt.plot(ecdf.x, ecdf.y,
                 label=labelled + " (n=" + str(len(data)) + ")",
                 c=color, lw=2)

    ecdf = ECDF(log10(with_uORFs[parameter]))
    plt.plot(ecdf.x, ecdf.y, "--",
             label="with uORFs" + " (n=" + str(len(ecdf.x)) + ")",
             c=C_UORF[0], lw=2)

    plt.xlim(*limits)
    plt.legend(loc=4)
    plt.xlabel("log10 " + para_label)
    plt.ylabel("Cumulative distribution")
    plt.title("All filtered transcripts")
    
    mean_trimmed_log_no_uORFs = mean(trimboth(log10(no_uORFs[parameter]), proportiontocut=0.05))

    to_display = DataFrame(columns=["No uORFs vs", "Mean diff (%)", "KS-test D", "KS-test p-value"])
    to_display = to_display.set_index(["No uORFs vs"])
    for data, labelled in zip((one_uORF, two_uORF, three_uORF, more_uORF, with_uORFs),
                              ("1 uORF", "2 uORFs", "3 uORFs", "4+ uORFs", "with uORFs")):

        mean_trimmed_log_i = mean(trimboth(log10(data[parameter]), proportiontocut=0.05))
        fold_change = 10 ** (mean_trimmed_log_i - mean_trimmed_log_no_uORFs)
        pct_change = (fold_change - 1) * 100
        d, p = ks_2samp(no_uORFs[parameter], data[parameter])

        to_display.loc[labelled] = {"Mean diff (%)": pct_change,
                                    "KS-test D": d, "KS-test p-value": p}
    #display(to_display.applymap('{:,.4g}'.format))
    
    # this is another inset axes over the main axes

    plt.figure(figsize=(5, 0.75 + float(len(to_display)) * .3))
    ax = to_display["Mean diff (%)"].iloc[::-1].plot(kind="barh", width=0.9,
                                                     color=list([C_UORF[0],] + list(C_UORF[-2::-1])))
    plt.xlabel("Difference (%)")
    #plt.xlim(-55, 0)
#     for i, label in enumerate(list(to_display.index[::-1])):
#         score = to_display.ix[label]["KS-test p-value"]
#         pct = to_display.ix[label]["Mean diff (%)"]
#         if pct < 0:
#             ax.annotate('p={:.3g}'.format(score), (-1, i), color="white",
#                         horizontalalignment='right', verticalalignment='center')
#         else:
#             ax.annotate('p={:.3g}'.format(score), (1, i), color="white",
#                         horizontalalignment='left', verticalalignment='center')
```

### Effect of uORF translation on downstream translational efficiency¶

**Fig 2c**: Scatter plots of various pairs of the following parameters for all filtered transcripts: CDS and 5' leader TEs, CDS and 5' leader ribosome profiling read density, and gene expression. Individual points and contours are colored by the number of uORFs within the transcript; the contours help visualize the distribution of these points.

In [23]:

```
def scatter_by_uORF_num(dataset, dataset_label, para_x, para_y, para_x_label, para_y_label, x_limits, y_limits):
    plt.figure()
    data = dataset[[para_x, para_y]].applymap(log10)
    data["num_uORF"] = dataset.num_uORFs.apply(lambda x: min(x, 4))
    data = drop_inf(data)

    cmap = mpl.colors.ListedColormap([C_SCATTER,] + list(C_UORF[:-1]))

    fig, ax = plt.subplots()
    fig1 = ax.scatter(data[para_x], data[para_y], c=data.num_uORF, s=15,
                      cmap=cmap, vmax=data.num_uORF.max(), alpha=0.3)

    for num_uORF, color in zip([0, 1, 2, 3, 4], [C_SCATTER,] + list(C_UORF[:-1])):
        sns.kdeplot(data[para_x][data.num_uORF == num_uORF], data[para_y][data.num_uORF == num_uORF],
                    n_levels=5, cmap=None, colors=color, linewidths=[0,3,0,0,0], alpha=1)

    cbar = fig.colorbar(fig1, ticks=[np.arange(0.4, 5, 0.8)])
    cbar.ax.set_yticklabels(["no uORFs", "1 uORF", "2 uORFs", "3 uORFs", "4+ uORFs"])

    plt.xlabel("log10 " + para_x_label)
    plt.ylabel("log10 " + para_y_label)
    plt.legend(loc="upper left")
    plt.xlim(*x_limits)
    plt.ylim(*y_limits)
    plt.title(dataset_label)
    
    to_display = DataFrame(columns=["Correlation\ncoefficient", "p-value"])

    corr, p = pearsonr(data[para_x], data[para_y])
    to_display.loc["Linear"] = {"Correlation\ncoefficient": corr,
                                "p-value": p}
    corr, p = spearmanr(data[para_x], data[para_y])
    to_display.loc["Rank"] = {"Correlation\ncoefficient": corr,
                              "p-value": p}

    display(Markdown(para_y_label + " against " + para_x_label))
    display(to_display.applymap('{:,.4g}'.format))
```

In [24]:

```
parameters = DataFrame({"CDS_TE": {"label": "CDS TE", "limits": (-3, -1)},
                        "UTR5_TE": {"label": "5' leader TE", "limits": (-4, -1)},
                        "CDS_density": {"label": "CDS RP read density", "limits": (-2.5, 1.5)},
                        "UTR5_density": {"label": "5' leader RP read density", "limits": (-2.5, 0.5)},
                        "Gene_Expression_FPKM": {"label": "gene expression (FPKM)", "limits": (-0.5, 3)},
                        "UTR5_length": {"label": "5' leader length", "limits": (1.4, 3)}})
```

In [25]:

```
dataset, dataset_label = df_main_filtered, "All filtered transcripts"

comparisons = [("CDS_TE", "CDS_density"), ("CDS_TE", "UTR5_TE"), ("CDS_density", "UTR5_density"),
               ("Gene_Expression_FPKM", "CDS_TE"), ("Gene_Expression_FPKM", "UTR5_TE"),
               ("Gene_Expression_FPKM", "CDS_density"), ("Gene_Expression_FPKM", "UTR5_density"),
               ("UTR5_length", "CDS_TE")]

for x, y in comparisons:
    scatter_by_uORF_num(dataset, dataset_label, x, y,
                        parameters[x].label, parameters[y].label,
                        parameters[x].limits, parameters[y].limits)
```

```
c:\Anaconda2\lib\site-packages\matplotlib\collections.py:650: FutureWarning: elementwise comparison failed; returning scalar instead, but in the future will perform elementwise comparison
  if self._edgecolors_original != str('face'):
c:\Anaconda2\lib\site-packages\matplotlib\axes\_axes.py:475: UserWarning: No labelled objects found. Use label='...' kwarg on individual plots.
  warnings.warn("No labelled objects found. "
```

CDS RP read density against CDS TE

|  | Correlation coefficient | p-value |
| --- | --- | --- |
| Linear | 0.4355 | 3.13e-255 |
| Rank | 0.4198 | 1.609e-235 |

5' leader TE against CDS TE

|  | Correlation coefficient | p-value |
| --- | --- | --- |
| Linear | 0.2281 | 2.429e-66 |
| Rank | 0.1608 | 2.073e-33 |

5' leader RP read density against CDS RP read density

|  | Correlation coefficient | p-value |
| --- | --- | --- |
| Linear | 0.5945 | 0 |
| Rank | 0.5868 | 0 |

CDS TE against gene expression (FPKM)

|  | Correlation coefficient | p-value |
| --- | --- | --- |
| Linear | -0.06129 | 4.983e-06 |
| Rank | -0.0005858 | 0.9652 |

5' leader TE against gene expression (FPKM)

|  | Correlation coefficient | p-value |
| --- | --- | --- |
| Linear | -0.5392 | 0 |
| Rank | -0.4852 | 0 |

CDS RP read density against gene expression (FPKM)

|  | Correlation coefficient | p-value |
| --- | --- | --- |
| Linear | 0.8718 | 0 |
| Rank | 0.8821 | 0 |

5' leader RP read density against gene expression (FPKM)

|  | Correlation coefficient | p-value |
| --- | --- | --- |
| Linear | 0.5752 | 0 |
| Rank | 0.5828 | 0 |

CDS TE against 5' leader length

|  | Correlation coefficient | p-value |
| --- | --- | --- |
| Linear | -0.1137 | 2.039e-17 |
| Rank | -0.08649 | 1.128e-10 |

```
<matplotlib.figure.Figure at 0x4c6ff940>
```

```
c:\Anaconda2\lib\site-packages\matplotlib\collections.py:590: FutureWarning: elementwise comparison failed; returning scalar instead, but in the future will perform elementwise comparison
  if self._edgecolors == str('face'):
```

```
<matplotlib.figure.Figure at 0x51265e80>
```

```
<matplotlib.figure.Figure at 0x516881d0>
```

```
<matplotlib.figure.Figure at 0x50704a90>
```

```
<matplotlib.figure.Figure at 0x5093b400>
```

```
<matplotlib.figure.Figure at 0x4f50b668>
```

```
<matplotlib.figure.Figure at 0x4eceb978>
```

```
<matplotlib.figure.Figure at 0x4e9d3f60>
```

Scatter plot of 5' leader TE as restricted to regions within uORFs, against CDS TE, for filtered transcripts with uORFs. Like above, individual points and contours are colored by the number of uORFs within a transcript.

In [26]:

```
data = df_main_filtered[["CDS_TE", "UTR5_uORF_TE"]].applymap(log10)
data["num_uORF"] = df_main_filtered.num_uORFs.apply(lambda x: min(x, 4))
data = drop_inf(data)

cmap = mpl.colors.ListedColormap(C_UORF[:-1])

fig, ax = plt.subplots()

fig1 = ax.scatter(data.CDS_TE, data.UTR5_uORF_TE, c=data.num_uORF, s=15,
                  cmap=cmap, vmax=data.num_uORF.max(), alpha=0.3)

for num_uORF, color in zip([1, 2, 3, 4], C_UORF[:-1]):
    sns.kdeplot(data.CDS_TE[data.num_uORF == num_uORF], data.UTR5_uORF_TE[data.num_uORF == num_uORF],
                n_levels=5, cmap=None, colors=color, linewidths=[0,3,0,0,0], alpha=1)

cbar = fig.colorbar(fig1, ticks=[np.arange(0.66, 5, 0.75)])
cbar.ax.set_yticklabels(["1 uORF", "2 uORFs", "3 uORFs", "4+ uORFs"])

plt.xlabel("log10 CDS TE")
plt.ylabel("log10 5' Leader uORF TE")
plt.legend(loc="upper left")
plt.xlim(-3, -1)
plt.ylim(-3, -0.5)
plt.title("Filtered transcripts with uORFs")
```

Out[26]:

```
<matplotlib.text.Text at 0x4fdd0f98>
```

**Fig 2d**: Scatter plot of uORF TE against cognate CDS TE, for transcripts with one non-overlapping uORF. Red line indicates linear (ridge) regression line.

In [27]:

```
logx, logy = [log10(one_non_overlapping_uORF[i]) for i in ("CDS_TE", "uORF_TE")]
scatter_linear_trend(logx, logy, "log10 CDS TE", "log10 uORF TE")
plt.xlim(-2.4, -0.6)
plt.ylim(-3, -1)
plt.title("Filtered transcripts with one non-overlapping uORF")
```

log10 uORF TE against log10 CDS TE

|  | Correlation coefficient | p-value |
| --- | --- | --- |
| Pearson linear | 0.3729 | 6.726e-10 |
| Spearman rank | 0.3346 | 3.864e-08 |

Out[27]:

```
<matplotlib.text.Text at 0x4ebb43c8>
```

**Supp Fig 5c**: Distributions of uORF and CDS TEs plotted as cumulative distribution

In [28]:

```
for i, labelled, color in zip((logx, logy),
                              ("CDS TE", "uORF TE"),
                              (C_CDS[2], C_UORF[1])):
    ecdf = ECDF(i)
    plt.plot(ecdf.x, ecdf.y,
             label=labelled + " (n=" + str(len(ecdf.x)) + ")",
             color=color, lw=2)

ecdf_CDS = ECDF(logx)
ecdf_uORF = ECDF(logy)
CDS_TE_med = [x for x, y in zip(ecdf_CDS.x, ecdf_CDS.y) if y < 0.5][-1]
uORF_TE_med = [x for x, y in zip(ecdf_uORF.x, ecdf_uORF.y) if y < 0.5][-1]
#uORF_CDS_med_val = [y for x, y in zip(ecdf_uORF.x, ecdf_uORF.y) if x > CDS_start_med][0]
plt.plot((CDS_TE_med, CDS_TE_med), (0, 0.5), "--", c="grey")
plt.plot((-4.5, CDS_TE_med), (0.5, 0.5), "--", c="grey")
plt.plot((uORF_TE_med, uORF_TE_med), (0, 0.5), "--", c="grey")
#plt.plot((-7, CDS_start_med), (uORF_CDS_med_val, uORF_CDS_med_val), "--", c="grey")    
    
plt.ylabel("Cumulative distribution")
plt.xlabel("log10 TE")
plt.legend(loc=0)
plt.xlim(-4.5, -0.5)
plt.title("Filtered transcripts with one non-overlapping uORF")
print uORF_TE_med, CDS_TE_med, 10 ** (CDS_TE_med - uORF_TE_med)
```

```
-2.09270033454 -1.67841555519 2.59588099779
```

And plotted as KDE.

In [29]:

```
for i, labelled, color in zip((logx, logy),
                              ("CDS TE", "uORF TE"),
                              (C_CDS[2], C_UORF[1])):
    i.plot(kind="kde", label=labelled, color=color, lw=2)
plt.xlabel("log10 TE")
plt.legend(loc=0)
plt.xlim(-4.5, 0)
plt.title("Filtered transcripts with one non-overlapping uORF")
```

Out[29]:

```
<matplotlib.text.Text at 0x4eb24a58>
```

**Supp Fig 5d**: Scatter plot of cognate uORF TEs for transcripts with two non-overlapping uORFs.

In [30]:

```
data = two_non_overlapping_uORFs[["uORF_TE0", "uORF_TE1"]]
data = data.applymap(log10).replace(-inf, nan).dropna()
scatter_linear_trend(data.uORF_TE0, data.uORF_TE1, "log10 uORF1 TE", "log10 uORF2 TE")
plt.xlim(-4, -1)
plt.ylim(-3, -1)
plt.title("Filtered transcripts with two non-overlapping uORFs")
```

log10 uORF2 TE against log10 uORF1 TE

|  | Correlation coefficient | p-value |
| --- | --- | --- |
| Pearson linear | 0.451 | 0.0001005 |
| Spearman rank | 0.4051 | 0.0005552 |

Out[30]:

```
<matplotlib.text.Text at 0x4de728d0>
```

Distributions of uORF 1 and uORF 2 TEs.

In [31]:

```
for i, labelled, color in zip((data.uORF_TE0, data.uORF_TE1),
                              ("uORF 1", "uORF 2"),
                              (C_UORF[0], C_UORF[3])):
    ecdf = ECDF(i)
    plt.plot(ecdf.x, ecdf.y,
             label=labelled + " (n=" + str(len(ecdf.x)) + ")",
             color=color, lw=2)
plt.xlabel("log10 uORF TE")
plt.ylabel("Cumulative distribution")
plt.legend(loc=0)
plt.xlim(-4, 0)
plt.title("Filtered transcripts with two non-overlapping uORFs")
```

Out[31]:

```
<matplotlib.text.Text at 0x4ec78c50>
```

### Determining "noise" of ribosome profiling data over 5' leaders¶

By comparing reads within vs without uORFs.

Scatter plots of uORF against non-uORF ribosome profiling read density, as well as uORF against 5' leader ribosome profiling read density, with linear (ridge) regression line indicated as a red line and the mean of both datasets indicated as a red point.

In [32]:

```
log_data = drop_inf(with_uORFs[["UTR5_uORF_density", "UTR5_non_uORF_density",
                                "UTR5_density", "CDS_density"]].applymap(log10))
scatter_linear_trend(log_data.UTR5_non_uORF_density, log_data.UTR5_uORF_density,
                     "log10 5' leader non-uORF RP read density",
                     "log10 5' leader uORF RP read density")

plt.scatter((mean(log_data.UTR5_non_uORF_density),), (mean(log_data.UTR5_uORF_density),), s=40, c='red')
plt.xlim(-2.5, 0.5)
plt.ylim(-2, 1)
plt.title("Filtered transcripts with uORFs")

scatter_linear_trend(log_data.UTR5_density, log_data.UTR5_uORF_density,
                     "log10 5' leader RP read density",
                     "log10 5' leader uORF RP read density")

plt.scatter((mean(log_data.UTR5_non_uORF_density),), (mean(log_data.UTR5_uORF_density),), s=40, c='red')
plt.xlim(-2, 1)
plt.ylim(-2, 1.5)
plt.title("Filtered transcripts with uORFs")
```

log10 5' leader uORF RP read density against log10 5' leader non-uORF RP read density

|  | Correlation coefficient | p-value |
| --- | --- | --- |
| Pearson linear | 0.4239 | 1.117e-109 |
| Spearman rank | 0.423 | 3.717e-109 |

log10 5' leader uORF RP read density against log10 5' leader RP read density

|  | Correlation coefficient | p-value |
| --- | --- | --- |
| Pearson linear | 0.8625 | 0 |
| Spearman rank | 0.8596 | 0 |

Out[32]:

```
<matplotlib.text.Text at 0x4dd27438>
```

Calculation of arithmetic and geometric means of the above ribosome profiling read densities, as well as some resultant ratios. uORF-regions are 3-4 times as dense for ribosome profiling reads as non-uORF regions within the 5' leader, while CDSes are 1.5-2 times as dense as uORF regions.

In [33]:

```
to_display = {"Geometric mean": {"5' leader uORF": 10**mean(log_data.UTR5_uORF_density),
                                 "5' leader non-uORF": 10**mean(log_data.UTR5_non_uORF_density),
                                 "CDS": 10**mean(log_data.CDS_density),
                                 "5' leader": 10**mean(log_data.UTR5_density)},
              "Arithmetic mean (dropped zeroes)": {"5' leader uORF": mean(with_uORFs.UTR5_uORF_density),
                                                   "5' leader non-uORF": mean(with_uORFs.UTR5_non_uORF_density),
                                                   "CDS": mean(with_uORFs.CDS_density),
                                                   "5' leader": mean(with_uORFs.UTR5_density)}}

to_display = DataFrame(to_display)
to_display.index.name = "RP read density"
display(DataFrame(to_display))

to_display1 = DataFrame(columns=["Geometric mean", "Arithmetic mean (dropped zeroes)"])
to_display1.index.name = "Ratio"
to_display1.loc["non-uORF/uORF"] = to_display.T["5' leader non-uORF"] / to_display.T["5' leader uORF"]
to_display1.loc["5' leader/CDS"] = to_display.T["5' leader"] / to_display.T["CDS"]
to_display1.loc["uORF/CDS"] = to_display.T["5' leader uORF"] / to_display.T["CDS"]
display(DataFrame(to_display1))
```

|  | Arithmetic mean (dropped zeroes) | Geometric mean |
| --- | --- | --- |
| RP read density |  |  |
| 5' leader | 0.412196 | 0.209631 |
| 5' leader non-uORF | 0.150131 | 0.079274 |
| 5' leader uORF | 1.323709 | 0.429184 |
| CDS | 1.030690 | 0.407343 |

|  | Geometric mean | Arithmetic mean (dropped zeroes) |
| --- | --- | --- |
| Ratio |  |  |
| non-uORF/uORF | 0.184708 | 0.113417 |
| 5' leader/CDS | 0.514628 | 0.399923 |
| uORF/CDS | 1.053617 | 1.284294 |

Distributions of ribosome profiling read densities over 5' leader uORF and non-uORF regions, 5' leader, and CDS, plotted as a CDF plot.

In [34]:

```
labels = ["5' leader uORF", "5' leader non-uORF", "5' leader", "CDS"]
log_data.columns = labels
for label in labels:
    ecdf = ECDF(log_data[label])
    plt.plot(ecdf.x, ecdf.y,
             label=label + " (n=" + str(len(ecdf.x)) + ")", lw=2)
plt.xlim(-2.5, 1.5)
plt.xlabel("log10 RP read density")
plt.ylabel("Cumulative distribution")
plt.title("Filtered transcripts with uORFs")
plt.legend(loc=0)
```

Out[34]:

```
<matplotlib.legend.Legend at 0x4fbca828>
```
