# Supplementary material for: Conservation of uORF repressiveness and sequence features in mouse, human and zebrafish
Source: Nat Commun. 2016 May 24;7:11663. doi: 10.1038/ncomms11663 (PMC4890304; doi:10.1038/ncomms11663)
Supplement: Supplementary Software — Conversions of iPython/Jupyter notebooks documenting all analysis for manuscript. Latest versions of iPython/Jupyer notebooks are available at https://github.com/chewgl/uORF_repressiveness_supplemental [file ncomms11663-s2.zip › Fig 3 (I) - Quantifying and modelling the effects of sequence features on uORF repressiveness - Mouse.html]

Fig 3 (I) - Quantifying and modelling the effects of sequence features on uORF repressiveness - Mouse


# Fig 3 (I) - Quantifying and modelling the effects of sequence features on uORF repressiveness¶

## Dataset¶

In [1]:

```
s = "mm"
stage = "mES"
ASSEMBLY = "GRCm38_ens"

# s = "dr"
# stage = "Shield"
# ASSEMBLY = "Zv9_ens"

# s = "hs"
# stage = "HeLa"
# ASSEMBLY = "GRCh37_ens"
```

## Imports, parameters and functions¶

In [2]:

```
# FILTER PARAMETERS
UTR5_LENGTH_MIN = 25
UTR3_LENGTH_MIN = 25

FPKM_MIN = 0.1
CDS_LENGTH_MIN = 100
CDS_READS_MIN = 1

UORF_LENGTH_MIN = 20
UORF_FROM_TRANSCRIPT_START_MIN = UTR5_LENGTH_MIN
UORF_READS_MIN = 1

UTR5_READS_MIN = 1

ORF_END_TRIM = 10
```

In [3]:

```
# IMPORTS
import corebio
import weblogolib

import matplotlib as mpl
import matplotlib.pyplot as plt
import numpy as np
import pandas as pd
import seaborn.apionly as sns

from Bio import SeqIO
from IPython.display import display, HTML, Markdown, Image
from ast import literal_eval
from numpy import argmax, mean, log10, log2, inf, nan, array, float64, subtract, multiply, divide, sign
from pandas import Panel, DataFrame, Series
from pandas.stats.moments import rolling_mean
from scipy.stats import spearmanr, ks_2samp, pearsonr, linregress, zscore, kendalltau, kde, gamma
from scipy.stats.mstats import trimboth
from sklearn.linear_model import Ridge, RidgeCV
from statsmodels.distributions import ECDF
from statsmodels.nonparametric.smoothers_lowess import lowess

%matplotlib inline
```

In [4]:

```
pd.options.display.mpl_style = 'default'
pd.options.mode.chained_assignment = None
mpl.rcParams['figure.figsize'] = 8, 6
mpl.rcParams['axes.labelsize'] = 12
mpl.rcParams['axes.titlesize'] = 16
mpl.rcParams['axes.facecolor'] = "#fdfdfd"
mpl.rcParams['grid.alpha'] = 0.5
mpl.rcParams['legend.fontsize'] = 12
mpl.rcParams['font.family'] = 'sans-serif'
mpl.rcParams['figure.autolayout'] = True
mpl.rcParams['savefig.dpi'] = 50
```

In [5]:

```
C_NT = ("#00d700", "#df1f00", "#0226cc", "#ffb700")
C_UORF = ('#b30000', '#e34a33', '#fc8d59', '#fdbb84', '#fdd49e')
C_CDS = ("#105e47", "#157e5f", "#1b9e77", "#48b192", "#76c4ad")
C_RATIO = "#7570b3"
C_BG = ("#000000", "#525252", "#969696", "#d9d9d9")
C_SP = {"hs": "#97543a", "mm": "#546079", "dr": "#3d7463"}
C_SP_BAR = {"hs": "#fc8d62", "mm": "#8da0cb", "dr": "#66c2a5"}
C_NOBIAS = "#ffd92f"
C_SCATTER = "#737373"
C_TREND = "#e41a1c"
C_TLOC = {"5' UTR": "#d95f02", "CDS start": "#1b9e77", "CDS internal": "#e7298a", "3' UTR": "#e6ab02"}
C_STARTS = ("#276419", "#4d9221", "#7fbc41")
C_STOPS = ("#8e0152", "#c51b7d", "#de77ae")
```

In [6]:

```
def scatter_linear_trend(x, y, x_label, y_label, plot_trend=True, corr_table=True):
    data = DataFrame({"x": x, "y": y}).dropna()
    
    plt.figure()
    plt.scatter(data.x, data.y, alpha=0.25, c=C_SCATTER)

    mod = Ridge(normalize=False).fit(zip(*[data.x,]), data.y)
    x1, x2 = (np.percentile(data.x, 2.5), np.percentile(data.x, 97.5))
    y1, y2 = mod.predict([(x1,), (x2,)])
    
    plt.ylim(np.percentile(data.y, 1), np.percentile(data.y, 99))
    plt.xlabel(x_label)
    plt.ylabel(y_label)
    
    if plot_trend:
        plt.plot((x1, x2), (y1, y2), c=C_TREND, lw=2)

    if corr_table:
        to_display = DataFrame(columns=["Correlation\ncoefficient", "p-value"])

        corr, p = pearsonr(data.x, data.y)
        to_display.loc["Pearson linear"] = {"Correlation\ncoefficient": corr,
                                    "p-value": p}
        corr, p = spearmanr(data.x, data.y)
        to_display.loc["Spearman rank"] = {"Correlation\ncoefficient": corr,
                                  "p-value": p}

        display(Markdown(y_label + " against " + x_label))
        display(to_display.applymap('{:,.4g}'.format))
    
    return abs(y2 - y1), sign(y2 - y1)
```

In [7]:

```
def scatter_lowess(x, y, x_label, y_label, color=C_TREND, corr_table=False):
    '''
    scatter plots x and y (should be same size), labelled by x_label and y_label
    Determines linear correlation, plots LOWESS fit line
    Outputs linear and rank correlations, as well as p-values
    '''
    data = DataFrame({"x": x, "y": y}).dropna()
    
    plt.figure()
    plt.scatter(data.x, data.y, alpha=0.25, c=C_SCATTER)

    lowx, lowy = lowess(y, x, it=10).T 
    plt.plot(lowx, lowy, c=color, lw=2)

    plt.ylim(np.percentile(data.y, 1), np.percentile(data.y, 99))
    
    plt.xlabel(x_label)
    plt.ylabel(y_label)
    
    if corr_table:
        to_display = DataFrame(columns=["r/rho", "p"])

        corr, p = pearsonr(data.x, data.y)
        to_display.loc["Pearson (linear)"] = {"r/rho": corr, "p": p}
        corr, p = spearmanr(data.x, data.y)
        to_display.loc["Spearman (rank)"] = {"r/rho": corr, "p": p}

        display(Markdown(y_label + " against " + x_label))
        display(to_display.applymap('{:,.4g}'.format))
    
    mod = Ridge(normalize=False).fit(zip(*[data.x,]), data.y)
    x1, x2 = (np.percentile(data.x, 2.5), np.percentile(data.x, 97.5))
    y1, y2 = mod.predict([(x1,), (x2,)])
    return abs(y2 - y1), sign(y2 - y1)
```

In [8]:

```
def flatten(i):
    return [j for k in i for j in k]

def clean(i):
    return i.replace([-inf, inf], nan).dropna()

def z(i):
    return (i - i.mean()) / i.std(ddof=0)
```

In [9]:

```
def lowess_joint(data, x_parameter, x_label, y_parameters, y_labels, colors, limits):
    for y_parameter, y_label, color in zip(y_parameters, y_labels, colors):
        plt.plot(*lowess(log2(data[y_parameter]), data[x_parameter], it=10).T,
                 label=y_label, color=color, lw=2)

    plt.xlabel(x_label)
    plt.ylabel("log2 value")
    plt.xlim(*limits[0])
    plt.ylim(*limits[1])
    plt.legend(loc="lower right")
```

In [10]:

```
def lowess_split(data, x_parameter, x_label, y_parameters, y_labels, colors, limits):
    to_display = DataFrame(columns=["Pearson r", "p",
                                    "Fold change"])
    
    for y_parameter, y_label, color in zip(y_parameters, y_labels, colors):
        y_range, y_sign = scatter_lowess(data[x_parameter], log2(data[y_parameter]),
                                         x_label, "log2 " + y_label, color=color)
        plt.xlim(*limits[0])
        plt.ylim(*limits[1])
    
        corr, p = pearsonr(data[x_parameter], log2(data[y_parameter]))
        
        to_display.loc[y_label] = {"Pearson r": corr, "p": p,
                                   "Fold change": 2**y_range * y_sign}
    display(Markdown(x_label))
    display(to_display.applymap('{:,.4g}'.format))
```

In [11]:

```
def lowess_versus(data, x_parameters, x_labels, y_parameters, y_labels, colors, limits):
    for x_parameter, x_label, ls in zip(x_parameters, x_labels, ("--", "-")):
        for y_parameter, y_label, color in zip(y_parameters, y_labels, colors):
            plt.plot(*lowess(log2(data[y_parameter]), data[x_parameter], it=10).T,
                     label=y_label + x_label, color=color, ls=ls, lw=2)
    
    plt.xlabel(x_labels[2])
    plt.ylabel("log2 value")
    plt.xlim(*limits[0])
    plt.ylim(*limits[1])
    plt.legend(loc="lower right", ncol=2)
```

In [12]:

```
def lin_regress_sets_RidgeCV(regressor_sets, parameter_sets, data_set_name, data_set,
                             parameter_to_regress, to_regress_label, limits, limits_coeff):
    
    to_display1 = DataFrame(columns=["Pearson r", "p", "Fold change", "PRESS", "RESS", "n"])
    
    sets_to_regress = list(set(flatten(regressor_sets)))
    regressors = [i + "_Z" for i in flatten([parameters[i][0] for i in sets_to_regress])]
    data = clean(data_set[regressors + [parameter_to_regress]])
    
    for regressor_set in regressor_sets:
        
        regressors = [i + "_Z" for i in flatten([parameter_sets[i][0] for i in regressor_set])]
        regressor_labels = [i for i in flatten([parameter_sets[i][1] for i in regressor_set])]
        colors = flatten([[parameter_sets[i][3] for j in range(len(parameter_sets[i][0]))] for i in regressor_set])

        mod = RidgeCV(normalize=False, store_cv_values=True).fit(data[regressors], data[parameter_to_regress])
        combined_score = np.sum([data[i].multiply(j) for i, j in zip(regressors, mod.coef_)], axis=0)
        
        plt.figure()
        plt.scatter(combined_score, data[parameter_to_regress], alpha=0.25, c=C_SCATTER)
        plt.xlabel("+".join(regressor_set) + " combined score")
        plt.ylabel(to_regress_label)
        plt.xlim(*limits[0])
        plt.ylim(*limits[1])
        plt.title(data_set_name)

        corr, p = pearsonr(combined_score, data[parameter_to_regress])

        x1, x2 = [np.percentile(combined_score, i) for i in [2.5, 97.5]]
        predicted = mod.predict(data[regressors])
        y1, y2 = [np.percentile(predicted, i) for i in [2.5, 97.5]]
        plt.plot((x1, x2), (y1, y2), c=C_TREND, lw=2)
        
        to_display1.loc[" + ".join(regressor_set)] = {"Pearson r": corr, "p": p,
                                                      "Fold change": 2**(y2-y1),
                                                      "PRESS": sum(mod.cv_values_)[0],
                                                      "RESS": sum((predicted - data[parameter_to_regress]) ** 2),
                                                      "n": len(data[parameter_to_regress])}
        
        display(Markdown(" + ".join(regressor_set) + " sequence features"))
        to_display = DataFrame(columns=["Relative contribution", "per unit 2fold change"])
        for i, label, j in zip(regressors, regressor_labels, mod.coef_):
            to_display.loc[label] = {"Relative contribution": j,
                                     "per unit 2fold change": data_set[i[:-2]].std() * j}
        to_display.index.name = "Sequence feature"
        display(to_display.applymap('{:,.4g}'.format))
        
        plt.figure(figsize=(6, 0.75 + float(len(to_display)) * .3))
        ax = to_display["Relative contribution"].plot(kind="barh", width=0.9, color=colors)
        ax.set_xlabel("Relative contribution")
        plt.xlim(*limits_coeff)
        plt.title("uORF repressiveness")
        print
    to_display1.index.name = "Sequence feature set"
    display(to_display1.iloc[::-1].applymap('{:,.4g}'.format))
```

## Reading in ORF characteristics data¶

In [13]:

```
DATA_DIR = "./data/" + s + "/"
ANNOTATIONS_DIR = "./annotations/"
```

In [14]:

```
CONVERTERS = {i:literal_eval for i in ("uORFs_reads", "uORFs_length", "uORFs_wrent_score", "uORFs_urent_score",
                                       "uORFs_wrent_seq", "uORFs_sec_struct_EFE_L", "uORFs_sec_struct_EFE_R",
                                       "uORFs_start_pos_wrt_tss", "uORFs_end_pos_wrt_CDS",
                                       "ORFs_wrent_score", "ORFs_urent_score", "ORFs_wrent_seq",
                                       "ORFs_sec_struct_EFE_L", "ORFs_sec_struct_EFE_R")}
```

In [15]:

```
stage_species = {"mES": "mm", "HeLa": "hs", "Shield": "dr"}
mean_ssefes = pd.read_table(DATA_DIR + stage_species[stage] + "_mean_ssefes.df", sep="\t", index_col=[0])
```

In [16]:

```
windows = [25, 30, 35, 40]
df_main = pd.read_csv(DATA_DIR + stage + "_main.df", index_col="Transcript", sep="\t", converters=CONVERTERS)
for window in windows:
    for pos in ("UTR5", "CDS"):
        field = pos + "_mean_ssefe_" + str(window)
        df_main[field] = mean_ssefes[field][mean_ssefes.index.isin(df_main.index)]
        df_main[field + "_Z"] = z(df_main[field])
```

In [17]:

```
df_main_filtered = df_main[(df_main.UTR5_length >= UTR5_LENGTH_MIN) &
                           (df_main.UTR3_length >= UTR3_LENGTH_MIN) &
                           (df_main.Gene_Expression_FPKM >= FPKM_MIN) &
                           (df_main.CDS_length >= CDS_LENGTH_MIN) &
                           (df_main.CDS_reads >= CDS_READS_MIN) &
                           (df_main.UTR5_reads_trunc >= UTR5_READS_MIN)]
```

In [18]:

```
df_main_filtered["CDS_TE"] = df_main_filtered.CDS_reads / \
                             df_main_filtered.CDS_length.subtract(ORF_END_TRIM) / \
                             df_main_filtered.Gene_Expression_FPKM

df_main_filtered["log2_CDS_TE"] = log2(df_main_filtered.CDS_TE)

df_main_filtered["num_uORFs_density"] = df_main_filtered.num_uORFs.apply(float) / \
                                        df_main_filtered.UTR5_length

for parameter in ("CDS_TE", "CDS_length", "UTR5_length", "Gene_Expression_FPKM", "num_uORFs_density"):
    df_main_filtered["log_" + parameter] = clean(log10(df_main_filtered[parameter].apply(float)))
    df_main_filtered["log_" + parameter + "_Z"] = z(df_main_filtered["log_" + parameter])

for parameter in ("CDS_wrent_score", "CDS_sec_struct_EFE_L", "CDS_sec_struct_EFE_R",
                  "UTR5_GC", "CDS_GC", "num_uORFs"):
    df_main_filtered[parameter + "_Z"] = z(df_main_filtered[parameter])
```

## Restricting uORF sequence feature effect analyses to transcripts with only one non-overlapping uORF¶

In [19]:

```
no_uORFs = df_main_filtered[df_main_filtered.num_uORFs == 0]
with_uORFs = df_main_filtered[df_main_filtered.num_uORFs > 0]
```

In [20]:

```
# Creates set of 1 non-overlapping uORF
one_non_overlapping_uORF = df_main_filtered[(df_main_filtered.num_uORFs == 1)]   # 1 uORF

# Since set has only transcripts with 1 uORF, "singularize" some columns of data
for variable in ("uORFs_reads", "uORFs_length", "uORFs_wrent_score", "uORFs_urent_score",
                 "uORFs_sec_struct_EFE_L", "uORFs_sec_struct_EFE_R", "uORFs_start_pos_wrt_tss",
                 "uORFs_end_pos_wrt_CDS"):
    one_non_overlapping_uORF[variable] = one_non_overlapping_uORF[variable].apply(lambda x:x[0])

# Filter set for non-overlapping uORF, with uORFs of minimum length, reads and distance from transcript start
one_non_overlapping_uORF = one_non_overlapping_uORF[\
    (one_non_overlapping_uORF.uORFs_end_pos_wrt_CDS < 0) &
    (one_non_overlapping_uORF.uORFs_reads >= UORF_READS_MIN) &
    (one_non_overlapping_uORF.uORFs_length >= UORF_LENGTH_MIN) &
    (one_non_overlapping_uORF.uORFs_start_pos_wrt_tss >= UORF_FROM_TRANSCRIPT_START_MIN)]
print "Number of one non-overlapping uORF Transcripts = %d" % len(one_non_overlapping_uORF)

# Calculate uORF TEs for set
one_non_overlapping_uORF["uORF_TE"] = one_non_overlapping_uORF.apply(lambda x: float64(x.uORFs_reads) \
                                                                               / (x.uORFs_length - ORF_END_TRIM) \
                                                                               / x.Gene_Expression_FPKM, axis=1)
```

```
Number of one non-overlapping uORF Transcripts = 750
```

## Calculate uORF repressiveness¶

uORF-mediated translational repression (or uORF repressiveness) are measured by the log ratio of the density of ribosome profiling reads over uORFs and CDSes.

In [21]:

```
# uORF inhibition as measured by log ratio of ribosome profiling read densities for uORFs and CDSes
uORF_density = one_non_overlapping_uORF.apply(lambda x: float64(x.uORFs_reads) \
                                                        / (x.uORFs_length - ORF_END_TRIM), axis=1)
CDS_density = one_non_overlapping_uORF.CDS_reads / \
              one_non_overlapping_uORF.CDS_length.subtract(ORF_END_TRIM)

one_non_overlapping_uORF["ratio"] = uORF_density / CDS_density
one_non_overlapping_uORF["log_ratio"] = log2(uORF_density / CDS_density)
```

In [22]:

```
one_non_overlapping_uORF["uORF_CDS_log_dist"] = one_non_overlapping_uORF.uORFs_end_pos_wrt_CDS.apply(lambda x:log10(-x))

for parameter in ("uORFs_length", "uORFs_start_pos_wrt_tss"):
    one_non_overlapping_uORF["log_" + parameter] = log10(one_non_overlapping_uORF[parameter].apply(float))
    one_non_overlapping_uORF["log_" + parameter + "_Z"] = z(one_non_overlapping_uORF["log_" + parameter])

for parameter in ("uORFs_wrent_score", "uORFs_sec_struct_EFE_L", "uORFs_sec_struct_EFE_R", "uORF_CDS_log_dist"):
    one_non_overlapping_uORF[parameter + "_Z"] = z(one_non_overlapping_uORF[parameter])

for parameter in ("CDS_TE", "CDS_length", "UTR5_length", "Gene_Expression_FPKM", "num_uORFs_density"):
    one_non_overlapping_uORF["log_" + parameter + "_Z"] = z(one_non_overlapping_uORF["log_" + parameter])

for parameter in ("CDS_wrent_score", "CDS_sec_struct_EFE_L", "CDS_sec_struct_EFE_R", "UTR5_GC", "CDS_GC", "num_uORFs"):
    one_non_overlapping_uORF[parameter + "_Z"] = z(one_non_overlapping_uORF[parameter])
```

## Figures¶

In [23]:

```
data = one_non_overlapping_uORF

y_parameters = ("ratio", "CDS_TE", "uORF_TE")
y_labels = ("uORF TE / CDS TE", "CDS TE", "uORF TE")
colors = (C_RATIO, C_CDS[2], C_UORF[1])
limits = ((-12, 8), (-8, 0))
```

**Fig 3a**: Relationship of uORF WRENT score to uORF repressiveness, CDS TE and uORF TE; only the trends (determined using locally-weighted scatterplot smoothing, or LOWESS) are shown.

In [24]:

```
x_parameter, x_label = ("uORFs_wrent_score", "uORF WRENT score")
lowess_joint(one_non_overlapping_uORF, x_parameter, x_label, y_parameters, y_labels, colors, limits)
```

```
c:\Anaconda2\lib\site-packages\matplotlib\figure.py:1653: UserWarning: This figure includes Axes that are not compatible with tight_layout, so its results might be incorrect.
  warnings.warn("This figure includes Axes that are not "
```

**Supp Fig 1c**: Relationship of uORF unweighted and weighted relative entropy scores (URENT and WRENT) to uORF repressiveness, CDS TE and uORF TE; only the LOWESS trends are shown.

In [25]:

```
x_parameters = ["uORFs_urent_score", "uORFs_wrent_score"]
x_labels = [" (unweighted)", " (weighted)", "uORF relative entropy score"]

lowess_versus(data, x_parameters, x_labels, y_parameters, y_labels, colors, limits)
```

**Supp Fig 6a, d, g; Supp Table 1:** Relationship of uORF unweighted and weighted relative entropy scores (URENT and WRENT) to uORF repressiveness, CDS TE and uORF TE; LOWESS trends and underlying scatter plots are shown.

In [26]:

```
x_parameter, x_label = ("uORFs_urent_score", "uORF URENT score")
lowess_split(one_non_overlapping_uORF, x_parameter, x_label, y_parameters, y_labels, colors, limits)

x_parameter, x_label = ("uORFs_wrent_score", "uORF WRENT score")
lowess_split(one_non_overlapping_uORF, x_parameter, x_label, y_parameters, y_labels, colors, limits)
```

uORF URENT score

|  | Pearson r | p | Fold change |
| --- | --- | --- | --- |
| uORF TE / CDS TE | 0.1204 | 0.0009514 | 2.045 |
| CDS TE | -0.1477 | 4.902e-05 | -1.645 |
| uORF TE | 0.0362 | 0.3222 | 1.243 |

uORF WRENT score

|  | Pearson r | p | Fold change |
| --- | --- | --- | --- |
| uORF TE / CDS TE | 0.138 | 0.0001507 | 2.352 |
| CDS TE | -0.09225 | 0.01148 | -1.383 |
| uORF TE | 0.08458 | 0.02053 | 1.7 |

```
c:\Anaconda2\lib\site-packages\matplotlib\collections.py:590: FutureWarning: elementwise comparison failed; returning scalar instead, but in the future will perform elementwise comparison
  if self._edgecolors == str('face'):
```

**Supp Fig 1d**: Relationship of CDS unweighted and weighted relative entropy scores (URENT and WRENT) to uORF repressiveness, CDS TE and uORF TE; only the LOWESS trends are shown.

In [27]:

```
x_parameters = ["CDS_urent_score", "CDS_wrent_score"]
x_labels = [" (unweighted)", " (weighted)", "CDS relative entropy score"]
limits=((-8, 8), (-8, 0))

lowess_versus(data, x_parameters, x_labels, y_parameters, y_labels, colors, limits)
```

Relationship of CDS unweighted and weighted relative entropy scores (URENT and WRENT) to uORF repressiveness, CDS TE and uORF TE; LOWESS trends and underlying scatter plots are shown.

In [28]:

```
x_parameter, x_label = ("CDS_urent_score", "CDS URENT score")
lowess_split(one_non_overlapping_uORF, x_parameter, x_label, y_parameters, y_labels, colors, limits)

x_parameter, x_label = ("CDS_wrent_score", "CDS WRENT score")
lowess_split(one_non_overlapping_uORF, x_parameter, x_label, y_parameters, y_labels, colors, limits)
```

CDS URENT score

|  | Pearson r | p | Fold change |
| --- | --- | --- | --- |
| uORF TE / CDS TE | -0.1091 | 0.002765 | -1.88 |
| CDS TE | 0.08621 | 0.01821 | 1.327 |
| uORF TE | -0.0595 | 0.1035 | -1.417 |

CDS WRENT score

|  | Pearson r | p | Fold change |
| --- | --- | --- | --- |
| uORF TE / CDS TE | -0.107 | 0.003343 | -1.904 |
| CDS TE | 0.1522 | 2.859e-05 | 1.681 |
| uORF TE | -0.02045 | 0.5761 | -1.133 |

#### Comparing CDS and uORF WRENT scores¶

Generally no correlation seen between CDS and uORF WRENT scores, implying that observed effect of uORF WRENT scores on CDS translation is not a result of existing biases in the data.

In [29]:

```
scatter_lowess(one_non_overlapping_uORF["uORFs_wrent_score"],
               one_non_overlapping_uORF["CDS_wrent_score"],
               "uORF WRENT score", "CDS WRENT score", corr_table=True)
plt.xlim(-12, 8)
```

CDS WRENT score against uORF WRENT score

|  | r/rho | p |
| --- | --- | --- |
| Pearson (linear) | 0.04544 | 0.2138 |
| Spearman (rank) | 0.0434 | 0.2352 |

Out[29]:

```
(-12, 8)
```

**Fig 3b**: Relationship of uORF initiation context secondary structure EFE (right of uORF start) to uORF repressiveness, CDS TE, and uORF TE; only the trends (determined using locally-weighted scatterplot smoothing, or LOWESS) are shown.

In [30]:

```
data = one_non_overlapping_uORF
x_parameter, x_label = ("uORFs_sec_struct_EFE_R", "uORF start sec struct EFE (+1 nts from uORF start)")
limits = ((-25, 0), (-8, 0))
```

In [31]:

```
lowess_joint(one_non_overlapping_uORF, x_parameter, x_label, y_parameters, y_labels, colors, limits)
```

Relationship of uORF initiation context secondary structure EFE (left and right of uORF start) to uORF repressiveness, CDS TE, and uORF TE; only the trends (determined using locally-weighted scatterplot smoothing, or LOWESS) are shown.

In [32]:

```
x_parameters = ["uORFs_sec_struct_EFE_L", "uORFs_sec_struct_EFE_R"]
x_labels = [" (-25 nts)", " (+1 nts)", "uORF sec struct EFE (pos w.r.t. start)"]
lowess_versus(data, x_parameters, x_labels, y_parameters, y_labels, colors, limits)
```

**Supp Fig 6b, e, h; Supp Table 1**: Relationship of uORF initiation context secondary structure EFE (left and right of uORF start) to uORF repressiveness, CDS TE and uORF TE; LOWESS trends and underlying scatter plots are shown.

In [33]:

```
x_parameter, x_label = ("uORFs_sec_struct_EFE_L", "uORF sec struct EFE (-25 from start)")
lowess_split(one_non_overlapping_uORF, x_parameter, x_label, y_parameters, y_labels, colors, limits)

x_parameter, x_label = ("uORFs_sec_struct_EFE_R", "uORF sec struct EFE (+1 from start)")
lowess_split(one_non_overlapping_uORF, x_parameter, x_label, y_parameters, y_labels, colors, limits)
```

uORF sec struct EFE (-25 from start)

|  | Pearson r | p | Fold change |
| --- | --- | --- | --- |
| uORF TE / CDS TE | 0.09443 | 0.009665 | 1.754 |
| CDS TE | 0.1365 | 0.0001773 | 1.585 |
| uORF TE | 0.1698 | 2.935e-06 | 2.781 |

uORF sec struct EFE (+1 from start)

|  | Pearson r | p | Fold change |
| --- | --- | --- | --- |
| uORF TE / CDS TE | 0.1544 | 2.171e-05 | 2.554 |
| CDS TE | 0.1564 | 1.681e-05 | 1.714 |
| uORF TE | 0.2402 | 2.667e-11 | 4.379 |

Relationship of CDS initiation context secondary structure EFE (left and right of CDS start) to uORF repressiveness, CDS TE and uORF TE; only the LOWESS trends are shown.

In [34]:

```
x_parameters = ["CDS_sec_struct_EFE_L", "CDS_sec_struct_EFE_R"]
x_labels = [" (-25)", " (+1)", "CDS sec struct EFE (pos w.r.t. start)"]
lowess_versus(data, x_parameters, x_labels, y_parameters, y_labels, colors, limits)
```

Relationship of CDS initiation context secondary structure EFE (left and right of CDS start) to uORF repressiveness, CDS TE and uORF TE; LOWESS trends and underlying scatter plots are shown.

In [35]:

```
x_parameter, x_label = ("CDS_sec_struct_EFE_L", "CDS sec struct EFE (-25 from start)")
lowess_split(one_non_overlapping_uORF, x_parameter, x_label, y_parameters, y_labels, colors, limits)

x_parameter, x_label = ("CDS_sec_struct_EFE_R", "CDS sec struct EFE (+1 from start)")
lowess_split(one_non_overlapping_uORF, x_parameter, x_label, y_parameters, y_labels, colors, limits)
```

CDS sec struct EFE (-25 from start)

|  | Pearson r | p | Fold change |
| --- | --- | --- | --- |
| uORF TE / CDS TE | -0.003853 | 0.9161 | -1.024 |
| CDS TE | 0.1636 | 6.725e-06 | 1.756 |
| uORF TE | 0.08784 | 0.01611 | 1.716 |

CDS sec struct EFE (+1 from start)

|  | Pearson r | p | Fold change |
| --- | --- | --- | --- |
| uORF TE / CDS TE | -0.02608 | 0.4758 | -1.177 |
| CDS TE | 0.1979 | 4.608e-08 | 2.014 |
| uORF TE | 0.08515 | 0.01969 | 1.712 |

#### Comparing secondary structure at CDS and uORF starts¶

There is a small but significant correlation between the amount of secondary structure at uORF and CDS starts. This may explain, in part, why CDS TE increases with reduced secondary structure at uORF starts. However, as to why this correlation even exists between secondary structure at uORF and CDS starts, we do not have an explanation.

In [36]:

```
scatter_lowess(one_non_overlapping_uORF["uORFs_sec_struct_EFE_R"],
               one_non_overlapping_uORF["CDS_sec_struct_EFE_R"],
               "uORF sec struct EFE (+1 from start)",
               "CDS sec struct EFE (+1 from start)",
               corr_table=True)
plt.xlim(-25, 0)
plt.ylim(-25, 0)
```

CDS sec struct EFE (+1 from start) against uORF sec struct EFE (+1 from start)

|  | r/rho | p |
| --- | --- | --- |
| Pearson (linear) | 0.1269 | 0.0004938 |
| Spearman (rank) | 0.1171 | 0.001318 |

Out[36]:

```
(-25, 0)
```

In [37]:

```
scatter_lowess(df_main_filtered["UTR5_mean_ssefe_35"],
               df_main_filtered["CDS_mean_ssefe_35"],
               "5' leader mean sec struct EFE",
               "CDS mean sec struct EFE",
               corr_table=True)
plt.xlim(-22, 0)
plt.ylim(-12.5, -2.5)
```

CDS mean sec struct EFE against 5' leader mean sec struct EFE

|  | r/rho | p |
| --- | --- | --- |
| Pearson (linear) | 0.0701 | 2.54e-12 |
| Spearman (rank) | 0.06649 | 3.13e-11 |

Out[37]:

```
(-12.5, -2.5)
```

**Fig 3c**: Relationship of uORF-CDS distance (shown as the position of the uORF end with respect ot the CDS start) to uORF repressiveness, CDS TE, and uORF TE; only the trends (determined using locally-weighted scatterplot smoothing, or LOWESS) are shown.

In [38]:

```
x_parameter, x_label = ("uORFs_end_pos_wrt_CDS", "uORF end position w.r.t CDS")

limits = ((-300, 0), (-8, 0))
lowess_joint(one_non_overlapping_uORF, x_parameter, x_label, y_parameters, y_labels, colors, limits)
```

**Supp Fig 6c, f, i; Supp Table 1**: Relationship of uORF-CDS distance (shown as the position of the uORF end with respect ot the CDS start) to uORF repressiveness, CDS TE, and uORF TE; LOWESS trends and underlying scatter plots are shown.

In [39]:

```
lowess_split(one_non_overlapping_uORF, x_parameter, x_label, y_parameters, y_labels, colors, limits)
```

uORF end position w.r.t CDS

|  | Pearson r | p | Fold change |
| --- | --- | --- | --- |
| uORF TE / CDS TE | 0.07774 | 0.03328 | 1.53 |
| CDS TE | -0.1394 | 0.0001288 | -1.541 |
| uORF TE | -0.001292 | 0.9718 | -1.007 |

### Integrating various sequence features in linear models for uORF repressiveness¶

In [40]:

```
parameters = {"CDS": (("CDS_wrent_score",
                       "CDS_sec_struct_EFE_L", "CDS_sec_struct_EFE_R", "CDS_mean_ssefe_35"),
                      ("CDS WRENT score",
                       "CDS start sec struct EFE left", "CDS start sec struct EFE right", "CDS mean sec struct EFE"),
                      ((-8, 8), (-20, 0), (-20, 0), (-9, -4)),
                      C_TLOC["CDS start"]),
              "uORF": (("uORFs_wrent_score", "uORFs_sec_struct_EFE_L", "uORFs_sec_struct_EFE_R", "uORF_CDS_log_dist"),
                       ("uORF WRENT score", "uORF start sec struct EFE left", "uORF start sec struct EFE right",
                        "uORF-CDS log distance"),
                       ((-8, 8), (-20, 0), (-20, 0), (0, 3), (1.4, 3), (1, 3)),
                      C_UORF[0]),
              "5' leader": (("log_UTR5_length", "UTR5_mean_ssefe_35"),
                            ("5' leader log10 length", "5' leader mean sec struct EFE"),
                            ((1.4, 3), (-16, -4)),
                            C_TLOC["5' UTR"])}
```

**Fig 3d-e, Table 1, Supp Fig 7a-b**: Linear modelling of uORF repressiveness with uORF, CDS and 5' leader sequence features. Scatter plots show the relationship of uORF repressiveness to a combined score integrating the various sequence features, while the red line indicates the regression (ridge) line; the relative contributions of individual sequence features is shown in the bar graphs below.

The results of using various sequence feature sets to model uORF repressiveness is summarized in a table, comparing the linear correlation and prediction errors (using the predicted residual sum of squares or PRESS statistic).

In [41]:

```
opt = ["CDS", "5' leader"]
permutes = [[i, j] for i in (False, True) for j in (False, True)]
regressor_sets = [["uORF",] + [opt[i] for i, j in enumerate(permute) if j] for permute in permutes]

lin_regress_sets_RidgeCV(regressor_sets, parameters, "Filtered transcripts with one non-overlapping uORF",
                         one_non_overlapping_uORF, "log_ratio", "log2 (uORF TE / CDS TE)",
                         ((-1.5, 1.5), (-7, 3)), (-0.35, 0.35))
```

uORF sequence features

|  | Relative contribution | per unit 2fold change |
| --- | --- | --- |
| Sequence feature |  |  |
| uORF WRENT score | 0.3127 | 0.8654 |
| uORF start sec struct EFE left | 0.05413 | 0.2157 |
| uORF start sec struct EFE right | 0.318 | 1.374 |
| uORF-CDS log distance | -0.2214 | -0.112 |

```

```

uORF + 5' leader sequence features

|  | Relative contribution | per unit 2fold change |
| --- | --- | --- |
| Sequence feature |  |  |
| uORF WRENT score | 0.3216 | 0.8902 |
| uORF start sec struct EFE left | 0.02434 | 0.09699 |
| uORF start sec struct EFE right | 0.2847 | 1.23 |
| uORF-CDS log distance | -0.2853 | -0.1444 |
| 5' leader log10 length | 0.1227 | 0.02587 |
| 5' leader mean sec struct EFE | 0.09132 | 0.2153 |

```

```

uORF + CDS sequence features

|  | Relative contribution | per unit 2fold change |
| --- | --- | --- |
| Sequence feature |  |  |
| uORF WRENT score | 0.3081 | 0.8529 |
| uORF start sec struct EFE left | 0.0908 | 0.3619 |
| uORF start sec struct EFE right | 0.3143 | 1.358 |
| uORF-CDS log distance | -0.2165 | -0.1096 |
| CDS WRENT score | -0.1955 | -0.4752 |
| CDS start sec struct EFE left | -0.01104 | -0.04317 |
| CDS start sec struct EFE right | -0.00765 | -0.03054 |
| CDS mean sec struct EFE | -0.3038 | -0.4023 |

```

```

uORF + CDS + 5' leader sequence features

|  | Relative contribution | per unit 2fold change |
| --- | --- | --- |
| Sequence feature |  |  |
| uORF WRENT score | 0.316 | 0.8746 |
| uORF start sec struct EFE left | 0.06037 | 0.2406 |
| uORF start sec struct EFE right | 0.2807 | 1.213 |
| uORF-CDS log distance | -0.2734 | -0.1383 |
| CDS WRENT score | -0.1929 | -0.4691 |
| CDS start sec struct EFE left | -0.03095 | -0.121 |
| CDS start sec struct EFE right | -0.01784 | -0.07122 |
| CDS mean sec struct EFE | -0.2917 | -0.3864 |
| 5' leader log10 length | 0.1053 | 0.0222 |
| 5' leader mean sec struct EFE | 0.1036 | 0.2441 |

```

```

|  | Pearson r | p | Fold change | PRESS | RESS | n |
| --- | --- | --- | --- | --- | --- | --- |
| Sequence feature set |  |  |  |  |  |  |
| uORF + CDS + 5' leader | 0.2922 | 3.136e-16 | 5.979 | 3,610 | 3,519 | 750 |
| uORF + CDS | 0.288 | 8.714e-16 | 5.609 | 3,602 | 3,529 | 750 |
| uORF + 5' leader | 0.2371 | 4.792e-11 | 4.45 | 3,689 | 3,632 | 750 |
| uORF | 0.2306 | 1.639e-10 | 4.11 | 3,682 | 3,643 | 750 |
