# Supplementary material for: Conservation of uORF repressiveness and sequence features in mouse, human and zebrafish
Source: Nat Commun. 2016 May 24;7:11663. doi: 10.1038/ncomms11663 (PMC4890304; doi:10.1038/ncomms11663)
Supplement: Supplementary Software — Conversions of iPython/Jupyter notebooks documenting all analysis for manuscript. Latest versions of iPython/Jupyer notebooks are available at https://github.com/chewgl/uORF_repressiveness_supplemental [file ncomms11663-s2.zip › Fig 3 (II) - Quantifying and modelling the effects of sequence features on CDS TE - Human.html]

Fig 3 (II) - Quantifying and modelling the effects of sequence features on CDS TE - Human


# Fig 3 (II) - Quantifying and modelling the effects of sequence features on CDS TE¶

## Dataset¶

In [1]:

```
# s = "mm"
# stage = "mES"
# ASSEMBLY = "GRCm38_ens"

# s = "dr"
# stage = "Shield"
# ASSEMBLY = "Zv9_ens"

s = "hs"
stage = "HeLa"
ASSEMBLY = "GRCh37_ens"
```

## Imports, parameters and functions¶

In [2]:

```
# FILTER PARAMETERS
UTR5_LENGTH_MIN = 25
UTR3_LENGTH_MIN = 25

FPKM_MIN = 0.1
CDS_LENGTH_MIN = 100
CDS_READS_MIN = 1

UORF_LENGTH_MIN = 20
UORF_FROM_TRANSCRIPT_START_MIN = UTR5_LENGTH_MIN
UORF_READS_MIN = 1

UTR5_READS_MIN = 1

ORF_END_TRIM = 10
```

In [3]:

```
# IMPORTS
import corebio
import weblogolib

import matplotlib as mpl
import matplotlib.pyplot as plt
import numpy as np
import pandas as pd
import seaborn.apionly as sns

from Bio import SeqIO
from IPython.display import display, HTML, Markdown, Image
from ast import literal_eval
from numpy import argmax, mean, log10, log2, inf, nan, array, float64, subtract, multiply, divide, sign
from pandas import Panel, DataFrame, Series
from pandas.stats.moments import rolling_mean
from scipy.stats import spearmanr, ks_2samp, pearsonr, linregress, zscore, kendalltau, kde
from scipy.stats.mstats import trimboth
from sklearn.linear_model import Ridge, RidgeCV
from statsmodels.distributions import ECDF
from statsmodels.nonparametric.smoothers_lowess import lowess

%matplotlib inline
```

In [4]:

```
pd.options.display.mpl_style = 'default'
pd.options.mode.chained_assignment = None
mpl.rcParams['figure.figsize'] = 8, 6
mpl.rcParams['axes.labelsize'] = 12
mpl.rcParams['axes.titlesize'] = 16
mpl.rcParams['axes.facecolor'] = "#fdfdfd"
mpl.rcParams['grid.alpha'] = 0.5
mpl.rcParams['legend.fontsize'] = 12
mpl.rcParams['font.family'] = 'sans-serif'
mpl.rcParams['figure.autolayout'] = True
mpl.rcParams['savefig.dpi'] = 50
```

In [5]:

```
C_NT = ("#00d700", "#df1f00", "#0226cc", "#ffb700")
C_UORF = ('#b30000', '#e34a33', '#fc8d59', '#fdbb84', '#fdd49e')
C_CDS = ("#105e47", "#157e5f", "#1b9e77", "#48b192", "#76c4ad")
C_RATIO = "#7570b3"
C_BG = ("#000000", "#525252", "#969696", "#d9d9d9")
C_SP = {"hs": "#97543a", "mm": "#546079", "dr": "#3d7463"}
C_SP_BAR = {"hs": "#fc8d62", "mm": "#8da0cb", "dr": "#66c2a5"}
C_NOBIAS = "#ffd92f"
C_SCATTER = "#737373"
C_TREND = "#e41a1c"
C_TLOC = {"5' UTR": "#d95f02", "CDS start": "#1b9e77", "CDS internal": "#e7298a", "3' UTR": "#e6ab02"}
C_STARTS = ("#276419", "#4d9221", "#7fbc41")
C_STOPS = ("#8e0152", "#c51b7d", "#de77ae")
```

In [6]:

```
def scatter_linear_trend(x, y, x_label, y_label, plot_trend=True, corr_table=True):
    data = DataFrame({"x": x, "y": y}).dropna()
    
    plt.figure()
    plt.scatter(data.x, data.y, alpha=0.25, c=C_SCATTER)

    mod = Ridge(normalize=False).fit(zip(*[data.x,]), data.y)
    x1, x2 = (np.percentile(data.x, 2.5), np.percentile(data.x, 97.5))
    y1, y2 = mod.predict([(x1,), (x2,)])
    
    plt.ylim(np.percentile(data.y, 1), np.percentile(data.y, 99))
    plt.xlabel(x_label)
    plt.ylabel(y_label)
    
    if plot_trend:
        plt.plot((x1, x2), (y1, y2), c=C_TREND, lw=2)

    if corr_table:
        to_display = DataFrame(columns=["Correlation\ncoefficient", "p-value"])

        corr, p = pearsonr(data.x, data.y)
        to_display.loc["Pearson linear"] = {"Correlation\ncoefficient": corr,
                                    "p-value": p}
        corr, p = spearmanr(data.x, data.y)
        to_display.loc["Spearman rank"] = {"Correlation\ncoefficient": corr,
                                  "p-value": p}

        display(Markdown(y_label + " against " + x_label))
        display(to_display.applymap('{:,.4g}'.format))
    
    return abs(y2 - y1), sign(y2 - y1)
```

In [7]:

```
def flatten(i):
    return [j for k in i for j in k]

def clean(i):
    return i.replace([-inf, inf], nan).dropna()

def z(i):
    return (i - i.mean()) / i.std(ddof=0)
```

In [8]:

```
def individual_correlations(parameter_set_names_to_correlate, parameter_sets, data_set_name, data_set):
    to_display = DataFrame(columns=["linear correlation", "p", "fold change over 95% of data"])
    
    colors = flatten([[parameters[i][3] for j in range(len(parameters[i][0]))] for i in parameter_set_names_to_correlate])
    
    for set_name in parameter_set_names_to_correlate:
        for parameter, label, limits, _ in zip(*parameters[set_name]):
            if parameter not in data_set.columns:
                continue

            data = clean(data_set[[parameter, "log2_CDS_TE"]])
            
            corr, p = pearsonr(data[parameter], data.log2_CDS_TE)
            
            plt.figure()
            plt.scatter(data[parameter], data.log2_CDS_TE, alpha=0.25, c=C_SCATTER)
            
            plt.xlabel(label)
            plt.ylabel("log2 CDS TE")
            
            plt.xlim(*limits)
            plt.ylim(-8, 0)
            plt.title(data_set_name)
            mod = Ridge(normalize=False).fit(zip(*[data[parameter],]), data.log2_CDS_TE)
            
            x1, x2 = (np.percentile(data[parameter], 2.5), np.percentile(data[parameter], 97.5))
            y1, y2 = mod.predict([(x1,), (x2,)])
            
            plt.plot((x1, x2), (y1, y2), c=C_TREND, lw=2)

            to_display.loc[label] = {"linear correlation": corr, "p": p,
                                     "fold change over 95% of data": 2**(y2 - y1)}

    display(Markdown("### Individual correlations with CDS TE, " + data_set_name[:1].lower() + data_set_name[1:]))
    display(to_display.iloc[::-1].applymap('{:,.4g}'.format))
    
    plt.figure(figsize=(6, 0.75 + float(len(to_display)) * .3))
    ax = to_display["fold change over 95% of data"].apply(log2).plot(kind="barh", width=0.9, color=colors)
    ax.set_xlabel("log2 fold change over 95% of data")
```

In [9]:

```
def lin_regress_sets_RidgeCV(regressor_sets, parameter_sets, data_set_name, data_set,
                             parameter_to_regress, to_regress_label, limits, limits_coeff):
    
    to_display1 = DataFrame(columns=["Pearson r", "p", "Fold change", "PRESS", "RESS", "n"])
    
    sets_to_regress = list(set(flatten(regressor_sets)))
    regressors = [i + "_Z" for i in flatten([parameters[i][0] for i in sets_to_regress])]
    data = data_set[regressors + [parameter_to_regress]].dropna()
    
    for regressor_set in regressor_sets:
        
        regressors = [i + "_Z" for i in flatten([parameter_sets[i][0] for i in regressor_set])]
        regressor_labels = [i for i in flatten([parameter_sets[i][1] for i in regressor_set])]
        colors = flatten([[parameter_sets[i][3] for j in range(len(parameter_sets[i][0]))] for i in regressor_set])

        mod = RidgeCV(normalize=False, store_cv_values=True).fit(data[regressors], data[parameter_to_regress])
        combined_score = np.sum([data[i].multiply(j) for i, j in zip(regressors, mod.coef_)], axis=0)
        
        plt.figure()
        plt.scatter(combined_score, data[parameter_to_regress], alpha=0.25, c=C_SCATTER)
        plt.xlabel("+".join(regressor_set) + " combined score")
        plt.ylabel(to_regress_label)
        plt.xlim(*limits[0])
        plt.ylim(*limits[1])
        plt.title(data_set_name)

        corr, p = pearsonr(combined_score, data[parameter_to_regress])

        x1, x2 = [np.percentile(combined_score, i) for i in [2.5, 97.5]]
        predicted = mod.predict(data[regressors])
        y1, y2 = [np.percentile(predicted, i) for i in [2.5, 97.5]]
        plt.plot((x1, x2), (y1, y2), c=C_TREND, lw=2)
        
        to_display1.loc[" + ".join(regressor_set)] = {"Pearson r": corr, "p": p,
                                                      "Fold change": 2**(y2-y1),
                                                      "PRESS": sum(mod.cv_values_)[0],
                                                      "RESS": sum((predicted - data[parameter_to_regress]) ** 2),
                                                      "n": len(data[parameter_to_regress])}
        
        display(Markdown(" + ".join(regressor_set) + " sequence features"))
        to_display = DataFrame(columns=["Relative contribution", "per unit 2fold change"])
        for i, label, j in zip(regressors, regressor_labels, mod.coef_):
            to_display.loc[label] = {"Relative contribution": j,
                                     "per unit 2fold change": data_set[i[:-2]].std() * j}
        to_display.index.name = "Sequence feature"
        display(to_display.iloc[::-1].applymap('{:,.4g}'.format))
        
        plt.figure(figsize=(6, 0.75 + float(len(to_display)) * .3))
        ax = to_display["Relative contribution"].plot(kind="barh", width=0.9, color=colors)
        ax.set_xlabel("Relative contribution")
        plt.xlim(*limits_coeff)
        plt.title("CDS TE")
        print
    to_display1.index.name = "Sequence feature set"
    display(to_display1.applymap('{:,.4g}'.format))
```

In [10]:

```
def scatter_by_uORF_num(dataset, dataset_label, para_x, para_y,
                        para_x_label, para_y_label, x_limits, y_limits):
    subset_labels = ["1 uORF", "2 uORFs", "3 uORFs", "4+ uORFs"]
    plt.figure()
    data = dataset[[para_x, para_y]]
    data["num_uORF"] = dataset.num_uORFs.apply(lambda x: min(x, 4))
    data = clean(data)

    cmap = mpl.colors.ListedColormap(C_UORF[:-1])

    fig, ax = plt.subplots()
    fig1 = ax.scatter(data[para_x], data[para_y], c=data.num_uORF, s=15,
                      cmap=cmap, vmax=data.num_uORF.max(), alpha=0.3)

    for num_uORF, color in zip([1, 2, 3, 4], C_UORF[:-1]):
        sns.kdeplot(data[para_x][data.num_uORF == num_uORF], data[para_y][data.num_uORF == num_uORF],
                    n_levels=5, cmap=None, colors=color, linewidths=[0,3,0,0,0], alpha=1)
        
    
    sns.kdeplot(data[para_x], data[para_y], n_levels=5, cmap=None,
                colors=C_SCATTER, linewidths=[0,3,0,0,0], alpha=1)

    cbar = fig.colorbar(fig1, ticks=[np.arange(0.4, 5, 0.8)])
    cbar.ax.set_yticklabels(subset_labels)

    plt.xlabel(para_x_label)
    plt.ylabel(para_y_label)
    plt.legend(loc="upper left")
    plt.xlim(*x_limits)
    plt.ylim(*y_limits)
    plt.title(dataset_label)
    
    to_display = DataFrame(columns=["Correlation\ncoefficient", "p-value"])

    for num_uORF, label in zip((1, 2, 3, 4), subset_labels):
        corr, p = pearsonr(data[para_x][data.num_uORF == num_uORF],
                           data[para_y][data.num_uORF == num_uORF])
        to_display.loc[label] = {"Correlation\ncoefficient": corr,
                                                   "p-value": p}
    corr, p = pearsonr(data[para_x], data[para_y])
    to_display.loc["All"] = {"Correlation\ncoefficient": corr,
                                "p-value": p}
    display(Markdown(para_y_label + " against " + para_x_label))
    display(to_display.applymap('{:,.4g}'.format))
```

## Reading in ORF characteristics data¶

In [11]:

```
DATA_DIR = "./data/" + s + "/"
ANNOTATIONS_DIR = "./annotations/"
```

In [12]:

```
CONVERTERS = {i:literal_eval for i in ("uORFs_reads", "uORFs_length", "uORFs_wrent_score", "uORFs_urent_score",
                                       "uORFs_wrent_seq", "uORFs_sec_struct_EFE_L", "uORFs_sec_struct_EFE_R",
                                       "uORFs_start_pos_wrt_tss", "uORFs_end_pos_wrt_CDS",
                                       "ORFs_wrent_score", "ORFs_urent_score", "ORFs_wrent_seq",
                                       "ORFs_sec_struct_EFE_L", "ORFs_sec_struct_EFE_R")}
```

In [13]:

```
stage_species = {"mES": "mm", "HeLa": "hs", "Shield": "dr"}
mean_ssefes = pd.read_table(DATA_DIR + stage_species[stage] + "_mean_ssefes.df", sep="\t", index_col=[0])
```

In [14]:

```
windows = [25, 30, 35, 40]
df_main = pd.read_csv(DATA_DIR + stage + "_main.df", index_col="Transcript", sep="\t", converters=CONVERTERS)
for window in windows:
    for pos in ("UTR5", "CDS"):
        field = pos + "_mean_ssefe_" + str(window)
        df_main[field] = mean_ssefes[field][mean_ssefes.index.isin(df_main.index)]
        df_main[field + "_Z"] = z(df_main[field])
```

In [15]:

```
df_main_filtered = df_main[(df_main.UTR5_length >= UTR5_LENGTH_MIN) &
                           (df_main.UTR3_length >= UTR3_LENGTH_MIN) &
                           (df_main.Gene_Expression_FPKM >= FPKM_MIN) &
                           (df_main.CDS_length >= CDS_LENGTH_MIN) &
                           (df_main.CDS_reads >= CDS_READS_MIN) &
                           (df_main.UTR5_reads_trunc >= UTR5_READS_MIN)]
```

In [16]:

```
df_main_filtered["CDS_density"] = df_main_filtered.CDS_reads / \
                                  df_main_filtered.CDS_length.subtract(ORF_END_TRIM)

df_main_filtered["CDS_TE"] = df_main_filtered.CDS_density / \
                             df_main_filtered.Gene_Expression_FPKM

df_main_filtered["log2_CDS_density"] = log2(df_main_filtered.CDS_density)

df_main_filtered["log2_CDS_TE"] = log2(df_main_filtered.CDS_TE)

df_main_filtered["num_uORFs"] = df_main_filtered.num_uORFs.replace([0], 0.1)

df_main_filtered["num_uORFs_density"] = df_main_filtered.num_uORFs.apply(float) / \
                                        df_main_filtered.UTR5_length

for parameter in ("CDS_TE", "CDS_length", "UTR5_length", "Gene_Expression_FPKM", "num_uORFs_density"):
    df_main_filtered["log_" + parameter] = clean(log10(df_main_filtered[parameter].apply(float)))
    df_main_filtered["log_" + parameter + "_Z"] = z(df_main_filtered["log_" + parameter])

for parameter in ("CDS_wrent_score", "CDS_sec_struct_EFE_L", "CDS_sec_struct_EFE_R",
                  "UTR5_GC", "CDS_GC", "num_uORFs"):
    df_main_filtered[parameter + "_Z"] = z(df_main_filtered[parameter])
```

## Restricting uORF sequence feature effect analyses to transcripts with only one non-overlapping uORF¶

In [17]:

```
no_uORFs = df_main_filtered[df_main_filtered.num_uORFs < 1]
with_uORFs = df_main_filtered[df_main_filtered.num_uORFs >= 1]
one_uORF = df_main_filtered[df_main_filtered.num_uORFs == 1]
two_uORF = df_main_filtered[df_main_filtered.num_uORFs == 2]
three_uORF = df_main_filtered[df_main_filtered.num_uORFs == 3]

for parameter in ("CDS_TE", "CDS_length", "UTR5_length", "Gene_Expression_FPKM", "num_uORFs_density"):
    no_uORFs["log_" + parameter + "_Z"] = z(no_uORFs["log_" + parameter])

for parameter in ("CDS_wrent_score", "CDS_sec_struct_EFE_L", "CDS_sec_struct_EFE_R", "UTR5_GC", "CDS_GC", "num_uORFs"):
    no_uORFs[parameter + "_Z"] = z(no_uORFs[parameter])

for parameter in ("CDS_TE", "CDS_length", "UTR5_length", "Gene_Expression_FPKM", "num_uORFs_density"):
    with_uORFs["log_" + parameter + "_Z"] = z(with_uORFs["log_" + parameter])

for parameter in ("CDS_wrent_score", "CDS_sec_struct_EFE_L", "CDS_sec_struct_EFE_R", "UTR5_GC", "CDS_GC", "num_uORFs"):
    with_uORFs[parameter + "_Z"] = z(with_uORFs[parameter])
```

In [18]:

```
# Creates set of 1 non-overlapping uORF
one_non_overlapping_uORF = df_main_filtered[(df_main_filtered.num_uORFs == 1)]   # 1 uORF

# Since set has only transcripts with 1 uORF, "singularize" some columns of data
for variable in ("uORFs_reads", "uORFs_length", "uORFs_wrent_score", "uORFs_urent_score",
                 "uORFs_sec_struct_EFE_L", "uORFs_sec_struct_EFE_R", "uORFs_start_pos_wrt_tss",
                 "uORFs_end_pos_wrt_CDS"):
    one_non_overlapping_uORF[variable] = one_non_overlapping_uORF[variable].apply(lambda x:x[0])

# Filter set for non-overlapping uORF, with uORFs of minimum length, reads and distance from transcript start
one_non_overlapping_uORF = one_non_overlapping_uORF[\
    (one_non_overlapping_uORF.uORFs_end_pos_wrt_CDS < 0) &
    (one_non_overlapping_uORF.uORFs_reads >= UORF_READS_MIN) &
    (one_non_overlapping_uORF.uORFs_length >= UORF_LENGTH_MIN) &
    (one_non_overlapping_uORF.uORFs_start_pos_wrt_tss >= UORF_FROM_TRANSCRIPT_START_MIN)]
print "Number of one non-overlapping uORF Transcripts = %d" % len(one_non_overlapping_uORF)

# Calculate uORF TEs for set
one_non_overlapping_uORF["uORF_TE"] = one_non_overlapping_uORF.apply(lambda x: float64(x.uORFs_reads) \
                                                                               / (x.uORFs_length - ORF_END_TRIM) \
                                                                               / x.Gene_Expression_FPKM, axis=1)
```

```
Number of one non-overlapping uORF Transcripts = 677
```

In [19]:

```
one_non_overlapping_uORF["uORF_CDS_log_dist"] = one_non_overlapping_uORF.uORFs_end_pos_wrt_CDS.apply(lambda x:log10(-x))

for parameter in ("uORFs_length", "uORFs_start_pos_wrt_tss"):
    one_non_overlapping_uORF["log_" + parameter] = log10(one_non_overlapping_uORF[parameter].apply(float))
    one_non_overlapping_uORF["log_" + parameter + "_Z"] = z(one_non_overlapping_uORF["log_" + parameter])

for parameter in ("uORFs_wrent_score", "uORFs_sec_struct_EFE_L", "uORFs_sec_struct_EFE_R", "uORF_CDS_log_dist"):
    one_non_overlapping_uORF[parameter + "_Z"] = z(one_non_overlapping_uORF[parameter])

for parameter in ("CDS_TE", "CDS_length", "UTR5_length", "Gene_Expression_FPKM", "num_uORFs_density"):
    one_non_overlapping_uORF["log_" + parameter + "_Z"] = z(one_non_overlapping_uORF["log_" + parameter])

for parameter in ("CDS_wrent_score", "CDS_sec_struct_EFE_L", "CDS_sec_struct_EFE_R", "UTR5_GC", "CDS_GC", "num_uORFs"):
    one_non_overlapping_uORF[parameter + "_Z"] = z(one_non_overlapping_uORF[parameter])
```

## Figures¶

### Integrating various sequence features in linear models¶

In [20]:

```
parameters = {"CDS": (("CDS_wrent_score", 
                       "CDS_sec_struct_EFE_L", "CDS_sec_struct_EFE_R", "CDS_mean_ssefe_35"),
                      ("CDS WRENT score", 
                       "CDS start sec struct EFE left", "CDS start sec struct EFE right", "CDS mean sec struct EFE"),
                      ((-6, 6), (-18, 0), (-18, 0), (-11, -3)),
                      C_TLOC["CDS start"]),
              "uORF": (("uORFs_wrent_score", "uORFs_sec_struct_EFE_L", "uORFs_sec_struct_EFE_R",
                        "uORF_CDS_log_dist", "log_uORFs_length", "log_uORFs_start_pos_wrt_tss"),
                       ("uORF WRENT score", "uORF start sec struct EFE left", "uORF start sec struct EFE right",
                        "uORF-CDS log distance", "log uORF length"),#, "log uORF start pos wrt tss"),
                       ((-8, 8), (-20, 0), (-20, 0), (0, 3), (1.4, 3)),#, (1, 3)),
                       C_UORF[0]),
              "5' leader": (("log_UTR5_length", "UTR5_mean_ssefe_35", "log_num_uORFs_density"),
                            ("5' leader log10 length", "5' leader mean sec struct EFE", "log10 uORF density"),
                            ((1.4, 3.2), (-16, -3), (-3.75, -1.5)),
                            C_TLOC["5' UTR"]),
              "5' leader (fixed uORFs)": (("log_UTR5_length", "UTR5_mean_ssefe_35"),
                            ("5' leader log10 length", "5' leader mean sec struct EFE"),
                            ((1.4, 2.8), (-17, -4)),
                            C_TLOC["5' UTR"]),
#               "5' leader": (("log_UTR5_length", "UTR5_mean_ssefe_35"),
#                             ("5' leader log10 length", "5' leader mean sec struct EFE"),
#                             ((1.4, 3), (-16, -4)),
#                             C_TLOC["5' UTR"]),
              "others": (("log_Gene_Expression_FPKM",),
                         ("Gene Expression (log10 FPKM)",),
                         ((-1, 3.5),),
                         C_BG[1])}
```

**Supp Fig 8a, Supp Table 2**: Verifying correlation of individual CDS sequence features to CDS TE for all filtered transcripts

In [21]:

```
individual_correlations(("CDS", "5' leader", "others"), parameters, "All filtered transcripts", df_main_filtered)
```

### Individual correlations with CDS TE, all filtered transcripts¶

|  | linear correlation | p | fold change over 95% of data |
| --- | --- | --- | --- |
| Gene Expression (log10 FPKM) | -0.1095 | 4.139e-26 | 0.5401 |
| log10 uORF density | -0.2155 | 7.646e-98 | 0.3638 |
| 5' leader mean sec struct EFE | 0.1139 | 8.962e-28 | 1.854 |
| 5' leader log10 length | -0.1828 | 1.899e-70 | 0.3624 |
| CDS mean sec struct EFE | 0.3574 | 2.056e-273 | 6.059 |
| CDS start sec struct EFE right | 0.09451 | 7.561e-20 | 1.659 |
| CDS start sec struct EFE left | 0.08652 | 7.124e-17 | 1.593 |
| CDS WRENT score | 0.1145 | 1.899e-28 | 1.875 |

```
c:\Anaconda2\lib\site-packages\matplotlib\collections.py:590: FutureWarning: elementwise comparison failed; returning scalar instead, but in the future will perform elementwise comparison
  if self._edgecolors == str('face'):
c:\Anaconda2\lib\site-packages\matplotlib\figure.py:1653: UserWarning: This figure includes Axes that are not compatible with tight_layout, so its results might be incorrect.
  warnings.warn("This figure includes Axes that are not "
```

**Supp Fig 8a, Supp Table 2**: Verifying correlation of individual CDS sequence features to CDS TE for transcripts with uORFs

In [22]:

```
individual_correlations(("CDS", "5' leader", "others"), parameters, "Filtered transcripts with uORFs", with_uORFs)
```

### Individual correlations with CDS TE, filtered transcripts with uORFs¶

|  | linear correlation | p | fold change over 95% of data |
| --- | --- | --- | --- |
| Gene Expression (log10 FPKM) | -0.07531 | 2.638e-08 | 0.6528 |
| log10 uORF density | -0.06357 | 2.671e-06 | 0.7127 |
| 5' leader mean sec struct EFE | 0.1427 | 4.344e-26 | 2.171 |
| 5' leader log10 length | -0.1129 | 6.469e-17 | 0.5233 |
| CDS mean sec struct EFE | 0.3607 | 1.917e-166 | 6.038 |
| CDS start sec struct EFE right | 0.1032 | 2.238e-14 | 1.741 |
| CDS start sec struct EFE left | 0.1058 | 4.924e-15 | 1.772 |
| CDS WRENT score | 0.08637 | 1.727e-10 | 1.609 |

**Supp Fig 8g, Supp Table 2**: Verifying correlation of individual CDS sequence features to CDS TE for transcripts with no uORFs

In [23]:

```
individual_correlations(("CDS", "5' leader (fixed uORFs)", "others"), parameters, "Filtered transcripts without uORFs", no_uORFs)
```

### Individual correlations with CDS TE, filtered transcripts without uORFs¶

|  | linear correlation | p | fold change over 95% of data |
| --- | --- | --- | --- |
| Gene Expression (log10 FPKM) | -0.244 | 5.839e-53 | 0.2772 |
| 5' leader mean sec struct EFE | 0.2312 | 3.378e-46 | 3.29 |
| 5' leader log10 length | -0.02274 | 0.1597 | 0.8924 |
| CDS mean sec struct EFE | 0.4038 | 1.556e-145 | 6.806 |
| CDS start sec struct EFE right | 0.1696 | 4.383e-26 | 2.327 |
| CDS start sec struct EFE left | 0.1677 | 1.539e-25 | 2.319 |
| CDS WRENT score | 0.1228 | 2.526e-14 | 1.85 |

#### Integrating various sequence features in linear model for CDS TE¶

Linear modelling of uORF repressiveness with uORF, CDS and 5' leader sequence features. Scatter plots show the relationship of uORF repressiveness to a combined score integrating the various sequence features, while the red line indicates the regression (ridge) line; the relative contributions of individual sequence features is shown in the bar graphs below.

The results of using various sequence feature sets to model uORF repressiveness is summarized in a table, comparing the linear correlation and prediction errors (using the predicted residual sum of squares or PRESS statistic).

**Fig 3f-g, Table 1, Supp Fig 7c-h**: Transcripts with one non-overlapping uORF

In [24]:

```
opt = ["5' leader (fixed uORFs)", "uORF"]
# opt = ["5' leader", "uORF"]
permutes = [[i, j] for i in (False, True) for j in (False, True)]
regressor_sets = [["CDS",] + [opt[i] for i, j in enumerate(permute) if j] for permute in permutes]

lin_regress_sets_RidgeCV(regressor_sets, parameters, "Filtered transcripts with one non-overlapping uORFs",
                         one_non_overlapping_uORF, "log2_CDS_TE", "log2 CDS TE", ((-2.5, 2), (-10, 0)), (-0.25, 0.8))
```

CDS sequence features

|  | Relative contribution | per unit 2fold change |
| --- | --- | --- |
| Sequence feature |  |  |
| CDS mean sec struct EFE | 0.7598 | 1.389 |
| CDS start sec struct EFE right | 0.07626 | 0.3214 |
| CDS start sec struct EFE left | 0.2927 | 1.174 |
| CDS WRENT score | -0.0078 | -0.01727 |

```

```

CDS + uORF sequence features

|  | Relative contribution | per unit 2fold change |
| --- | --- | --- |
| Sequence feature |  |  |
| log uORF length | 0.0264 | 0.007245 |
| uORF-CDS log distance | 0.2055 | 0.1019 |
| uORF start sec struct EFE right | 0.1812 | 0.7899 |
| uORF start sec struct EFE left | 0.2029 | 0.8306 |
| uORF WRENT score | -0.2145 | -0.5418 |
| CDS mean sec struct EFE | 0.7453 | 1.363 |
| CDS start sec struct EFE right | 0.05285 | 0.2227 |
| CDS start sec struct EFE left | 0.2365 | 0.9489 |
| CDS WRENT score | -0.01076 | -0.02382 |

```

```

CDS + 5' leader (fixed uORFs) sequence features

|  | Relative contribution | per unit 2fold change |
| --- | --- | --- |
| Sequence feature |  |  |
| 5' leader mean sec struct EFE | 0.5621 | 1.35 |
| 5' leader log10 length | 0.1305 | 0.02716 |
| CDS mean sec struct EFE | 0.6938 | 1.269 |
| CDS start sec struct EFE right | 0.04625 | 0.1949 |
| CDS start sec struct EFE left | 0.1234 | 0.495 |
| CDS WRENT score | 0.04074 | 0.09018 |

```

```

CDS + 5' leader (fixed uORFs) + uORF sequence features

|  | Relative contribution | per unit 2fold change |
| --- | --- | --- |
| Sequence feature |  |  |
| log uORF length | -0.1074 | -0.02948 |
| uORF-CDS log distance | 0.009889 | 0.004904 |
| uORF start sec struct EFE right | 0.05324 | 0.232 |
| uORF start sec struct EFE left | 0.06586 | 0.2697 |
| uORF WRENT score | -0.2085 | -0.5265 |
| 5' leader mean sec struct EFE | 0.476 | 1.143 |
| 5' leader log10 length | 0.2954 | 0.06149 |
| CDS mean sec struct EFE | 0.7068 | 1.292 |
| CDS start sec struct EFE right | 0.03851 | 0.1623 |
| CDS start sec struct EFE left | 0.1383 | 0.555 |
| CDS WRENT score | 0.01504 | 0.03329 |

```

```

|  | Pearson r | p | Fold change | PRESS | RESS | n |
| --- | --- | --- | --- | --- | --- | --- |
| Sequence feature set |  |  |  |  |  |  |
| CDS | 0.4901 | 3.442e-42 | 9.979 | 1,908 | 1,884 | 677 |
| CDS + uORF | 0.5362 | 1.2e-51 | 13.8 | 1,820 | 1,766 | 677 |
| CDS + 5' leader (fixed uORFs) | 0.5417 | 6.81e-53 | 13.68 | 1,783 | 1,751 | 677 |
| CDS + 5' leader (fixed uORFs) + uORF | 0.5612 | 1.93e-57 | 14.79 | 1,758 | 1,698 | 677 |

**Supp Fig S8b-c, e-f, h-i, Supp Table 3**: All filtered transcripts, transcripts with uORFs, transcripts without uORFs

In [25]:

```
for t_set, t_set_label, UTR5_type in zip((df_main_filtered, with_uORFs, no_uORFs),
                                         ("All filtered transcripts",
                                          "Filtered transcripts with uORFs",
                                          "Filtered transcripts without uORFs"),
                                         ("5' leader", "5' leader", "5' leader (fixed uORFs)")):
    display(Markdown("**" + t_set_label + "**"))
    lin_regress_sets_RidgeCV([["CDS",], ["CDS", UTR5_type]], parameters, t_set_label,
                             t_set, "log2_CDS_TE", "log2 CDS TE", ((-2.5, 2), (-10, 0)), (-0.25, 0.8))
```

**All filtered transcripts**

CDS sequence features

|  | Relative contribution | per unit 2fold change |
| --- | --- | --- |
| Sequence feature |  |  |
| CDS mean sec struct EFE | 0.7359 | 1.276 |
| CDS start sec struct EFE right | -0.05224 | -0.2158 |
| CDS start sec struct EFE left | -0.00926 | -0.03596 |
| CDS WRENT score | 0.1394 | 0.3103 |

```

```

CDS + 5' leader sequence features

|  | Relative contribution | per unit 2fold change |
| --- | --- | --- |
| Sequence feature |  |  |
| log10 uORF density | -0.4871 | -0.2777 |
| 5' leader mean sec struct EFE | 0.3295 | 0.8955 |
| 5' leader log10 length | -0.3414 | -0.1207 |
| CDS mean sec struct EFE | 0.6923 | 1.2 |
| CDS start sec struct EFE right | -0.01086 | -0.04485 |
| CDS start sec struct EFE left | -0.00191 | -0.007415 |
| CDS WRENT score | 0.08902 | 0.1982 |

```

```

|  | Pearson r | p | Fold change | PRESS | RESS | n |
| --- | --- | --- | --- | --- | --- | --- |
| Sequence feature set |  |  |  |  |  |  |
| CDS | 0.3643 | 9.012e-285 | 6.456 | 3.23e+04 | 3.227e+04 | 9,136 |
| CDS + 5' leader | 0.4737 | 0 | 12.71 | 2.891e+04 | 2.886e+04 | 9,136 |

**Filtered transcripts with uORFs**

CDS sequence features

|  | Relative contribution | per unit 2fold change |
| --- | --- | --- |
| Sequence feature |  |  |
| CDS mean sec struct EFE | 0.7444 | 1.297 |
| CDS start sec struct EFE right | -0.05245 | -0.21 |
| CDS start sec struct EFE left | 0.0102 | 0.0389 |
| CDS WRENT score | 0.08611 | 0.1947 |

```

```

CDS + 5' leader sequence features

|  | Relative contribution | per unit 2fold change |
| --- | --- | --- |
| Sequence feature |  |  |
| log10 uORF density | -0.363 | -0.1024 |
| 5' leader mean sec struct EFE | 0.3716 | 0.9312 |
| 5' leader log10 length | -0.3517 | -0.1105 |
| CDS mean sec struct EFE | 0.6879 | 1.199 |
| CDS start sec struct EFE right | -0.044 | -0.1762 |
| CDS start sec struct EFE left | -0.01593 | -0.06079 |
| CDS WRENT score | 0.08012 | 0.1811 |

```

```

|  | Pearson r | p | Fold change | PRESS | RESS | n |
| --- | --- | --- | --- | --- | --- | --- |
| Sequence feature set |  |  |  |  |  |  |
| CDS | 0.3637 | 2.419e-169 | 6.141 | 1.936e+04 | 1.933e+04 | 5,426 |
| CDS + 5' leader | 0.4179 | 2.502e-228 | 9.146 | 1.843e+04 | 1.838e+04 | 5,426 |

**Filtered transcripts without uORFs**

CDS sequence features

|  | Relative contribution | per unit 2fold change |
| --- | --- | --- |
| Sequence feature |  |  |
| CDS mean sec struct EFE | 0.7153 | 1.228 |
| CDS start sec struct EFE right | 0.05585 | 0.2357 |
| CDS start sec struct EFE left | 0.1315 | 0.5065 |
| CDS WRENT score | 0.08825 | 0.1908 |

```

```

CDS + 5' leader (fixed uORFs) sequence features

|  | Relative contribution | per unit 2fold change |
| --- | --- | --- |
| Sequence feature |  |  |
| 5' leader mean sec struct EFE | 0.3226 | 0.9116 |
| 5' leader log10 length | -0.06079 | -0.01794 |
| CDS mean sec struct EFE | 0.6983 | 1.199 |
| CDS start sec struct EFE right | 0.03823 | 0.1614 |
| CDS start sec struct EFE left | 0.02346 | 0.09039 |
| CDS WRENT score | 0.0966 | 0.2089 |

```

```

|  | Pearson r | p | Fold change | PRESS | RESS | n |
| --- | --- | --- | --- | --- | --- | --- |
| Sequence feature set |  |  |  |  |  |  |
| CDS | 0.4163 | 1.912e-155 | 7.442 | 1.071e+04 | 1.069e+04 | 3,710 |
| CDS + 5' leader (fixed uORFs) | 0.445 | 5.847e-180 | 8.512 | 1.04e+04 | 1.037e+04 | 3,710 |

#### Tracking correlations amongst 5' leader sequence features¶

**Supp Fig S14a:** Correlations between 5' leader GC content, density of uORFs and mean secondary structure EFE

In [26]:

```
parameters = ["UTR5_GC", "UTR5_mean_ssefe_35"]
data = clean(with_uORFs[["log_num_uORFs_density",] + parameters])

ax = plt.scatter(data.UTR5_mean_ssefe_35, data.log_num_uORFs_density, c=data.UTR5_GC,
                 vmin=0.35, vmax=0.85, cmap="Greys", s=30, alpha=0.5)

plt.xlim(-15, -3)
plt.xlabel("5' leader mean sec struct EFE")
plt.ylim(-3, -1.5)
plt.ylabel("5' leader log10 density of uORFs")
plt.title("Filtered transcripts with uORFs")
plt.colorbar(ax, label="5' leader GC content")

data.columns = ["log10 density of uORFs", "GC content", "Mean sec struct EFE"]
data.corr()
```

Out[26]:

|  | log10 density of uORFs | GC content | Mean sec struct EFE |
| --- | --- | --- | --- |
| log10 density of uORFs | 1.000000 | -0.392773 | 0.288289 |
| GC content | -0.392773 | 1.000000 | -0.886569 |
| Mean sec struct EFE | 0.288289 | -0.886569 | 1.000000 |

Individual correlations between 5' leader GC content, density of uORFs and mean secondary structure EFE

In [27]:

```
parameters = ["UTR5_GC", "UTR5_mean_ssefe_35"]
data = clean(with_uORFs[["log_num_uORFs_density",] + parameters])

for parameter, parameter_label, limits in zip(parameters,
                                              ("5' leader GC content", "5' leader mean sec struct EFE"),
                                              ((0.35, 0.85), (-15, -3))):
    
    scatter_linear_trend(data[parameter], data.log_num_uORFs_density,
                         parameter_label, "5' leader log10 uORF density")
    plt.xlim(*limits)
    plt.ylim(-2.6, -1.6)
    plt.title("Filtered transcripts with uORFs")

scatter_linear_trend(data.UTR5_GC, data.UTR5_mean_ssefe_35,
                     "5' leader GC content", "5' leader mean sec struct EFE")
plt.xlim(0.35, 0.85)
plt.ylim(-15, -3)
plt.title("Filtered transcripts with uORFs")
```

5' leader log10 uORF density against 5' leader GC content

|  | Correlation coefficient | p-value |
| --- | --- | --- |
| Pearson linear | -0.3928 | 1.236e-199 |
| Spearman rank | -0.3949 | 5.146e-202 |

5' leader log10 uORF density against 5' leader mean sec struct EFE

|  | Correlation coefficient | p-value |
| --- | --- | --- |
| Pearson linear | 0.2883 | 2.384e-104 |
| Spearman rank | 0.318 | 8.778e-128 |

5' leader mean sec struct EFE against 5' leader GC content

|  | Correlation coefficient | p-value |
| --- | --- | --- |
| Pearson linear | -0.8866 | 0 |
| Spearman rank | -0.8923 | 0 |

Out[27]:

```
<matplotlib.text.Text at 0x20608c18>
```

**Supp Fig S14b:** Correlations between select sequence parameters, subsetted by number of uORFs per transcripts.

In [28]:

```
parameters = DataFrame({"log_CDS_TE": {"label": "log10 CDS TE",
                                       "limits": (-2.5, 0)},
                        "log_UTR5_TE": {"label": "log10 5' leader TE",
                                        "limits": (-3.5, 0)},
                        "log_CDS_density": {"label": "log10 CDS RP read density",
                                            "limits": (-2.5, 1.5)},
                        "log_UTR5_density": {"label": "log10 5' leader RP read density",
                                             "limits": (-3, 1)},
                        "log_Gene_Expression_FPKM": {"label": "log10 gene expression (FPKM)",
                                                     "limits": (-1, 2.5)},
                        "log_UTR5_length": {"label": "log10 5' leader length",
                                            "limits": (1.8, 3)},
                        "log_num_uORFs_density": {"label": "log10 5' leader density of uORFs",
                                                  "limits": (-3, -1.5)},
                        "UTR5_GC": {"label": "5' leader GC content",
                                    "limits": (0.35, 0.85)},
                        "UTR5_mean_ssefe_35": {"label": "5' leader mean sec struct EFE",
                                               "limits": (-16, -4)}})
```

In [29]:

```
dataset, dataset_label = with_uORFs, "Filtered transcripts with uORFs"
comparisons = [("UTR5_GC", "log_UTR5_length"),
               ("UTR5_GC", "UTR5_mean_ssefe_35"),
               ("UTR5_GC", "log_num_uORFs_density"),
               ("log_UTR5_length", "UTR5_GC"),
               ("log_UTR5_length", "UTR5_mean_ssefe_35"),
               ("log_UTR5_length", "log_num_uORFs_density"),
               ("UTR5_mean_ssefe_35", "UTR5_GC"),
               ("UTR5_mean_ssefe_35", "log_UTR5_length"),
               ("UTR5_mean_ssefe_35", "log_num_uORFs_density"),
               ("log_num_uORFs_density", "UTR5_GC"),
               ("log_num_uORFs_density", "log_UTR5_length"),
               ("log_num_uORFs_density", "UTR5_mean_ssefe_35"),
               ("UTR5_GC", "log_CDS_TE"),
               ("log_UTR5_length", "log_CDS_TE"),
               ("UTR5_mean_ssefe_35", "log_CDS_TE"),
               ("log_num_uORFs_density", "log_CDS_TE")]

for x, y in comparisons:
    scatter_by_uORF_num(dataset, dataset_label, x, y,
                        parameters[x].label, parameters[y].label,
                        parameters[x].limits, parameters[y].limits)
```

```
c:\Anaconda2\lib\site-packages\matplotlib\collections.py:650: FutureWarning: elementwise comparison failed; returning scalar instead, but in the future will perform elementwise comparison
  if self._edgecolors_original != str('face'):
c:\Anaconda2\lib\site-packages\matplotlib\axes\_axes.py:475: UserWarning: No labelled objects found. Use label='...' kwarg on individual plots.
  warnings.warn("No labelled objects found. "
```

log10 5' leader length against 5' leader GC content

|  | Correlation coefficient | p-value |
| --- | --- | --- |
| 1 uORF | 0.1133 | 2.026e-07 |
| 2 uORFs | 0.1425 | 1.507e-06 |
| 3 uORFs | 0.2524 | 4.301e-11 |
| 4+ uORFs | 0.01049 | 0.679 |
| All | -0.181 | 2.524e-41 |

5' leader mean sec struct EFE against 5' leader GC content

|  | Correlation coefficient | p-value |
| --- | --- | --- |
| 1 uORF | -0.8344 | 0 |
| 2 uORFs | -0.8714 | 0 |
| 3 uORFs | -0.8934 | 6.855e-232 |
| 4+ uORFs | -0.923 | 0 |
| All | -0.8866 | 0 |

log10 5' leader density of uORFs against 5' leader GC content

|  | Correlation coefficient | p-value |
| --- | --- | --- |
| 1 uORF | -0.1133 | 2.026e-07 |
| 2 uORFs | -0.1425 | 1.507e-06 |
| 3 uORFs | -0.2524 | 4.301e-11 |
| 4+ uORFs | -0.4887 | 2.458e-94 |
| All | -0.3908 | 3.262e-198 |

5' leader GC content against log10 5' leader length

|  | Correlation coefficient | p-value |
| --- | --- | --- |
| 1 uORF | 0.1133 | 2.026e-07 |
| 2 uORFs | 0.1425 | 1.507e-06 |
| 3 uORFs | 0.2524 | 4.301e-11 |
| 4+ uORFs | 0.01049 | 0.679 |
| All | -0.181 | 2.524e-41 |

5' leader mean sec struct EFE against log10 5' leader length

|  | Correlation coefficient | p-value |
| --- | --- | --- |
| 1 uORF | 0.01452 | 0.5085 |
| 2 uORFs | -0.05899 | 0.04743 |
| 3 uORFs | -0.1646 | 2.041e-05 |
| 4+ uORFs | 0.003726 | 0.8832 |
| All | 0.2167 | 1.187e-58 |

log10 5' leader density of uORFs against log10 5' leader length

|  | Correlation coefficient | p-value |
| --- | --- | --- |
| 1 uORF | -1 | 0 |
| 2 uORFs | -1 | 0 |
| 3 uORFs | -1 | 0 |
| 4+ uORFs | -0.4378 | 5.712e-74 |
| All | -0.2609 | 2.008e-85 |

5' leader GC content against 5' leader mean sec struct EFE

|  | Correlation coefficient | p-value |
| --- | --- | --- |
| 1 uORF | -0.8344 | 0 |
| 2 uORFs | -0.8714 | 0 |
| 3 uORFs | -0.8934 | 6.855e-232 |
| 4+ uORFs | -0.923 | 0 |
| All | -0.8866 | 0 |

log10 5' leader length against 5' leader mean sec struct EFE

|  | Correlation coefficient | p-value |
| --- | --- | --- |
| 1 uORF | 0.01452 | 0.5085 |
| 2 uORFs | -0.05899 | 0.04743 |
| 3 uORFs | -0.1646 | 2.041e-05 |
| 4+ uORFs | 0.003726 | 0.8832 |
| All | 0.2167 | 1.187e-58 |

log10 5' leader density of uORFs against 5' leader mean sec struct EFE

|  | Correlation coefficient | p-value |
| --- | --- | --- |
| 1 uORF | -0.01452 | 0.5085 |
| 2 uORFs | 0.05899 | 0.04743 |
| 3 uORFs | 0.1646 | 2.041e-05 |
| 4+ uORFs | 0.4032 | 5.514e-62 |
| All | 0.2883 | 2.384e-104 |

5' leader GC content against log10 5' leader density of uORFs

|  | Correlation coefficient | p-value |
| --- | --- | --- |
| 1 uORF | -0.1133 | 2.026e-07 |
| 2 uORFs | -0.1425 | 1.507e-06 |
| 3 uORFs | -0.2524 | 4.301e-11 |
| 4+ uORFs | -0.4887 | 2.458e-94 |
| All | -0.3908 | 3.262e-198 |

```
c:\Anaconda2\lib\site-packages\matplotlib\pyplot.py:424: RuntimeWarning: More than 20 figures have been opened. Figures created through the pyplot interface (`matplotlib.pyplot.figure`) are retained until explicitly closed and may consume too much memory. (To control this warning, see the rcParam `figure.max_open_warning`).
  max_open_warning, RuntimeWarning)
```

log10 5' leader length against log10 5' leader density of uORFs

|  | Correlation coefficient | p-value |
| --- | --- | --- |
| 1 uORF | -1 | 0 |
| 2 uORFs | -1 | 0 |
| 3 uORFs | -1 | 0 |
| 4+ uORFs | -0.4378 | 5.712e-74 |
| All | -0.2609 | 2.008e-85 |

5' leader mean sec struct EFE against log10 5' leader density of uORFs

|  | Correlation coefficient | p-value |
| --- | --- | --- |
| 1 uORF | -0.01452 | 0.5085 |
| 2 uORFs | 0.05899 | 0.04743 |
| 3 uORFs | 0.1646 | 2.041e-05 |
| 4+ uORFs | 0.4032 | 5.514e-62 |
| All | 0.2883 | 2.384e-104 |

log10 CDS TE against 5' leader GC content

|  | Correlation coefficient | p-value |
| --- | --- | --- |
| 1 uORF | -0.2896 | 1.013e-41 |
| 2 uORFs | -0.2613 | 4.192e-19 |
| 3 uORFs | -0.2827 | 1.191e-13 |
| 4+ uORFs | -0.1238 | 9.567e-07 |
| All | -0.145 | 5.795e-27 |

log10 CDS TE against log10 5' leader length

|  | Correlation coefficient | p-value |
| --- | --- | --- |
| 1 uORF | -0.0404 | 0.06464 |
| 2 uORFs | -0.006194 | 0.8352 |
| 3 uORFs | 0.003356 | 0.9313 |
| 4+ uORFs | -0.04079 | 0.1075 |
| All | -0.1129 | 6.469e-17 |

log10 CDS TE against 5' leader mean sec struct EFE

|  | Correlation coefficient | p-value |
| --- | --- | --- |
| 1 uORF | 0.2678 | 2.07e-35 |
| 2 uORFs | 0.2371 | 6.635e-16 |
| 3 uORFs | 0.2721 | 1.022e-12 |
| 4+ uORFs | 0.1132 | 7.551e-06 |
| All | 0.1427 | 4.344e-26 |

log10 CDS TE against log10 5' leader density of uORFs

|  | Correlation coefficient | p-value |
| --- | --- | --- |
| 1 uORF | 0.0404 | 0.06464 |
| 2 uORFs | 0.006194 | 0.8352 |
| 3 uORFs | -0.003356 | 0.9313 |
| 4+ uORFs | 0.01989 | 0.4328 |
| All | -0.06357 | 2.671e-06 |

```
<matplotlib.figure.Figure at 0x3b8bbe10>
```

```
<matplotlib.figure.Figure at 0x1a708860>
```

```
<matplotlib.figure.Figure at 0x3c185a58>
```

```
<matplotlib.figure.Figure at 0x29332390>
```

```
<matplotlib.figure.Figure at 0x29cb88d0>
```

```
<matplotlib.figure.Figure at 0x3c424e10>
```

```
<matplotlib.figure.Figure at 0x2bb1a780>
```

```
<matplotlib.figure.Figure at 0x2a5349e8>
```

```
<matplotlib.figure.Figure at 0x2bd7aeb8>
```

```
<matplotlib.figure.Figure at 0x2969a7b8>
```

```
<matplotlib.figure.Figure at 0x3c272d30>
```

```
<matplotlib.figure.Figure at 0x3c297278>
```

```
<matplotlib.figure.Figure at 0x2b25cb00>
```

```
<matplotlib.figure.Figure at 0x3c35c748>
```

```
<matplotlib.figure.Figure at 0x29bf1f28>
```

```
<matplotlib.figure.Figure at 0x2dad70f0>
```
