# Supplementary material for: Conservation of uORF repressiveness and sequence features in mouse, human and zebrafish
Source: Nat Commun. 2016 May 24;7:11663. doi: 10.1038/ncomms11663 (PMC4890304; doi:10.1038/ncomms11663)
Supplement: Supplementary Software — Conversions of iPython/Jupyter notebooks documenting all analysis for manuscript. Latest versions of iPython/Jupyer notebooks are available at https://github.com/chewgl/uORF_repressiveness_supplemental [file ncomms11663-s2.zip › Fig 4 - Conservation of uORF repressiveness and sequence features.html]

Fig 4 - Conservation of uORF repressiveness and sequence features


# Fig 4 - Conservation of uORF repressiveness and sequence features¶

## Imports, parameters and functions¶

In [1]:

```
# FILTER PARAMETERS
UTR5_LENGTH_MIN = 25
UTR3_LENGTH_MIN = 25

FPKM_MIN = 0.01
CDS_LENGTH_MIN = 100
CDS_READS_MIN = 1

UORF_LENGTH_MIN = 20
UORF_FROM_TRANSCRIPT_START_MIN = UTR5_LENGTH_MIN
UORF_READS_MIN = 1

UTR5_READS_MIN = 1

ORF_END_TRIM = 10
```

In [2]:

```
# IMPORTS
import corebio
import weblogolib

import matplotlib as mpl
import matplotlib.pyplot as plt
import numpy as np
import pandas as pd
import seaborn.apionly as sns

from Bio import SeqIO
from IPython.display import display, HTML, Markdown, Image
from ast import literal_eval
from numpy import argmax, mean, log10, log2, inf, nan, array, float64, subtract, multiply, divide, sign
from pandas import Panel, DataFrame, Series
from pandas.stats.moments import rolling_mean
from scipy.stats import spearmanr, ks_2samp, pearsonr, linregress, zscore, kendalltau, kde
from scipy.stats.mstats import trimboth
from sklearn.linear_model import Ridge, RidgeCV
from statsmodels.distributions import ECDF
from statsmodels.nonparametric.smoothers_lowess import lowess

%matplotlib inline
```

In [3]:

```
pd.options.display.mpl_style = 'default'
pd.options.mode.chained_assignment = None
mpl.rcParams['figure.figsize'] = 8, 6
mpl.rcParams['axes.labelsize'] = 12
mpl.rcParams['axes.titlesize'] = 16
mpl.rcParams['axes.facecolor'] = "#fdfdfd"
mpl.rcParams['grid.alpha'] = 0.5
mpl.rcParams['legend.fontsize'] = 12
mpl.rcParams['font.family'] = 'sans-serif'
mpl.rcParams['figure.autolayout'] = True
mpl.rcParams['savefig.dpi'] = 50
```

In [4]:

```
C_NT = ("#00d700", "#df1f00", "#0226cc", "#ffb700")
C_UORF = ('#b30000', '#e34a33', '#fc8d59', '#fdbb84', '#fdd49e')
C_CDS = ("#105e47", "#157e5f", "#1b9e77", "#48b192", "#76c4ad")
C_RATIO = "#7570b3"
C_BG = ("#000000", "#525252", "#969696", "#d9d9d9")
C_SP = {"hs": "#97543a", "mm": "#546079", "dr": "#3d7463"}
C_SP_BAR = {"hs": "#fc8d62", "mm": "#8da0cb", "dr": "#66c2a5"}
C_NOBIAS = "#ffd92f"
C_SCATTER = "#737373"
C_TREND = "#e41a1c"
C_TLOC = {"5' UTR": "#d95f02", "CDS start": "#1b9e77", "CDS internal": "#e7298a", "3' UTR": "#e6ab02"}
C_STARTS = ("#276419", "#4d9221", "#7fbc41")
C_STOPS = ("#8e0152", "#c51b7d", "#de77ae")
```

In [5]:

```
def flatten(i):
    return [j for k in i for j in k]

def clean(i):
    return i.replace([-inf, inf], nan).dropna()

def z(i):
    return (i - i.mean()) / i.std(ddof=0)
```

In [6]:

```
def scatter_linear_trend(x, y, x_label, y_label, plot_trend=True, corr_table=True):
    data = DataFrame({"x": x, "y": y}).dropna()
    
    plt.figure()
    plt.scatter(data.x, data.y, alpha=0.25, c=C_SCATTER)

    mod = Ridge(normalize=False).fit(zip(*[data.x,]), data.y)
    x1, x2 = (np.percentile(data.x, 2.5), np.percentile(data.x, 97.5))
    y1, y2 = mod.predict([(x1,), (x2,)])
    
    plt.ylim(np.percentile(data.y, 1), np.percentile(data.y, 99))
    plt.xlabel(x_label)
    plt.ylabel(y_label)
    
    if plot_trend:
        plt.plot((x1, x2), (y1, y2), c=C_TREND, lw=2)

    if corr_table:
        to_display = DataFrame(columns=["Correlation\ncoefficient", "p-value"])

        corr, p = pearsonr(data.x, data.y)
        to_display.loc["Pearson linear"] = {"Correlation\ncoefficient": corr,
                                    "p-value": p}
        corr, p = spearmanr(data.x, data.y)
        to_display.loc["Spearman rank"] = {"Correlation\ncoefficient": corr,
                                  "p-value": p}

        display(Markdown(y_label + " against " + x_label))
        display(to_display.applymap('{:,.4g}'.format))
    
    return abs(y2 - y1), sign(y2 - y1)
```

In [7]:

```
def conserve_plot_subset_by_num_uORF(ss, pairs, s_limitss, parameters, para_labels, para_is_logs, species_label):
    for s, pair, limitss in zip(ss, pairs, s_limitss):
        s1, s2 = pair["s1"], pair["s2"]
        set_labels = ["all filtered transcripts",
                      "without uORFs",
                      "with 1-2 uORFs",
                      "with 3+ uORFs"]

        set_filters = [Series({i:True for i in s[s1].index}),
                       ((s[s1]["num_uORFs"] == 0) & (s[s2]["num_uORFs"] == 0)),
                       ((s[s1]["num_uORFs"] >= 1) & (s[s2]["num_uORFs"] >= 1) & \
                        (s[s1]["num_uORFs"] <= 2) & (s[s2]["num_uORFs"] <= 2)),
                       ((s[s1]["num_uORFs"] >= 3) & (s[s2]["num_uORFs"] >= 3))]

        set_colors = (C_CDS[2], C_SCATTER, C_UORF[0], C_UORF[3])
        
        conserve_plot(s, pair, parameters, para_labels, limitss, para_is_logs, s1, s2, set_labels, set_filters, set_colors)
```

In [8]:

```
def conserve_plot_subset_by_uORF_similarity(ss, pairs, s_limitss, parameters, para_labels, para_is_logs, species_label):
    for s, pair, limitss in zip(ss, pairs, s_limitss):
        s1, s2 = pair["s1"], pair["s2"]
        set_labels = ["all filtered transcripts",
                      "similar number of uORFs", "diff number of uORFs",
                      "if uORFs differs"]

        set_filters = [Series({i:True for i in s[s1].index}),
                       (((s[s1]["num_uORFs"] - s[s2]["num_uORFs"]).map(abs) <= 1) & \
                        (s[s1]["num_uORFs"] > 0) & (s[s2]["num_uORFs"] > 0)),
                       (((s[s1]["num_uORFs"] - s[s2]["num_uORFs"]).map(abs) > 1) & \
                        (s[s1]["num_uORFs"] > 0) & (s[s2]["num_uORFs"] > 0)),
                       ((s[s1]["num_uORFs"] == 0) ^ (s[s2]["num_uORFs"] == 0))]

        set_colors = [C_CDS[2], C_UORF[0], C_UORF[2], C_SCATTER]
        
        conserve_plot(s, pair, parameters, para_labels, limitss, para_is_logs, s1, s2, set_labels, set_filters, set_colors)
```

In [9]:

```
def conserve_plot(s, pair, parameters, para_labels, limitss, para_is_logs, s1, s2, set_labels, set_filters, set_colors):

    summary = DataFrame(columns=[set_labels])
    display(Markdown(species_label[s1] + " vs " + species_label[s2]))

    for parameter, para_label, limits, para_is_log in zip(parameters, para_labels, limitss, para_is_logs):
        entry = {i:0 for i in set_labels}
        plt.figure()
        for set_label, set_filter, set_color in zip(set_labels, set_filters, set_colors):
            if para_is_log:
                data = clean(s.minor_xs(parameter)[set_filter].applymap(log10))
            else:
                data = clean(s.minor_xs(parameter)[set_filter])
            if len(data) != 0:
                plt.scatter(data[s1], data[s2], alpha=1, c=set_color, s=10)
                sns.kdeplot(data[s1], data[s2], n_levels=5, linewidths=[0,3,0,0,0], 
                            cmap=None, colors=set_color, alpha=1)
                plt.plot((100, 100), (100, 100), c=set_color, lw=3,
                         label=set_label + " (n=" + str(len(data)) + ")")

                corr, p = pearsonr(data[s1], data[s2])
                append = ""
                if p < 0.05:
                    append += "*"
                if p < 0.01:
                    append += "*"
                if p < 0.001:
                    append += "*"
                entry[set_label] = '{:,.3g}'.format(corr) + append

        summary.loc[para_label] = entry

        if para_is_log:
            legend = plt.legend(title="log10 " + para_label, loc="upper left")
        else:
            legend = plt.legend(title=para_label, loc="upper left")
        plt.setp(legend.get_title(), fontsize=12)
        plt.xlabel(species_label[s1] + " ortholog")
        plt.ylabel(species_label[s2] + " ortholog")
        plt.xlim(*limits[0])
        plt.ylim(*limits[1])
    summary = summary.T
    summary["n"] = Series({set_label: len(s.minor_xs(parameter)[set_filter]) \
                           for set_filter, set_label in zip(set_filters, set_labels)})
    display(summary)
    display(Markdown("\*: p < 0.05; \*\*: p < 0.01; \*\*\*: p < 0.001"))
    print
```

In [10]:

```
def ridge_conservation_z(ss, pairs, to_regress, to_regress_label, regressors, regressor_labels, para_is_logs,
                         limits, limits_coeff, uORF_filter=False, color_coeff=False):

    for s, pair in zip(ss, pairs):
        s1, s2 = pair["s1"], pair["s2"]
        if uORF_filter:
            set_filter = (s[s1]["num_uORFs"] > 0) & (s[s2]["num_uORFs"] > 0)
        
        data = DataFrame()
        for parameter, para_is_log in zip([to_regress,] + regressors, para_is_logs):
            if para_is_log:
                df = clean(s.minor_xs(parameter).applymap(float).applymap(log10)).apply(z)
            else:
                df = clean(s.minor_xs(parameter).applymap(float)).apply(z)
            df.columns = [i + "_" + parameter for i in df.columns]
            if uORF_filter:
                data[df.columns] = df[set_filter]
            else:
                data[df.columns] = df

        div = DataFrame()
        for parameter in [to_regress,] + regressors:
            div[parameter] = data[s1 + "_" + parameter] - data[s2 + "_" + parameter]
        div.dropna(inplace=True)

        mod = Ridge(normalize=False).fit(div[regressors], div[to_regress])
        combined_score = np.sum([div[i].multiply(j) for i, j in zip(regressors, mod.coef_)], axis=0)

        n = str(len(combined_score))
        display(Markdown(species_label[s1] + " vs " + species_label[s2] + " (n=" + n + ")"))

        plt.figure()
        plt.scatter(combined_score, div[to_regress], alpha=0.25, c=C_SCATTER)
        plt.xlabel("Combined divergence of sequence features")
        plt.ylabel("Divergence of " + to_regress_label)
        plt.title(species_label[s1] + " vs " + species_label[s2] + " (n=" + n + ")")

        corr, p = map('{:,.4g}'.format, pearsonr(combined_score, div[to_regress]))

        display(Markdown("Correlation: " + corr + "\np = " + p))

        x1, x2 = [np.percentile(combined_score, i) for i in [2.5, 97.5]]
        predicted = mod.predict(div[regressors])
        y1, y2 = [np.percentile(predicted, i) for i in [2.5, 97.5]]
        plt.plot((x1, x2), (y1, y2), c=C_TREND ,lw=2)
        plt.xlim(*limits[0])
        plt.ylim(*limits[1])

        to_display = DataFrame(columns=["Relative contribution"])
        for i, label, j in zip(regressors, regressor_labels, mod.coef_):
            to_display.loc[label] = {"Relative contribution": j}
        to_display.index.name = "Parameter"
        display(to_display.iloc[::-1].applymap('{:,.4g}'.format))

        plt.figure(figsize=(6, 1 + float(len(to_display)) * .3))
        if color_coeff:
            ax = to_display["Relative contribution"].plot(kind="barh", width=0.9, color=color_coeff)
        else:
            ax = to_display["Relative contribution"].plot(kind="barh", width=0.9)
        ax.set_xlabel("Relative contribution to divergence")
        plt.xlim(*limits_coeff)
        plt.title(to_regress_label)
```

## Reading in orthology data¶

List of directional pair-wise orthologs were downloaded from Ensembl (build 75).

Attributes used: Species 1 Gene, Species 1 Transcript, Species 2 Gene, Species 1 Canonical Protein, Orthology Type, Orthology Confidence

In [11]:

```
ANNOTATIONS_DIR = "./annotations/"
```

In [12]:

```
species = ("mm", "hs", "dr")
species_label = {"mm": "Mouse", "hs": "Human", "dr": "Zebrafish"}
species_stage = {"mm": "mES", "hs": "Hela", "dr": "Shield"}
stage_species = {"mES": "mm", "HeLa": "hs", "Shield": "dr"}
```

In [13]:

```
pairs = [{"s1": "mm", "s2": "hs"},
         {"s1": "mm", "s2": "dr"},
         {"s1": "hs", "s2": "dr"}]
```

Orthology pairs were filtered for high confidence and one2one orthology. Only gene IDs were compiled (duplicates orginating from a gene having multiple transcripts were dropped).

In [14]:

```
ortholog_pairs = DataFrame(index=species, columns=species)

for s1, s2 in [(pair["s1"], pair["s2"]) for pair in pairs]:
    ortholog_file = ANNOTATIONS_DIR + s1 + "-" + s2 + "_orthologs.df"
    orthology = DataFrame.from_csv(ortholog_file, sep="\t", index_col=False)
    orthology = orthology[(orthology["orthology_confidence"] == 1) &
                          (orthology["homology_type"] == "ortholog_one2one")]
    
    
    orthology = pd.concat((orthology[s1 + "_Gene"], orthology[s2 + "_Gene"]), axis=1)
    orthology = orthology.drop_duplicates((s1 + "_Gene", s2 + "_Gene"))
    ortholog_pairs[s1][s2] = orthology.set_index(s1 + "_Gene").squeeze()
```

In [15]:

```
print "Species, Number of Pairs"
for s1, s2 in [(pair["s1"], pair["s2"]) for pair in pairs]:
    print s1, s2, len(ortholog_pairs[s1][s2])
```

```
Species, Number of Pairs
mm hs 9487
mm dr 8240
hs dr 8228
```

## Reading in ORF characteristics data¶

Files were read in as DataFrames into a master DataFrame, filtered for presence in the orthology pairs gene sets.

In [16]:

```
CONVERTERS = {i:literal_eval for i in ("uORFs_reads", "uORFs_length", "uORFs_wrent_score", "uORFs_urent_score",
                                       "uORFs_wrent_seq", "uORFs_sec_struct_EFE_L", "uORFs_sec_struct_EFE_R",
                                       "uORFs_start_pos_wrt_tss", "uORFs_end_pos_wrt_CDS",
                                       "ORFs_wrent_score", "ORFs_urent_score", "ORFs_wrent_seq",
                                       "ORFs_sec_struct_EFE_L", "ORFs_sec_struct_EFE_R")}
```

In [17]:

```
species_df = Series({sp: pd.read_csv("./data/" + sp + "/" + species_stage[sp] + "_main.df",
                                     sep="\t", index_col=["Gene"],
                                     converters=CONVERTERS)
                     for sp in species})
```

Various parameters are calculated here. 5' leader density of ribosome profiling reads was calculated by taking the total number of reads in the 5' leader, divided bythe length of 5' leader.

In [18]:

```
windows = [25, 30, 35, 40]
for i in species:
    mean_ssefes = pd.read_table("./data/" + i + "/" + i + "_mean_ssefes.df", sep="\t", index_col=["Gene"])
    
    for window in windows:
        for pos in ("UTR5", "CDS"):
            field = pos + "_mean_ssefe_" + str(window)
            species_df[i][field] = mean_ssefes[field][mean_ssefes.index.isin(species_df[i].index)]
            species_df[i][field + "_Z"] = z(species_df[i][field])
    
    species_df[i]["CDS_density"] = species_df[i].CDS_reads / (species_df[i].CDS_length - ORF_END_TRIM)
    
    species_df[i]["CDS_TE"] = species_df[i].CDS_density / species_df[i].Gene_Expression_FPKM
    
    species_df[i]["UTR5_density"] = species_df[i].UTR5_reads_trunc / (species_df[i].UTR5_length - ORF_END_TRIM)
    
    species_df[i]["UTR5_TE"] = species_df[i].UTR5_density / species_df[i].Gene_Expression_FPKM
    
    species_df[i]["UTR5_repress"] = species_df[i].UTR5_density / species_df[i].CDS_density
    
    species_df[i]["UTR5_num_uORFs_density"] = species_df[i].num_uORFs / species_df[i].UTR5_length
```

Data is filtered for minimum UTR length and gene expression, as well as having at least one non-overlapping uORF.

In [19]:

```
species_df_filtered = Series({i: species_df[i][(species_df[i].UTR5_length >= UTR5_LENGTH_MIN) &
                                               (species_df[i].UTR3_length >= UTR3_LENGTH_MIN) &
                                               (species_df[i].Gene_Expression_FPKM >= FPKM_MIN) &
                                               (species_df[i].CDS_length >= CDS_LENGTH_MIN) &
                                               (species_df[i].CDS_reads >= CDS_READS_MIN) &
                                               (species_df[i].UTR5_reads_trunc >= UTR5_READS_MIN)]
                              for i in species})
```

Panels are created here for each pairwise comparison.

In [20]:

```
ss = []
for s1, s2 in [(pair["s1"], pair["s2"]) for pair in pairs]:
    s1_to_s2 = ortholog_pairs[s1][s2][(ortholog_pairs[s1][s2].index.isin(species_df_filtered[s1].index)) &\
                                      (ortholog_pairs[s1][s2].isin(species_df_filtered[s2].index))].order()
    
    s1_in_s2 = species_df_filtered[s1].index.isin(s1_to_s2.index)
    s2_in_s1 = species_df_filtered[s2].index.isin(s1_to_s2.values)
    ss.append(Panel({s1: species_df_filtered[s1][s1_in_s2],
                     s2: species_df_filtered[s2][s2_in_s1].sort().set_index(s1_to_s2.index)}))
```

```
c:\Anaconda2\lib\site-packages\ipykernel\__main__.py:3: FutureWarning: order is deprecated, use sort_values(...)
  app.launch_new_instance()
c:\Anaconda2\lib\site-packages\ipykernel\__main__.py:8: FutureWarning: sort(....) is deprecated, use sort_index(.....)
```

## Figures¶

### Conservation of translation efficiency, 5' leader vs CDS translation¶

Positive correlation between density of uORFs in 5' leader between different species.

In [21]:

```
for s, pair in zip(ss, pairs):
    s1, s2 = pair["s1"], pair["s2"]
    set_filter = (s[s1]["num_uORFs"] > 0) & (s[s2]["num_uORFs"] > 0)
    data = clean(s.minor_xs("UTR5_density")[set_filter].applymap(log10))
    
    scatter_linear_trend(data[s1], data[s2],
                         species_label[s1] + " 5' leader uORF number density",
                         species_label[s2] + " 5' leader uORF number density")
    plt.xlim(-3, 1)
    plt.ylim(-2, 0.5)
    plt.title("Filtered transcripts with uORFs")
```

Human 5' leader uORF number density against Mouse 5' leader uORF number density

|  | Correlation coefficient | p-value |
| --- | --- | --- |
| Pearson linear | 0.4439 | 1.431e-78 |
| Spearman rank | 0.4434 | 2.326e-78 |

Zebrafish 5' leader uORF number density against Mouse 5' leader uORF number density

|  | Correlation coefficient | p-value |
| --- | --- | --- |
| Pearson linear | 0.1811 | 2.588e-09 |
| Spearman rank | 0.19 | 3.988e-10 |

Zebrafish 5' leader uORF number density against Human 5' leader uORF number density

|  | Correlation coefficient | p-value |
| --- | --- | --- |
| Pearson linear | 0.1423 | 4.641e-06 |
| Spearman rank | 0.1545 | 6.418e-07 |

```
c:\Anaconda2\lib\site-packages\matplotlib\collections.py:590: FutureWarning: elementwise comparison failed; returning scalar instead, but in the future will perform elementwise comparison
  if self._edgecolors == str('face'):
c:\Anaconda2\lib\site-packages\matplotlib\figure.py:1653: UserWarning: This figure includes Axes that are not compatible with tight_layout, so its results might be incorrect.
  warnings.warn("This figure includes Axes that are not "
```

In [22]:

```
parameters = ["CDS_TE", "UTR5_TE", "UTR5_repress",
              "CDS_density", "UTR5_density", "Gene_Expression_FPKM",
              "UTR5_length", "UTR5_mean_ssefe_35", "CDS_mean_ssefe_35",
              "CDS_wrent_score", "CDS_sec_struct_EFE_R"]
para_labels = ["CDS TE", "5' Leader TE", "(5' Leader TE / CDS TE)",
               "CDS RP read density", "5' leader RP read density", "Transcript expression",
               "5' leader length", "5' leader mean sec struct EFE", "CDS mean sec struct EFE",
               "CDS WRENT score", "CDS start sec struct EFE right"]
para_is_logs = [True, True, True,
                True, True, True,
                True, False, False,
                False, False]
```

In [23]:

```
s_limitss = [[((-2, -0.5), (-3, 0)), ((-3, -0.5), (-3, 0)), ((-1.5, 0.5), (-1.2, 1.2)),
              ((-2, 1.5), (-2.5, 1.5)), ((-2.5, 0.5), (-2, 1)), ((-0.5, 2), (-0.5, 3)),
              ((1.6, 3), (1.6, 3)), ((-15, -4), (-15, -4)), ((-9, -4), (-10, -3)),
              ((-4, 6), (-4, 6)), ((-16, 0), (-16, 0))],
             [((-2, -0.5), (-2.8, -1.2)), ((-3, -0.8), (-3.75, -1)), ((-2, 0.5), (-2, 1)),
               ((-1.4, 1.4), (-2.5, 1)), ((-2, 0.8), (-2.5, 0.3)), ((0, 2.5), (0, 2.5)),
               ((1.4, 3), (1.7, 2.8)), ((-14, -4), (-7, -2)), ((-9, -3), (-7.5, -4)),
               ((-3, 6), (-12, 6)), ((-13, 0), (-13, 0))],
             [((-2.5, -0.3), (-2.5, -1.2)), ((-3.2, -0.2), (-4, -1)), ((-1.5, 1.5), (-2, 1)),
              ((-2, 2), (-2.5, 1.5)), ((-2, 1.5), (-2.5, 0.5)), ((0, 3), (0, 3)),
              ((1.6, 3.2), (1.7, 2.9)), ((-14, -4), (-7, -2)), ((-9, -3), (-7, -4)),
              ((-2, 5), (-12, 5)), ((-15, 0), (-12, 0))]]
```

**Fig 4a-b, Supp Fig 9**: Conservation of TE, 5' leader repressiveness amongst vertebrate orthologous transcripts, and how that varies by the number of uORFs. Translation over CDSes and 5' leaders is generally conserved between mammals, and covaries by the number of uORFs in a transcript.

In [24]:

```
conserve_plot_subset_by_num_uORF(ss, pairs, zip(*zip(*s_limitss)[:6]),
                                 parameters[:6], para_labels[:6], para_is_logs[:6], species_label)
```

Mouse vs Human

```
c:\Anaconda2\lib\site-packages\matplotlib\collections.py:650: FutureWarning: elementwise comparison failed; returning scalar instead, but in the future will perform elementwise comparison
  if self._edgecolors_original != str('face'):
```

|  | CDS TE | 5' Leader TE | (5' Leader TE / CDS TE) | CDS RP read density | 5' leader RP read density | Transcript expression | n |
| --- | --- | --- | --- | --- | --- | --- | --- |
| all filtered transcripts | 0.494\*\*\* | 0.358\*\*\* | 0.515\*\*\* | 0.727\*\*\* | 0.442\*\*\* | 0.6\*\*\* | 3538 |
| without uORFs | 0.495\*\*\* | 0.423\*\*\* | 0.463\*\*\* | 0.691\*\*\* | 0.459\*\*\* | 0.591\*\*\* | 1002 |
| with 1-2 uORFs | 0.496\*\*\* | 0.325\*\*\* | 0.421\*\*\* | 0.665\*\*\* | 0.483\*\*\* | 0.559\*\*\* | 734 |
| with 3+ uORFs | 0.382\*\*\* | 0.427\*\*\* | 0.653\*\*\* | 0.682\*\*\* | 0.484\*\*\* | 0.609\*\*\* | 403 |

\*: p < 0.05; \*\*: p < 0.01; \*\*\*: p < 0.001

```

```

Mouse vs Zebrafish

|  | CDS TE | 5' Leader TE | (5' Leader TE / CDS TE) | CDS RP read density | 5' leader RP read density | Transcript expression | n |
| --- | --- | --- | --- | --- | --- | --- | --- |
| all filtered transcripts | 0.235\*\*\* | 0.128\*\*\* | 0.286\*\*\* | 0.57\*\*\* | 0.177\*\*\* | 0.485\*\*\* | 2220 |
| without uORFs | 0.216\*\*\* | 0.135\* | 0.129\* | 0.478\*\*\* | 0.275\*\*\* | 0.427\*\*\* | 248 |
| with 1-2 uORFs | 0.202\*\*\* | 0.0901 | 0.236\*\*\* | 0.536\*\*\* | 0.127\* | 0.437\*\*\* | 320 |
| with 3+ uORFs | 0.315\*\*\* | 0.197\*\* | 0.356\*\*\* | 0.439\*\*\* | 0.233\*\*\* | 0.398\*\*\* | 240 |

\*: p < 0.05; \*\*: p < 0.01; \*\*\*: p < 0.001

```

```

Human vs Zebrafish

|  | CDS TE | 5' Leader TE | (5' Leader TE / CDS TE) | CDS RP read density | 5' leader RP read density | Transcript expression | n |
| --- | --- | --- | --- | --- | --- | --- | --- |
| all filtered transcripts | 0.231\*\*\* | 0.11\*\*\* | 0.307\*\*\* | 0.526\*\*\* | 0.148\*\*\* | 0.419\*\*\* | 2077 |
| without uORFs | -0.0142 | 0.045 | 0.221\*\*\* | 0.351\*\*\* | 0.189\*\* | 0.415\*\*\* | 220 |
| with 1-2 uORFs | 0.162\*\* | 0.0361 | 0.216\*\*\* | 0.538\*\*\* | 0.107 | 0.386\*\*\* | 305 |
| with 3+ uORFs | 0.275\*\*\* | 0.115 | 0.315\*\*\* | 0.367\*\*\* | 0.119 | 0.208\*\* | 243 |

\*: p < 0.05; \*\*: p < 0.01; \*\*\*: p < 0.001

```

```

**Fig 4c-f**: Conservation of transcript sequence features amongst vertebrate transcripts. Selected sequence features that affect TE are also generally conserved between mammals; while sequence features in the 5' leader (Fig 4c-d) covary by the number of uORFs in the transcript, conservation of sequence features in the CDS are largely independent of number of uORFs (Fig 4e-f)

In [25]:

```
conserve_plot_subset_by_num_uORF(ss, pairs, zip(*zip(*s_limitss)[6:]),
                                 parameters[6:], para_labels[6:], para_is_logs[6:], species_label)
```

Mouse vs Human

|  | 5' leader length | 5' leader mean sec struct EFE | CDS mean sec struct EFE | CDS WRENT score | CDS start sec struct EFE right | n |
| --- | --- | --- | --- | --- | --- | --- |
| all filtered transcripts | 0.452\*\*\* | 0.541\*\*\* | 0.916\*\*\* | 0.652\*\*\* | 0.692\*\*\* | 3538 |
| without uORFs | 0.532\*\*\* | 0.575\*\*\* | 0.921\*\*\* | 0.675\*\*\* | 0.709\*\*\* | 1002 |
| with 1-2 uORFs | 0.57\*\*\* | 0.626\*\*\* | 0.925\*\*\* | 0.734\*\*\* | 0.721\*\*\* | 734 |
| with 3+ uORFs | 0.416\*\*\* | 0.6\*\*\* | 0.93\*\*\* | 0.628\*\*\* | 0.681\*\*\* | 403 |

\*: p < 0.05; \*\*: p < 0.01; \*\*\*: p < 0.001

```

```

Mouse vs Zebrafish

|  | 5' leader length | 5' leader mean sec struct EFE | CDS mean sec struct EFE | CDS WRENT score | CDS start sec struct EFE right | n |
| --- | --- | --- | --- | --- | --- | --- |
| all filtered transcripts | 0.312\*\*\* | 0.0925\*\*\* | 0.34\*\*\* | 0.26\*\*\* | 0.2\*\*\* | 2220 |
| without uORFs | 0.348\*\*\* | 0.167\*\* | 0.392\*\*\* | 0.339\*\*\* | 0.178\*\* | 248 |
| with 1-2 uORFs | 0.309\*\*\* | 0.118\* | 0.354\*\*\* | 0.17\*\* | 0.153\*\* | 320 |
| with 3+ uORFs | 0.309\*\*\* | 0.112 | 0.223\*\*\* | 0.247\*\*\* | 0.138\* | 240 |

\*: p < 0.05; \*\*: p < 0.01; \*\*\*: p < 0.001

```

```

Human vs Zebrafish

|  | 5' leader length | 5' leader mean sec struct EFE | CDS mean sec struct EFE | CDS WRENT score | CDS start sec struct EFE right | n |
| --- | --- | --- | --- | --- | --- | --- |
| all filtered transcripts | 0.198\*\*\* | 0.0423 | 0.268\*\*\* | 0.228\*\*\* | 0.183\*\*\* | 2077 |
| without uORFs | 0.253\*\*\* | 0.121 | 0.332\*\*\* | 0.153\* | 0.18\*\* | 220 |
| with 1-2 uORFs | 0.155\*\* | -0.0557 | 0.297\*\*\* | 0.0817 | 0.185\*\* | 305 |
| with 3+ uORFs | 0.166\*\* | 0.102 | 0.281\*\*\* | 0.221\*\*\* | 0.117 | 243 |

\*: p < 0.05; \*\*: p < 0.01; \*\*\*: p < 0.001

```

```

**Supp Fig 11, Supp Tables 5-7**: Conservation of 5' leader and CDS translation amongst all 3 species, subsetted by whether orthologous transcripts have similar numbers of uORFs.

In [26]:

```
conserve_plot_subset_by_uORF_similarity(ss, pairs, zip(*zip(*s_limitss)[:6]), parameters[:6],
                                        para_labels[:6], para_is_logs[:6], species_label)
```

Mouse vs Human

|  | CDS TE | 5' Leader TE | (5' Leader TE / CDS TE) | CDS RP read density | 5' leader RP read density | Transcript expression | n |
| --- | --- | --- | --- | --- | --- | --- | --- |
| all filtered transcripts | 0.494\*\*\* | 0.358\*\*\* | 0.515\*\*\* | 0.727\*\*\* | 0.442\*\*\* | 0.6\*\*\* | 3538 |
| similar number of uORFs | 0.457\*\*\* | 0.335\*\*\* | 0.503\*\*\* | 0.677\*\*\* | 0.496\*\*\* | 0.571\*\*\* | 1041 |
| diff number of uORFs | 0.516\*\*\* | 0.364\*\*\* | 0.514\*\*\* | 0.686\*\*\* | 0.365\*\*\* | 0.592\*\*\* | 566 |
| if uORFs differs | 0.408\*\*\* | 0.274\*\*\* | 0.363\*\*\* | 0.733\*\*\* | 0.42\*\*\* | 0.589\*\*\* | 929 |

\*: p < 0.05; \*\*: p < 0.01; \*\*\*: p < 0.001

```

```

Mouse vs Zebrafish

|  | CDS TE | 5' Leader TE | (5' Leader TE / CDS TE) | CDS RP read density | 5' leader RP read density | Transcript expression | n |
| --- | --- | --- | --- | --- | --- | --- | --- |
| all filtered transcripts | 0.235\*\*\* | 0.128\*\*\* | 0.286\*\*\* | 0.57\*\*\* | 0.177\*\*\* | 0.485\*\*\* | 2220 |
| similar number of uORFs | 0.271\*\*\* | 0.161\*\*\* | 0.322\*\*\* | 0.547\*\*\* | 0.172\*\*\* | 0.438\*\*\* | 497 |
| diff number of uORFs | 0.165\*\*\* | 0.124\*\* | 0.325\*\*\* | 0.482\*\*\* | 0.181\*\*\* | 0.42\*\*\* | 569 |
| if uORFs differs | 0.152\*\*\* | 0.0434 | 0.105\*\* | 0.53\*\*\* | 0.156\*\*\* | 0.488\*\*\* | 906 |

\*: p < 0.05; \*\*: p < 0.01; \*\*\*: p < 0.001

```

```

Human vs Zebrafish

|  | CDS TE | 5' Leader TE | (5' Leader TE / CDS TE) | CDS RP read density | 5' leader RP read density | Transcript expression | n |
| --- | --- | --- | --- | --- | --- | --- | --- |
| all filtered transcripts | 0.231\*\*\* | 0.11\*\*\* | 0.307\*\*\* | 0.526\*\*\* | 0.148\*\*\* | 0.419\*\*\* | 2077 |
| similar number of uORFs | 0.242\*\*\* | 0.0525 | 0.298\*\*\* | 0.524\*\*\* | 0.113\* | 0.335\*\*\* | 459 |
| diff number of uORFs | 0.277\*\*\* | 0.183\*\*\* | 0.364\*\*\* | 0.479\*\*\* | 0.152\*\*\* | 0.339\*\*\* | 569 |
| if uORFs differs | 0.15\*\*\* | 0.0948\*\* | 0.18\*\*\* | 0.484\*\*\* | 0.153\*\*\* | 0.446\*\*\* | 829 |

\*: p < 0.05; \*\*: p < 0.01; \*\*\*: p < 0.001

```

```

Conservation of sequence features in orthologous transcripts amongst species, subsetted by whether uORFs have similar numbers of uORFs.

In [27]:

```
conserve_plot_subset_by_uORF_similarity(ss, pairs, zip(*zip(*s_limitss)[6:]), parameters[6:],
                                        para_labels[6:], para_is_logs[6:], species_label)
```

Mouse vs Human

|  | 5' leader length | 5' leader mean sec struct EFE | CDS mean sec struct EFE | CDS WRENT score | CDS start sec struct EFE right | n |
| --- | --- | --- | --- | --- | --- | --- |
| all filtered transcripts | 0.452\*\*\* | 0.541\*\*\* | 0.916\*\*\* | 0.652\*\*\* | 0.692\*\*\* | 3538 |
| similar number of uORFs | 0.639\*\*\* | 0.651\*\*\* | 0.925\*\*\* | 0.712\*\*\* | 0.726\*\*\* | 1041 |
| diff number of uORFs | 0.178\*\*\* | 0.319\*\*\* | 0.916\*\*\* | 0.617\*\*\* | 0.616\*\*\* | 566 |
| if uORFs differs | -0.0298 | 0.35\*\*\* | 0.899\*\*\* | 0.579\*\*\* | 0.651\*\*\* | 929 |

\*: p < 0.05; \*\*: p < 0.01; \*\*\*: p < 0.001

```

```

Mouse vs Zebrafish

|  | 5' leader length | 5' leader mean sec struct EFE | CDS mean sec struct EFE | CDS WRENT score | CDS start sec struct EFE right | n |
| --- | --- | --- | --- | --- | --- | --- |
| all filtered transcripts | 0.312\*\*\* | 0.0925\*\*\* | 0.34\*\*\* | 0.26\*\*\* | 0.2\*\*\* | 2220 |
| similar number of uORFs | 0.456\*\*\* | 0.114\* | 0.291\*\*\* | 0.171\*\*\* | 0.13\*\* | 497 |
| diff number of uORFs | 0.122\*\* | 0.0399 | 0.318\*\*\* | 0.255\*\*\* | 0.273\*\*\* | 569 |
| if uORFs differs | 0.0963\*\* | 0.105\*\* | 0.368\*\*\* | 0.277\*\*\* | 0.211\*\*\* | 906 |

\*: p < 0.05; \*\*: p < 0.01; \*\*\*: p < 0.001

```

```

Human vs Zebrafish

|  | 5' leader length | 5' leader mean sec struct EFE | CDS mean sec struct EFE | CDS WRENT score | CDS start sec struct EFE right | n |
| --- | --- | --- | --- | --- | --- | --- |
| all filtered transcripts | 0.198\*\*\* | 0.0423 | 0.268\*\*\* | 0.228\*\*\* | 0.183\*\*\* | 2077 |
| similar number of uORFs | 0.333\*\*\* | -0.00686 | 0.254\*\*\* | 0.106\* | 0.191\*\*\* | 459 |
| diff number of uORFs | 0.0259 | 0.0823 | 0.237\*\*\* | 0.28\*\*\* | 0.185\*\*\* | 569 |
| if uORFs differs | -0.0165 | 0.03 | 0.281\*\*\* | 0.258\*\*\* | 0.18\*\*\* | 829 |

\*: p < 0.05; \*\*: p < 0.01; \*\*\*: p < 0.001

```

```

**Supp Fig S10:** Contribution of transcript expression and CDS TE to the divergence of total gene translation between various vertebrates

For pairwise comparisons, a subset of transcripts with orthologues present in both species was defined.

Log values of transcript levels, CDS RP read density, CDS TEs, and 5' leader TEs were Z-normalized within this subset of transcripts. Divergence was calculated to be the relative difference between the Z-normalized values between species (could be positive or negative depending on which orthologue had the larger Z-normalized score).

A linear model for the divergence of CDS RP read density was constructed using the divergences of CDS TE, 5' leader TE and transcript expression.

In [28]:

```
to_regress, to_regress_label = "CDS_density", "CDS RP read density"
regressors, regressor_labels = (["CDS_TE", "UTR5_TE", "Gene_Expression_FPKM"],
                                ["CDS TE", "5' leader TE", "Transcript expression"])
para_is_logs = [True, True, True, True]

ridge_conservation_z(ss, pairs, to_regress, to_regress_label, regressors, regressor_labels, para_is_logs,
                     ((-4, 4), (-4, 4)), (0, 1))
```

Mouse vs Human (n=3538)

Correlation: 0.9759
p = 0

|  | Relative contribution |
| --- | --- |
| Parameter |  |
| Transcript expression | 0.9041 |
| 5' leader TE | 0.01391 |
| CDS TE | 0.5512 |

Mouse vs Zebrafish (n=2220)

Correlation: 0.9941
p = 0

|  | Relative contribution |
| --- | --- |
| Parameter |  |
| Transcript expression | 0.9163 |
| 5' leader TE | 0.01168 |
| CDS TE | 0.4752 |

Human vs Zebrafish (n=2077)

Correlation: 0.988
p = 0

|  | Relative contribution |
| --- | --- |
| Parameter |  |
| Transcript expression | 0.8442 |
| 5' leader TE | 0.01287 |
| CDS TE | 0.543 |

Determining the relative contribution of transcript expression and 5' leader translational efficiency to 5' leader translation.

In [29]:

```
to_regress, to_regress_label = "UTR5_density", "5' leader RP read density"
regressors, regressor_labels = (["CDS_TE", "UTR5_TE", "Gene_Expression_FPKM"],
                                ["CDS TE", "5' leader TE", "Transcript expression"])
para_is_logs = [True, True, True, True]

ridge_conservation_z(ss, pairs, to_regress, to_regress_label, regressors, regressor_labels, para_is_logs,
                     ((-4, 4), (-4, 4)), (-0.1, 1))
```

Mouse vs Human (n=3538)

Correlation: 0.9913
p = 0

|  | Relative contribution |
| --- | --- |
| Parameter |  |
| Transcript expression | 0.9869 |
| 5' leader TE | 0.919 |
| CDS TE | -0.02756 |

Mouse vs Zebrafish (n=2220)

Correlation: 0.9996
p = 0

|  | Relative contribution |
| --- | --- |
| Parameter |  |
| Transcript expression | 0.9736 |
| 5' leader TE | 0.9084 |
| CDS TE | 0.003328 |

Human vs Zebrafish (n=2077)

Correlation: 0.994
p = 0

|  | Relative contribution |
| --- | --- |
| Parameter |  |
| Transcript expression | 0.9339 |
| 5' leader TE | 0.932 |
| CDS TE | 0.007562 |

### Determining the relative contribution of various sequence features to the divergence of 5' leader repressiveness and CDS TE between species.¶

Sequence features examined are: 5' leader uORF number density, 5' leader mean sec struct EFE, 5' leader length, CDS mean sec struct EFE, CDS start sec struct EFE right, CDS WRENT score

In [30]:

```
regressors, regressor_labels = (["UTR5_num_uORFs_density",
                                 "UTR5_mean_ssefe_35",
                                 "UTR5_length",
                                 "CDS_mean_ssefe_35",
                                 "CDS_sec_struct_EFE_R",
                                 "CDS_wrent_score"],
                                ["5' leader uORF number density",
                                 "5' leader mean sec struct EFE",
                                 "5' leader length",
                                 "CDS mean sec struct EFE",
                                 "CDS start sec struct EFE right",
                                 "CDS WRENT score"])
para_is_logs = [True, True, False, True, False, False, False]
color_pos = (C_TLOC["5' UTR"], C_TLOC["CDS start"])
colors = [color_pos[i] for i in (0, 0, 0, 1, 1, 1)]
```

**Supp Fig 12a-f**: Contribution of sequence features to divergence of 5' leader repressiveness.

In [31]:

```
to_regress, to_regress_label = "UTR5_repress", "5' leader repressiveness"

ridge_conservation_z(ss, pairs, to_regress, to_regress_label, regressors, regressor_labels, para_is_logs,
                     ((-1, 1), (-2.5, 2.5)), (-0.4, 0.2), uORF_filter=True, color_coeff=colors)
```

```
c:\Anaconda2\lib\site-packages\pandas\core\frame.py:1997: UserWarning: Boolean Series key will be reindexed to match DataFrame index.
  "DataFrame index.", UserWarning)
```

Mouse vs Human (n=1601)

Correlation: 0.3368
p = 9.489e-44

|  | Relative contribution |
| --- | --- |
| Parameter |  |
| CDS WRENT score | -0.008611 |
| CDS start sec struct EFE right | 0.005173 |
| CDS mean sec struct EFE | 0.1544 |
| 5' leader length | -0.3531 |
| 5' leader mean sec struct EFE | 0.05529 |
| 5' leader uORF number density | -0.008865 |

Mouse vs Zebrafish (n=1057)

Correlation: 0.2689
p = 5.728e-19

|  | Relative contribution |
| --- | --- |
| Parameter |  |
| CDS WRENT score | 0.03015 |
| CDS start sec struct EFE right | 0.02153 |
| CDS mean sec struct EFE | -0.1198 |
| 5' leader length | -0.1112 |
| 5' leader mean sec struct EFE | 0.1005 |
| 5' leader uORF number density | 0.1243 |

Human vs Zebrafish (n=1015)

Correlation: 0.2911
p = 2.821e-21

|  | Relative contribution |
| --- | --- |
| Parameter |  |
| CDS WRENT score | 0.04796 |
| CDS start sec struct EFE right | 0.0966 |
| CDS mean sec struct EFE | -0.03645 |
| 5' leader length | -0.2451 |
| 5' leader mean sec struct EFE | 0.0257 |
| 5' leader uORF number density | 0.06915 |

**Supp Fig 12g-l**: Contribution of sequence features to divergence of CDS TE.

In [32]:

```
to_regress, to_regress_label = "CDS_TE", "CDS TE"

ridge_conservation_z(ss, pairs, to_regress, to_regress_label, regressors, regressor_labels, para_is_logs,
                     ((-1.25, 1.25), (-3, 3)), (-0.2, 0.4), uORF_filter=True, color_coeff=colors)
```

Mouse vs Human (n=1601)

Correlation: 0.1287
p = 2.371e-07

|  | Relative contribution |
| --- | --- |
| Parameter |  |
| CDS WRENT score | 0.01736 |
| CDS start sec struct EFE right | 0.04848 |
| CDS mean sec struct EFE | 0.2054 |
| 5' leader length | -0.061 |
| 5' leader mean sec struct EFE | 0.07317 |
| 5' leader uORF number density | -0.07652 |

Mouse vs Zebrafish (n=1057)

Correlation: 0.3886
p = 1.933e-39

|  | Relative contribution |
| --- | --- |
| Parameter |  |
| CDS WRENT score | 0.02885 |
| CDS start sec struct EFE right | 0.0636 |
| CDS mean sec struct EFE | 0.3453 |
| 5' leader length | -0.06095 |
| 5' leader mean sec struct EFE | 0.1506 |
| 5' leader uORF number density | -0.1549 |

Human vs Zebrafish (n=1015)

Correlation: 0.4074
p = 7.317e-42

|  | Relative contribution |
| --- | --- |
| Parameter |  |
| CDS WRENT score | -0.02713 |
| CDS start sec struct EFE right | -0.01428 |
| CDS mean sec struct EFE | 0.3852 |
| 5' leader length | -0.02382 |
| 5' leader mean sec struct EFE | 0.115 |
| 5' leader uORF number density | -0.07341 |
