# Supplementary material for: Conservation of uORF repressiveness and sequence features in mouse, human and zebrafish
Source: Nat Commun. 2016 May 24;7:11663. doi: 10.1038/ncomms11663 (PMC4890304; doi:10.1038/ncomms11663)
Supplement: Supplementary Software — Conversions of iPython/Jupyter notebooks documenting all analysis for manuscript. Latest versions of iPython/Jupyer notebooks are available at https://github.com/chewgl/uORF_repressiveness_supplemental [file ncomms11663-s2.zip › 1. Mapping ribosome profiling and RNA-seq data.html]

1. Mapping ribosome profiling and RNA-seq data


# 1. Mapping ribosome profiling and RNA-seq data¶

These steps collate information from transcriptome assemblies, RNA-seq and ribosome profiling raw data to produce ".trpedf" files (details described below) which will be used in all subsequent data preprocessing steps.

## General Software Requirements¶

A \*nix-based system: Bowtie2, Tophat, Cufflinks, Bedtools

Python 2.7 (with Numpy, Scipy, Pandas, ViennaRNA, Statsmodels, Biopython; everything but ViennaRNA can also be run on Windows)

## Raw Data Source¶

Ribosome profiling and RNA-seq data obtained from NCBI's Sequence Read Archive (SRA) by downloading the corresponding .SRA files. Genome and transcriptome annotations were obtained from Illumina iGenomes.

|  |  |  |  |  |
| --- | --- | --- | --- | --- |
| \*\*Sample\*\* | \*\*Ribosome Profiling Data\*\* | \*\*RNA-Seq Data\*\* | \*\*Genome Assembly\*\* | \*\*Transcriptome\*\* |
| Human HeLa cells | SRR970587, SRR970588 | SRR970592, SRR970593 | GRCh37 | Ensembl 70 |
| Mouse ES cells | SRR315616, SRR315617, SRR315618, SRR315619 | SRR315595, SRR315596 | GRCm38 | Ensembl 70 |
| Zebrafish Shield stage | SRR836196 | SRR2047225 | Zv9 | Ensembl 70 |

SRA files were converted to fastq files and clipped of 3' ligation adapter sequences "CTGTAGGCACCATCAAT", retaining reads >= 25 nucleotides.

## Mapping Ribosome Profiling Data¶

Ribosome profiling reads were first depleted of abundent sequences such as rRNA using Bowtie2. Abundant sequences were compiled from the AbundantSequences directory from Illumina iGenomes, and built into a Bowtie2 index. Additional manually-curated rRNA sequences for zebrafish were used, and are included in the supplementary data files.

Example using Mouse ES cell ribosome profiling data below:

In [ ]:

```
%%bash
cat *.fa > ./annotations/GRCm38_Abundant.fa
bowtie2-build ./annotations/GRCm38_Abundant.fa GRCm38_Abundant

OPTIONS="-N 0 -L 23 --norc"
bowtie2 $OPTIONS --un ribo_mES_sub_abund.fastq -x GRCm38_Abundant -U ribo_mES.fastq -S /dev/null
```

Remaining reads were mapped to the Ensembl 70 transcriptome using Tophat, allowing no indels, junctions only from gene annotations, max 10 multihits, with multihit pre-filtering. Use the .gtf files and Bowtie2 indices ("genome") from the corresponding iGenomes compilation.

In [ ]:

```
%%bash
OPTIONS="--max-insertion-length 0 --max-deletion-length 0\
--no-novel-juncs -g 10 --prefilter-multihits\
--library-type fr-secondstrand"
tophat -o ribo_mES $OPTIONS -G genes.gtf genome ribo_mES_sub_abund.fastq
```

## Mapping RNA-Seq Data¶

RNA-seq reads (when libraries were constructed by 3' ligation) were mapped by Tophat using the following parameters:

In [ ]:

```
%%bash
OPTIONS="--no-novel-juncs --library-type=fr-secondstrand"
tophat -o mRNA_mES $OPTIONS -G genes.gtf genome mRNA_mES.fastq
```

Quantification of RNA-seq data was done using Cufflinks; accepted\_hits.bam is from the Tophat output:

In [ ]:

```
%%bash
OPTIONS="-b genome.fa --multi-read-correct --library-type=fr-secondstrand"
cufflinks ${OPTIONS} -o cuffdiff/ -G genes.gtf accepted_hits.bam
```

## Assembling Canonical Transcriptome¶

To generate a list of transcripts that only use one "canonical" transcript isoform per gene, ensGtp tables for each vertebrate species were retrieved from the UCSC genome browser. BED files were generated from the refFlat files in the iGenomes compilation, using the following awk script:

In [ ]:

```
# ASSEMBLY="GRCm38_ens"
# Save following script as file [refFlat_to_bed12.awk]
# run as 'refFlat_to_bed12.awk refFlat.txt > ./annotations/$ASSEMBLY_genes.bed'

#!/bin/awk -f
BEGIN  {FS="\t"; OFS="\t"}
{   blockSizes="";
    blockStarts="";
    split($10,exonStarts,",");
    split($11,exonEnds,",");
    
    for (i=1; i<=$9; i++)
    {   blockSizes=blockSizes exonEnds[i]-exonStarts[i] ",";
    blockStarts=blockStarts exonStarts[i]-$5 ",";
    }
    blockSizes = substr(blockSizes,1,length(blockSizes)-1);
    blockStarts = substr(blockStarts,1,length(blockStarts)-1);
    print $3,$5,$6,$2,0,$4,$7,$8,"0,0,0",$9,blockSizes,blockStarts;
}
```

Canonical transcriptome contain one transcript per gene: the transcript with the longest CDS, then longest 5' UTR, then longest transcript length.

With the BED file and ensGtp file in the same directory, the following python script was run to generate "\$ASSEMBLY\_genes\_canonical.bed", which is the transcript subset of \$ASSEMBLY\_genes.bed with one transcript per gene.

Upload the file to UCSC as a custom track and use it to obtain the corresponding fasta file. Alternatively, the transcriptome fasta file can be obtained from a local whole genome fasta file using bedtools getfasta (a.k.a. getFastaFromBed)

In [ ]:

```
ANNOTATIONS_DIR = "./annotations/"
DATA_DIR = "./data/"
ASSEMBLY = "GRCm38_ens"

def transcript_position(exons, c_intron_lengths, genomic_pos):
    for exon, c_intron_length in zip(exons, c_intron_lengths):
        if genomic_pos >= exon[0] and genomic_pos <= exon[1]:
            return genomic_pos - exons[0][0] - c_intron_length

def read_Gtp_file(Gtp_file):
    transcript_to_gene = {}
    gene_to_transcript = {}
    with open(Gtp_file, "r+") as f:
        for line in f:
            entry = line.strip().split("\t")
            transcript_to_gene[entry[1]] = entry[0]
            gene_to_transcript.setdefault(entry[0], []).append(entry[1])
    return transcript_to_gene, gene_to_transcript
        
#%% OPEN FILES

with open(ANNOTATIONS_DIR + ASSEMBLY + "_genes.bed", "r+") as in_bed, \
open(ANNOTATIONS_DIR + ASSEMBLY + "_genes_canonical.bed", "w+") as out_bed:

    #%% READ GTP FILE
    transcript_to_gene, gene_to_transcript = read_Gtp_file(ANNOTATIONS_DIR + ASSEMBLY + "Gtp")
    
    #%% READ BED FILES
    bed_store = {}
    for line in in_bed:
        _, chromStart, chromEnd, name, \
        _, strand, thickStart, thickEnd, \
        _, blockCount, blockSizes, blockStarts = line.split("\t")
        if name not in transcript_to_gene:
            continue

        #%% CONVERT ENTRY TO INTEGERS
        chromStart, chromEnd, thickStart, thickEnd, blockCount = map(int,
                                                                     (chromStart, chromEnd,
                                                                      thickStart, thickEnd, blockCount))
        blockSizes = map(int, blockSizes.split(","))
        blockStarts = map(int, blockStarts.split(","))

        #%% SECONDARY DATA FOR CALCULATIONS
        intron_lengths = [(blockStarts[i+1]-blockStarts[i]-blockSizes[i]) for i in xrange(blockCount-1)]
        c_intron_lengths = [sum(intron_lengths[:i]) for i in xrange(blockCount)]
        exons = [[i[0] + chromStart, sum(i) + chromStart] for i in zip(blockStarts, blockSizes)]
        plus_strand = (strand == "+")

        #%% CALCULATE LENGTHS: TRANSCRIPT, 5'LEADER, CDS, 3'UTR
        transcript_length = sum(blockSizes)
        if plus_strand:
            UTR5_length = abs(transcript_position(exons, c_intron_lengths, thickStart)\
                              - transcript_position(exons, c_intron_lengths, chromStart))
            UTR3_length = abs(transcript_position(exons, c_intron_lengths, chromEnd)\
                              - transcript_position(exons, c_intron_lengths, thickEnd))
        else:
            UTR5_length = abs(transcript_position(exons, c_intron_lengths, chromEnd)\
                              - transcript_position(exons, c_intron_lengths, thickEnd))
            UTR3_length = abs(transcript_position(exons, c_intron_lengths, thickStart)\
                              - transcript_position(exons, c_intron_lengths, chromStart))
        CDS_length = abs(transcript_position(exons, c_intron_lengths, thickEnd)\
                         - transcript_position(exons, c_intron_lengths, thickStart))
        bed_store[name] = [line, CDS_length, UTR5_length, transcript_length]

    #%% FIND TRANSCRIPT WITH LONGEST CDS, THEN LONGEST 5' LEADER, THEN LONGEST TRANSCRIPT LENGTH, PER GENE, OUTPUT
    for gene in gene_to_transcript:
        try:
            canonical_transcript = sorted([[transcript, bed_store[transcript][1],
                                            bed_store[transcript][2],
                                            bed_store[transcript][3]] \
                                           for transcript in gene_to_transcript[gene] \
                                           if transcript in bed_store], key=lambda i: (i[1], i[2], i[3]))[-1][0]
        except IndexError:
            continue
        out_bed.write(bed_store[canonical_transcript][0])
```

## Integrating ribosome profiling, RNA-seq data in transcript coordinates¶

For data analysis, a custom file-format is used that integrates RNA-seq and ribosome profiling data in the context of a defined transcriptome.

Ribosome profiling data first needs to be assembled at nucleotide resolution. Note that the offsets correspond to P-site, rather than A-site.

Use either of the following awk scripts as part of the conversion of .bam files (accepted\_hits.bam from Tophat).

In [ ]:

```
# Create file as bed12_to_bedpoint_mammal.awk, for use with human and mouse ribosome profiling data

#!/bin/awk -f
BEGIN {OFS="\t"}

{if ($10 != 1){
    split($11,a,",");\
    split($12,b,",");\
    len=0;\
    for (i in a){len+= a[i]}
}
else
    {len=$11}

out=(len>=29 && len<=35);\
strand=$6;\
if (out){
if(strand=="+"){
         if(len == 29) offset = 12;\
    else if(len == 30) offset = 12;\
    else if(len == 31) offset = 13;\
    else if(len == 32) offset = 13;\
    else if(len == 33) offset = 13;\
    else if(len == 34) offset = 14;\
    else if(len == 35) offset = 14;\
    }
else{    if(len == 29) offset = 16;\
    else if(len == 30) offset = 17;\
    else if(len == 31) offset = 17;\
    else if(len == 32) offset = 18;\
    else if(len == 33) offset = 19;\
    else if(len == 34) offset = 19;\
    else if(len == 35) offset = 20;\
    }
}

if(out && ($10 == 1)){print $1, $2+offset, $2+offset+1, $4, $5, $6}
else if(out){
    for (i in a){
        if (offset <= a[i] && offset > 0){print $1, $2+offset+b[i], $2+offset+b[i]+1, $4, $5, $6}
        offset -= a[i];\
    }
}
}
```

In [ ]:

```
# Create file as bed12_to_bedpoint_zf.awk, for use with zebrafish ribosome profiling data

#!/bin/awk -f
BEGIN {OFS="\t"}

{if ($10 != 1){
    split($11,a,",");\
    split($12,b,",");\
    len=0;\
    for (i in a){len+= a[i]}
}
else
    {len=$11}

out=(len>=27 && len<=32);\
strand=$6;\
if (out){
if(strand=="+"){
         if(len == 27) offset = 11;\
    else if(len == 28) offset = 11;\
    else if(len == 29) offset = 12;\
    else if(len == 30) offset = 12;\
    else if(len == 31) offset = 12;\
    else if(len == 32) offset = 13;\
    }
else{    if(len == 27) offset = 15;\
    else if(len == 28) offset = 16;\
    else if(len == 29) offset = 16;\
    else if(len == 30) offset = 17;\
    else if(len == 31) offset = 18;\
    else if(len == 32) offset = 18;\
   }
}

if(out && ($10 == 1)){print $1, $2+offset, $2+offset+1, $4, $5, $6}
else if(out){
    for (i in a){
        if (offset <= a[i] && offset > 0){print $1, $2+offset+b[i], $2+offset+b[i]+1, $4, $5, $6}
    offset -= a[i];\
    }
}
}
```

Running the following commands (from BedTools) generates the ".in" files that will be used for creating the ".trpedf" files in subsequent analyses, as well as strand-specific bedgraph files (can be converted to binary .bw files using bedgraphToBigWig from UCSC, for easy viewing in most genome browsers). ".bg.bed" files may be deleted following execution of these commands. The respective "ChromInfo.txt" files can be found in the iGenomes compilations.

In [ ]:

```
%%bash
ASSEMBLY="GRCm38_ens"
bamToBed -bed12 -i accepted_hits.bam | bed12_to_bedpoint_mammal.awk |\
    tee >(genomeCoverageBed -bg -i stdin -g ChromInfo.txt -strand + > mES_fwd.bedgraph) \
        >(genomeCoverageBed -bg -i stdin -g ChromInfo.txt -strand - > mES_rev.bedgraph) \
        >/dev/null

awk 'BEGIN{FS="\t";OFS="\t"}{print $1,$2,$3,".",$4,"+"}' mES_fwd.bedgraph > mES_fwd.bg.bed
awk 'BEGIN{FS="\t";OFS="\t"}{print $1,$2,$3,".",-$4,"-"}' mES_rev.bedgraph > mES_rev.bg.bed

cat mES_fwd.bg.bed mES_rev.bg.bed | sort -k1,1 -k2,2n > mES.bg.bed
intersectBed -wa -wb -s -split -a $ASSEMBLY_genes_canonical.bed -b mES_rev.bg.bed | \
awk 'BEGIN{FS="\t"; OFS="\t"}\
    {if ($4==curr) print $14,$15,$17;\
    else {print $1,$2,$3,$4,$5,$6,$7,$8,$9,$10,$11,$12; print $14,$15,$17; curr=$4}}'\
> mES_canonical.in
```

The following python script integrates mRNA expression data from cufflinks (genes.fpkm\_tracking files from the cufflinks output), ribosome profiling data from ".in" files, and sequence data (from genes\_canonical.fasta, derived from genes\_canonical.bed; to define all ORFs).

gene\_canonical.fasta file can be generated from genes\_canonical.bed either by uploading the .bed file to UCSC and downloading the fasta, or by using Bedtools getfasta.

Data was organized in a tab-separated custom ASCII file format (.trpedf) for subsequent processing.
(N.B. trpedf ~ **t**ranscript **r**ibosome **p**rofile **e**xtended, **D**ata**F**rame compatible)

|  |  |
| --- | --- |
| \*\*Column\*\* | \*\*Description\*\* |
| Transcript | Transcript ID |
| Gene | Gene ID |
| Gene\_Name | Gene Name |
| Gene\_Expression\_FPKM | Expression at gene level (from corresponding RNA-seq data; Tophat + Cufflinks) |
| ORF\_starts | ORF starts (comma-separated values in transcript coordinates, 0-based) |
| ORF\_ends | ORF ends (as above) |
| RPF\_csvProfile | Ribosome profiling reads at nucleotide resolution in transcript coordinates, for length of transcript, comma-separated values |
| CDS | Annotated CDS |

".in" files may be deleted following execution of these commands.

In [ ]:

```
ASSEMBLY = "GRCm38_ens"
stage = "mES"

from Bio import SeqIO
from ast import literal_eval
import hmm_for_RPF_Seq as h

def read_genes_tracking_file(tracking_file, stages):
    expected_line_length = 9 + 4 * len(stages)
    ensg_expression = {stage:{} for stage in stages}
    ensg_name = {}
    with open(tracking_file, "r+") as f:
        for line in f:
            entry = line.strip().split("\t")
            if entry[0] == "tracking_id" or len(entry) != expected_line_length: continue
            for i, stage in enumerate(stages):
                expression = 4 * i + 9
                status = 4 * i + 12
                if entry[status] != "OK": continue
                ensg_expression[stage][entry[0]] = float(entry[(expression)])
            ensg_name[entry[0]] = entry[4]
        return ensg_expression, ensg_name

def read_Gtp_file(Gtp_file):
    transcript_to_gene = {}
    gene_to_transcript = {}
    with open(Gtp_file, "r+") as f:
        for line in f:
            entry = line.strip().split("\t")
            transcript_to_gene[entry[1]] = entry[0]
            gene_to_transcript.setdefault(entry[0], []).append(entry[1])
    return transcript_to_gene, gene_to_transcript

def csv(list):
    return ",".join(map(str, list))

def tsv_line(*list):
    return "\t".join(map(str, list)) + "\n"

def ORF_start_end(seq):
    ORF_list = []
    seq_len = len(seq)
    for frame in xrange(3):
        trans = str(seq[frame:].translate(1))
        trans_len = len(trans)
        aa_start, aa_end = [0 for i in xrange(2)]
        while aa_start < trans_len:
            aa_start = trans.find("M", aa_start)
            if aa_start == -1:
                break
            aa_end = trans.find("*", aa_start)
            ORF_start = frame + aa_start * 3
            ORF_end = frame + aa_end * 3 + 3
            if aa_end == -1:
                ORF_end = seq_len
            ORF_list.append((ORF_start, ORF_end))
            aa_start = aa_start + 1
    return zip(*tuple(sorted(ORF_list)))

def transcript_position(exons, c_intron_lengths, genomic_pos):
    for exon, c_intron_length in zip(exons, c_intron_lengths):
        if genomic_pos >= exon[0] and genomic_pos <= exon[1]:
            return genomic_pos - exons[0][0] - c_intron_length
    return

def parse_in_file(f, prev_entry_pos):
    f.seek(prev_entry_pos)
    while 1:
        line = f.readline()
        entry = line.split()
        if len(entry) != 3:
            try:
                if thick_start == thick_end: transcript_CDS = [0,0]
                else:
                    # assume strand is "+" first
                    transcript_CDS = [transcript_position(exons, c_intron_lengths, thick_start),
                                      transcript_position(exons, c_intron_lengths, thick_end)]
                    if strand == "-":
                        transcript_bedgraph.reverse()
                        transcript_CDS[0], transcript_CDS[1] = (transcript_length - transcript_CDS[1],
                                                                transcript_length - transcript_CDS[0])
                return transcript_ID, transcript_CDS, transcript_bedgraph, prev_entry_pos
            except UnboundLocalError:
                pass
            transcript_ID, strand = (entry[3], entry[5])
            transcript_start, thick_start, thick_end, block_count = map(int, (entry[1], entry[6], entry[7], entry[9]))

            block_sizes = literal_eval(entry[10])
            genome_block_starts = literal_eval(entry[11])
            
            transcript_length = sum(block_sizes)
            transcript_bedgraph = [0] * transcript_length
            
            #introns and exons below in *GENOMIC* coordinates (i.e. not strand-specific)
            intron_lengths = [(genome_block_starts[i+1]-genome_block_starts[i]-block_sizes[i]) for i in xrange(block_count-1)]
            
            c_intron_lengths = [sum(intron_lengths[:i]) for i in xrange(block_count)]
            exons = [[i[0], sum(i)] for i in zip(genome_block_starts, block_sizes)]
            prev_entry = entry
        else:
            prev_entry_pos = f.tell()
            transcript_pos = transcript_position(exons, c_intron_lengths, int(entry[1]))
            if transcript_pos != None:
                for i in xrange(int(entry[1])-int(entry[0])):
                    if transcript_pos + i < transcript_length:
                        transcript_bedgraph[transcript_pos + i] = abs(int(entry[2]))

#%% Files, Stages

ensg_expression, ensg_name = read_genes_tracking_file(DATA_DIR + stage + "_genes.fpkm_tracking", stages)
seqs = SeqIO.index(ANNOTATIONS_DIR + ASSEMBLY + "_genes_canonical.fasta", "fasta")
enst_to_ensg, ensg_to_enst = read_Gtp_file(ANNOTATIONS_DIR + ASSEMBLY + "Gtp")

in_file = stage + ".in"
trpedf_file = DATA_DIR + stage + "_canonical.trpedf"

#%% DEFINE ORFs in seqs
ORF_starts_ends = {}
for seq in seqs:
    ORF_starts_ends[seq] = ORF_start_end(seqs[seq].seq)

with open(in_file, 'rb+') as f, open(trpedf_file, 'w+') as out:
    out.write(tsv_line("Transcript", "Gene", "Gene_Name", "Gene_Expression_FPKM",
                       "ORF_starts", "ORF_ends", "RPF_csvProfile", "CDS"))
    prev_entry_pos = 0
    while 1:
        try:
            ID,transcript_CDS, transcript_bedgraph, prev_entry_pos = parse_in_file(f, prev_entry_pos)
        except IndexError:
            break
        try:
            expression = ensg_expression[stage][enst_to_ensg[ID]]
        except KeyError:
            continue
        try:
            name = ensg_name[enst_to_ensg[ID]]
        except KeyError:
            name = enst_to_ensg[ID]
        if len(ORF_starts_ends[ID]) == 0: continue
        out.write(tsv_line(ID, enst_to_ensg[ID], name, expression,
                           csv(ORF_starts_ends[ID][0]),
                           csv(ORF_starts_ends[ID][1]),
                           csv(transcript_bedgraph),
                           csv(transcript_CDS)))
```
